# Efficient Ligand Discovery Using Sulfur(VI) Fluoride Reactive Fragments

**DOI:** 10.1021/acschembio.3c00034

**Published:** 2023-04-21

**Authors:** Arron Aatkar, Aini Vuorinen, Oliver E. Longfield, Katharine Gilbert, Rachel Peltier-Heap, Craig D. Wagner, Francesca Zappacosta, Katrin Rittinger, Chun-wa Chung, David House, Nicholas C. O. Tomkinson, Jacob T. Bush

**Affiliations:** † GSK, Gunnels Wood Road, Stevenage, Hertfordshire SG1 2NY, U.K.; ‡ Department of Pure and Applied Chemistry, University of Strathclyde, 295 Cathedral Street, Glasgow G1 1XL, U.K.; § The Francis Crick Institute, London NW1 1AT, U.K.; ∥ GSK, South Collegeville Road, Collegeville, Pennsylvania 19426, United States

## Abstract

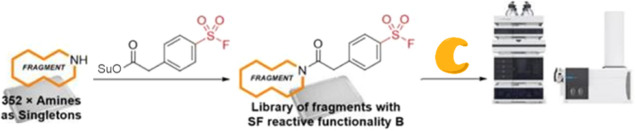

Sulfur(VI) fluorides (SFs) have emerged as valuable electrophiles for the design of “beyond-cysteine” covalent inhibitors and offer potential for expansion of the liganded proteome. Since SFs target a broad range of nucleophilic amino acids, they deliver an approach for the covalent modification of proteins without requirement for a proximal cysteine residue. Further to this, libraries of reactive fragments present an innovative approach for the discovery of ligands and tools for proteins of interest by leveraging a breadth of mass spectrometry analytical approaches. Herein, we report a screening approach that exploits the unique properties of SFs for this purpose. Libraries of SF-containing reactive fragments were synthesized, and a direct-to-biology workflow was taken to efficiently identify hit compounds for CAII and BCL6. The most promising hits were further characterized to establish the site(s) of covalent modification, modification kinetics, and target engagement in cells. Crystallography was used to gain a detailed molecular understanding of how these reactive fragments bind to their target. It is anticipated that this screening protocol can be used for the accelerated discovery of “beyond-cysteine” covalent inhibitors.

## Introduction

**Figure 1:**
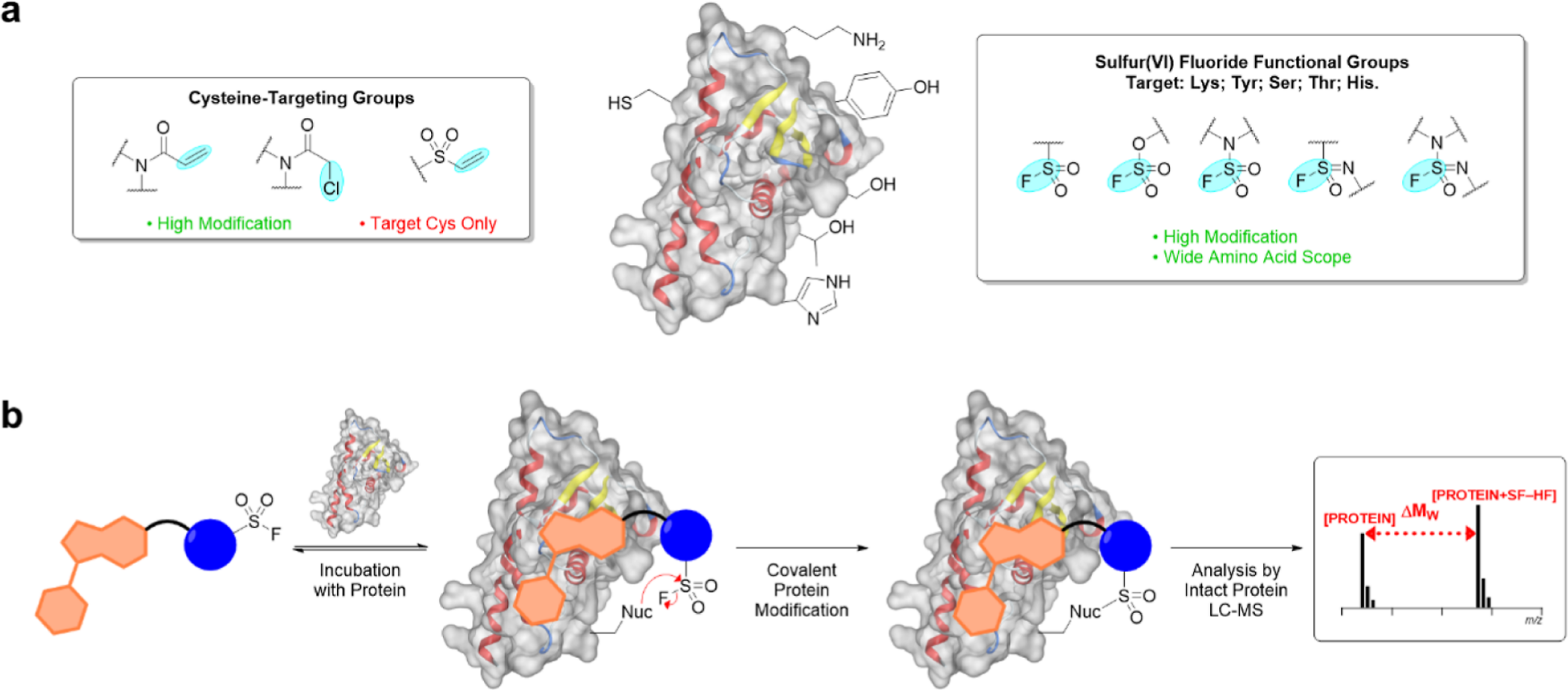
Approaches to covalent protein modification in chemical biology and drug discovery. (a) Summary of electrophiles commonly used for the covalent modification of proteins, including cysteine-targeting reactive groups and SF functional groups. (b) Schematic representation of the mechanism associated with an SF-containing ligand targeting a nucleophilic amino acid residue and subsequent read-out by intact protein LC–MS.

The impact of cysteine-targeting covalent modifiers has spurred interest in the development of complementary “beyond-cysteine” approaches to target additional amino acid residues and thus expand applicability across the proteome (Figure 1a).^1−5^ Sulfur(VI) fluorides (SFs) have emerged as useful electrophiles for this application, targeting multiple nucleophilic amino acid residues, including lysine,^6^ tyrosine,^7^ and serine.^8^ The prevalence of these residues in almost all protein pockets makes SFs promising functional groups for the development of covalent inhibitors for proteins and expansion of the liganded proteome.^9−16^ Recently, several SF-containing modulators have been reported, which enabled covalent modification of protein pockets without targeting a cysteine. Examples include “XO44” for broad-spectrum kinase profiling,^17^ various SF-containing ligands for targeting G protein-coupled receptors including the human adenosine A_3_ receptor,^18^ and “EM12-SO_2_F”/“EM12-FS” which modulate cereblon.^19^ These were developed by structure-based, rational installation of the SF group on optimized noncovalent scaffolds. The development of complementary “bottom-up” approaches will be useful for discovering ligands for targets that have low tractability to noncovalent ligands.^20^

Reactive fragment screening has emerged as a useful strategy for the discovery of chemical probes for protein targets of interest.^21^ These approaches couple the utility of fragments in enabling the efficient coverage of chemical space, with a reactive functionality that traps weak protein–ligand interactions to improve binding, and enable robust detection by intact protein liquid chromatography–mass spectrometry (LC–MS) (Figure 1b).^22,23^ Covalent capture provides access to a suite of follow-up studies that further characterize the interaction, including determination of the site(s) of binding, measurement of kinetic parameters, and assessment of in-cell target engagement.^3,24,25^ To date, the approach has been limited to cysteine-targeting covalent inhibitors for challenging targets, including HOIP and KRAS^G12C^, which led to the discovery of the Food and Drug Administration-approved therapeutic AMG 510.^3,26^ Chemistries that enable the screening of electrophilic libraries to target alternative nucleophilic residues would greatly expand the number of proteins that are amenable to reactive fragment screening technologies.

Here, we report an SF-reactive fragment screening approach for the identification of covalent ligands for proteins of interest. This approach enabled the rapid discovery of novel ligands for multiple protein pockets without reliance upon the presence of a cysteine residue. This strategy employed a high-throughput chemistry direct-to-biology (HTC-D2B) workflow, providing an expedient and accessible method for the rapid and iterative generation of SF-reactive fragment libraries.^27−29^

## Results and Discussion

**Figure 2:**
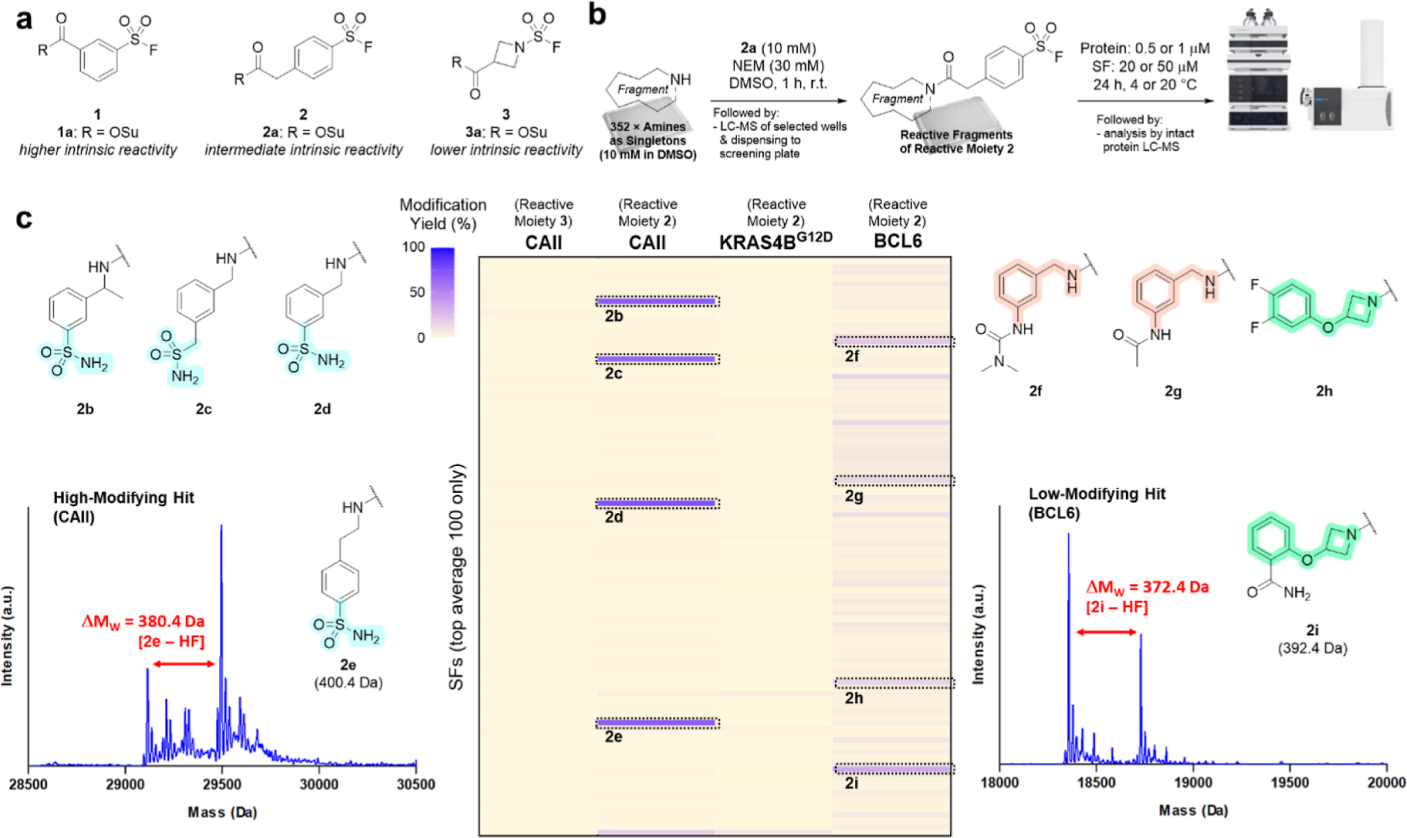
HTC-D2B protocol identifies hit compounds following screens against purified proteins. (a) Structures of SF-reactive moieties **1**–**3** considered for the screening approach. (b) Overview of the high-throughput coupling of amine-functionalized fragments with the SF moiety and subsequent screening of crude reaction mixtures against proteins of interest. (c) Heatmap summary of SF fragment screens against CAII, KRAS4B^G12D^, and BCL6, with hit structures **2b**–**e** and **2f**–**i** shown alongside exemplar mass spectra. Screening conditions: [protein] = 0.5 μM for CAII, 1 μM for KRAS4B^G12D^ and BCL6, [SF] = 20 μM, 24 h, 20 °C (4 °C for BCL6).

The SF-based reactive fragment screening approach was developed in two stages: first, the development of an appropriate SF fragment library and second, the application of this library in screens against three protein targets. A modular library was employed by linking a diverse set of amine-functionalized fragments to an SF-containing reactive moiety. Three SFs were selected that spanned a range of intrinsic reactivities, based on previous profiling of SF functionalities.^14^ These included aromatic sulfonyl fluorides **1** (meta-substituted) and **2** (para-substituted with a methylene spacer), as well as sulfamoyl fluoride **3** (azetidine-linked) (Figure 2a).

### HTC Synthesis of an SF-Reactive Fragment Library

An HTC protocol for the generation of the reactive fragment libraries was pursued to enable the rapid generation of SF fragments in 384-well plates. This allowed for screening in a D2B format as crude reaction mixtures, circumventing the requirement for purification and thus accelerating reactive fragment library screens.^29^

A succinimide-activated (OSu) amide coupling was employed using dimethyl sulfoxide (DMSO) and *N*-ethylmorpholine (NEM) as the solvent and base, respectively.^29^ The conditions were initially trialed on a panel of 12 diverse amine-functionalized fragments by the addition of SF-reactive moieties (**1a**, **2a**, and **3a**) in both dry DMSO and DMSO/water (9:1) to assess robustness to hydrolysis under the reaction conditions. After 1 h incubation, reactions with OSu esters **2a** and **3a** afforded good conversion to the desired products and tolerance of 10% water, while meta-substituted OSu ester **1a** gave poorer conversions due to hydrolysis of the SF group to the sulfonic acid (see Figures S1 and S2).^14^ Thus, compounds **2a** and **3a** were selected for library synthesis. Over longer incubation times, the SF products were observed to slowly hydrolyze to the sulfonic acids under the basic reaction conditions. As such, library synthesis was performed using a 1 h reaction incubation time, followed by direct transfer to protein-containing buffer solution to limit base catalyzed hydrolysis of the SF fragments.

Two 352-membered SF-reactive fragment libraries were subsequently synthesized employing para-substituted reactive moiety **2** and azetidine-linked reactive moiety **3**. A set of 352 amine-functionalized fragments were selected from the GSK compound collection by first filtering for fragment-like properties (aromatic ring count ≤2; HBDs/HBAs ≤4; heavy atoms ≤ 15; 150 < *M*_W_ ≤ 250) and then selecting for maximal chemical diversity by clustering on chemical fingerprints (see Figure S3).^30^ Owing to the slow hydrolysis of the products under basic reaction conditions, it was not possible to perform LC–MS analysis of the whole plate, which would require ∼24 h. Thus, LC–MS analysis was performed on six wells selected at random, which indicated good conversion to the desired products, and conversions were found to be highly reproducible across three library syntheses conducted on separate occasions (see Figure S4). The high yields and reproducibility observed were consistent with related HTC-D2B protocols developed in our group employing photoreactive fragments.^29^

### D2B Screening against a Range of Purified Proteins

A panel of three proteins were selected for screening SF-reactive fragment libraries: CAII, KRAS4B^G12D^, and BCL6. These proteins were selected to sample broad structural diversity and biological function while also being of therapeutic relevance. None of the proteins contain a catalytic nucleophilic amino acid residue, which allowed us to probe the utility of the SFs in targeting nucleophilic amino acid residues present in the vicinity of binding pockets.

Initially, the more reactive library, containing para-substituted aryl sulfonyl fluoride **2**, was screened against the three proteins (24 h incubation, 4 or 20 °C) and directly analyzed by intact protein LC–MS (0.5 or 1 μM protein and 20 or 50 μM SF) (Figure 2b). Across all screens, the majority of wells contained unmodified protein, indicating that the SF-reactive moieties were not yielding nonspecific covalent modifications. However, for some of the wells, the resultant mass spectra displayed additional peaks with mass shifts consistent with the covalent modification of the protein by the SF fragment, accompanied by the loss of HF as expected for the reaction between the nucleophilic amino acid residue with the SF group: [protein + SF – HF]. A range of modification yields were observed, and the hit threshold for each screen was defined as the mean percentage modification + 2 standard deviations. Hits were also prioritized based on the overall extent of modification, with some showing modification greater than 50% (e.g., **2b**–**e** with CAII), while others showed modification less than 50% (e.g., **2f**–**i** with BCL6) (Figure 2c). All hits gave a single modification event on the protein, consistent with recognition-driven modification. Hits containing reactive moiety **2** from the screens against CAII and BCL6 were resynthesized and purified for use in further investigations. Disappointingly, no hits were observed for KRAS^G12D^, consistent with the fact that this is considered to be a poorly tractable target.^25^ The library containing the less-reactive sulfamoyl fluoride **3** was screened against CAII but afforded no hits, suggesting an insufficient intrinsic reactivity of the electrophile.

### Site(s) of Binding for CAII Hits

Carbonic anhydrase II (CAII) is a metalloenzyme responsible for the interconversion of carbon dioxide and bicarbonate for which noncovalent inhibitors have been developed as treatments for glaucoma, oedema, and cancer.^31,32^ CAII inhibitors are typically based on aromatic sulfonamide pharmacophores, which interact with the Zn^2+^ ion via the sulfonamide group.^33^

The HTC-D2B screen with CAII afforded four hits (**2b**–**e**) with modification yields of 66–75% (see Figure S5). Three of these hits contained an aromatic sulfonamide, consistent with known inhibitors, while one of the four (**2c**) was an aliphatic sulfonamide, of which there are few prior reports. These four compounds were the only primary sulfonamides in the library, and the remainder of the library gave an average modification yield of <1%, indicating negligible nonspecific modification and high specificity of the hit fragment interactions.

**Figure 3:**
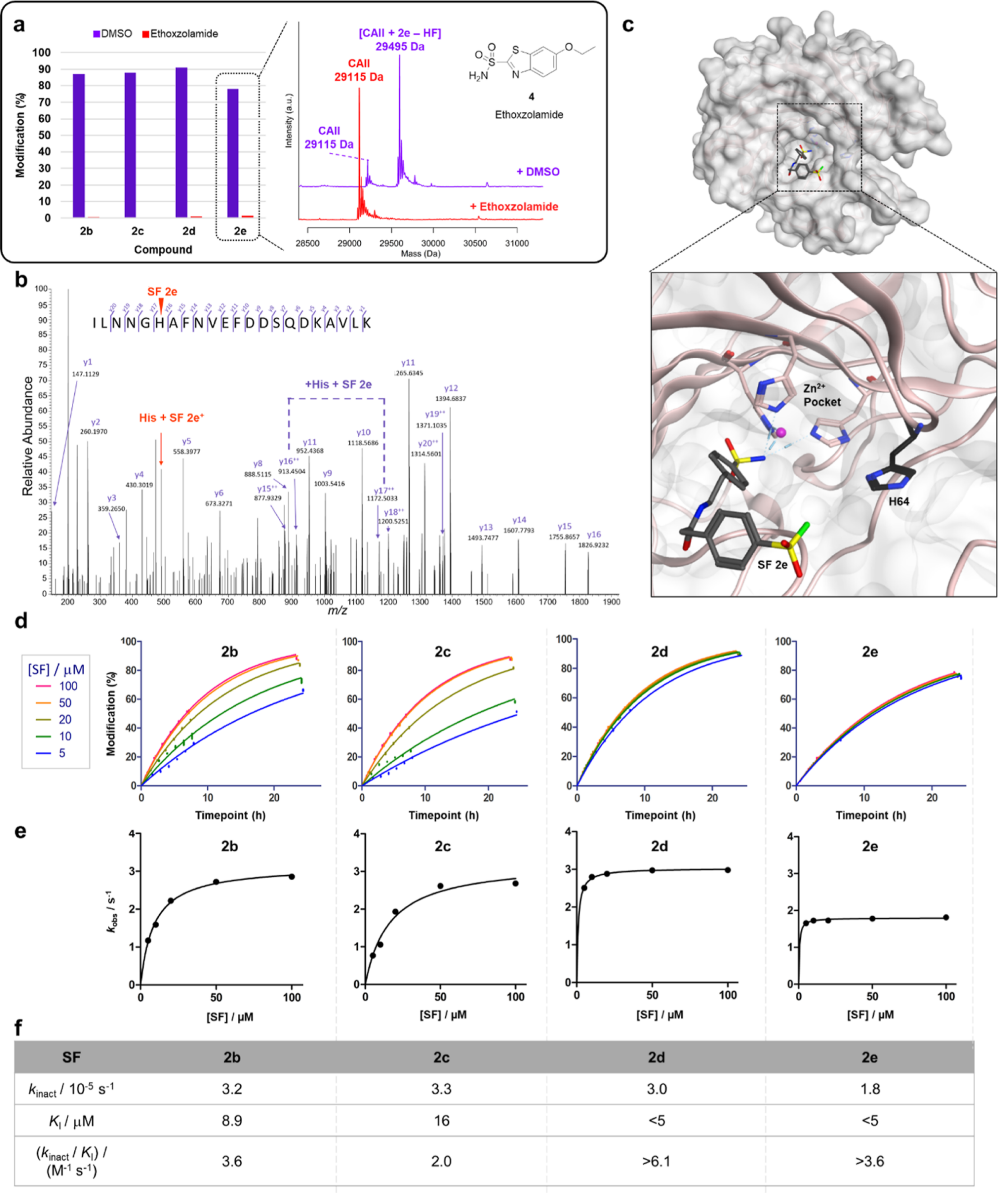
Determination of the site(s) of modification, rationalization with docking, and kinetic analyses for the measure of respective covalent modification efficiencies of CAII hits. (a) Modifications observed in the displacement studies and exemplar spectra observed following the displacement SF **2e** by ethoxzolamide. Final concentrations: 0.5 μM protein; 100 μM SF; and 50 μM ethoxzolamide. (b) Exemplar MS/MS spectrum of peptide _59_ILNNGH*AFNVEFDDSQDKAVLK_80_ modified by SF **2e** confirming His64 as the site of covalent modification. (c) X-ray crystal structure of CAII (PDB: 3CAJ) and virtual docking showing SF **2e** in the CAII pocket, with sulfonamide bound to the active site Zn^2+^ cofactor and sulfonyl fluoride group proximal to the His64 residue. (d) Time courses (various concentrations plotted against time and fitted to a single exponential function to determine *k*_obs_) showing concentration-dependent modification of SFs **2b**–**e** with CAII. Screening conditions: [protein] = 0.5 μM, [SF] = 100, 50, 20, 10, and 5 μM, 0–24 h, 20 °C. (e) *k*_obs_ measurements plotted against the measured concentrations of SFs **2b**–**e** to determine *k*_inact_ and *K*_I_. (f) Table displaying *k*_inact_, *K*_I_, and hence *k*_inact_/*K*_I_—a parameter to describe the overall modification efficiencies of SFs **2b**–**e**.

The site of binding was investigated by displacement studies.^34^ For this, we used the CAII inhibitor ethoxzolamide (**4**) (*K*_i_ = 8 nM), which is known to bind within the Zn^2+^ pocket.^31^ The resynthesized and purified SFs (100 μM) were coincubated with either ethoxzolamide (50 μM) or DMSO as a control, with CAII (0.5 μM). Inspection of the resultant mass spectra after a 24 h incubation revealed that the presence of ethoxzolamide had abolished covalent modification for all hits **2b**–**e**, indicating binding to the same site (Figure 3a).

Tandem MS analyses were subsequently employed for identification of the amino acid residue(s) that was covalently modified. Samples of CAII modified by the resynthesized and purified SFs **2d** and **2e** were digested using trypsin and analyzed by LC–MS/MS. This identified peptides _59_ILNNGH*AFNVEFDDSQDKAVLK_80_ and _59_ILNNGH*AFNVEFDDSQDK_76_ as the major site(s) of modification on His64 for both **2d** (366 Da) (see Figure S6) and **2e** (380 Da) (Figure 3b), respectively. Further to this, minor modifications were observed on the N-terminal peptide _1_MSHHWGYGK_9_ at His3 (**2d** and **2e**) and Tyr7 (**2d** only).

To rationalize these observations, virtual dockings were carried out on SFs **2d** and **2e**, based on the reported binding mode of ethoxzolamide (PDB: 3CAJ). For **2e**, the docking indicated that the sulfonamide was bound to the Zn^2+^ cofactor, and the His64 residue was proximal to the SF group, just 7 Å away, and appears well poised for reaction (Figures 3c and S7).^35^ Similarly, for **2d**, the Zn^2+^–sulfonamide interaction was observed, and the SF group was in the vicinity of His64, as well as residues His3 and Tyr7 which were also found to carry minor modifications (see Figure S8).

### Kinetic Analyses of CAII Hits

The kinetics of binding were investigated by incubation of the resynthesized and purified SFs **2b**–**e** with CAII at a range of concentrations and analysis by intact protein LC–MS over a 24 h period (see Figure S9). The concentration–response was analyzed based on a two-step model of reversible ligand (L) binding and subsequent irreversible covalent modification of the protein (P) (P + L ⇌ P·L → PL) (see Figure S10).^24^ Time courses were fit to a single exponential function to give a *k*_obs_ for each concentration (Figure 3d). These were then fitted using a Michaelis–Menten model to determine *k*_inact_ (corresponding to the rate of covalent-bond formation) and *K*_I_ (corresponding to the recognition of the ligand for the protein pocket) (Figure 3e,f). All fragments were found to have strong reversible affinity (*K*_I_), and these were below the minimum concentration screened for **2d** and **2e** (<5 μM). This is consistent with the strong binding of aryl sulfonamides to the CAII Zn^2+^ binding pocket.^33^ The SFs exhibited unexpectedly slow rates of covalent modification, with 50% modification achieved between 6.5 and 10.5 h for all 100 μM conditions. The slow rate of the reaction may be attributed to a suboptimal trajectory of the SF group toward the His64 residue in the reversible bound conformation and/or low nucleophilicity of the residue.^14^

An advantage of the HTC-D2B approach taken with this screening strategy is the opportunity to rapidly explore iterative libraries of compounds which are structurally similar to the original hits identified. Such iteration-based screens allow for faster design-make-test cycles in contrast to traditional methods in early-stage drug discovery, leading to the more efficient identification of potent and selective probes.^29^ To explore whether any alternative sulfonamide-containing fragments would give high modification yields at a faster rate, a new library was designed. For this, 96 amine-functionalized fragment analogues of hits **2b**–**e** were selected, coupled to OSu ester **2a**, and the resultant library was incubated with CAII for 1 h prior to analysis by intact protein LC–MS. Inspection of the resultant mass spectra after this short incubation revealed many fragment hits that exhibited significantly higher modification yields (>70%, see Figure S11) when compared with the original hits (<15% at 1 h, see Figure 3d). This improvement in rate of modification is particularly notable given that the second-generation HTC-D2B library would be unlikely to have 100% purity as compared to the purified hits from the first-generation library. The improvement in potency demonstrates the utility of the HTC-D2B approach as a means to rapidly optimize kinetic parameters, with respect to both reversible recognition (*K*_i_) and also the electrophile trajectory in determining *k*_inact_.

### Assessment of Target Engagement in Cells for CAII Hits

**Figure 4:**
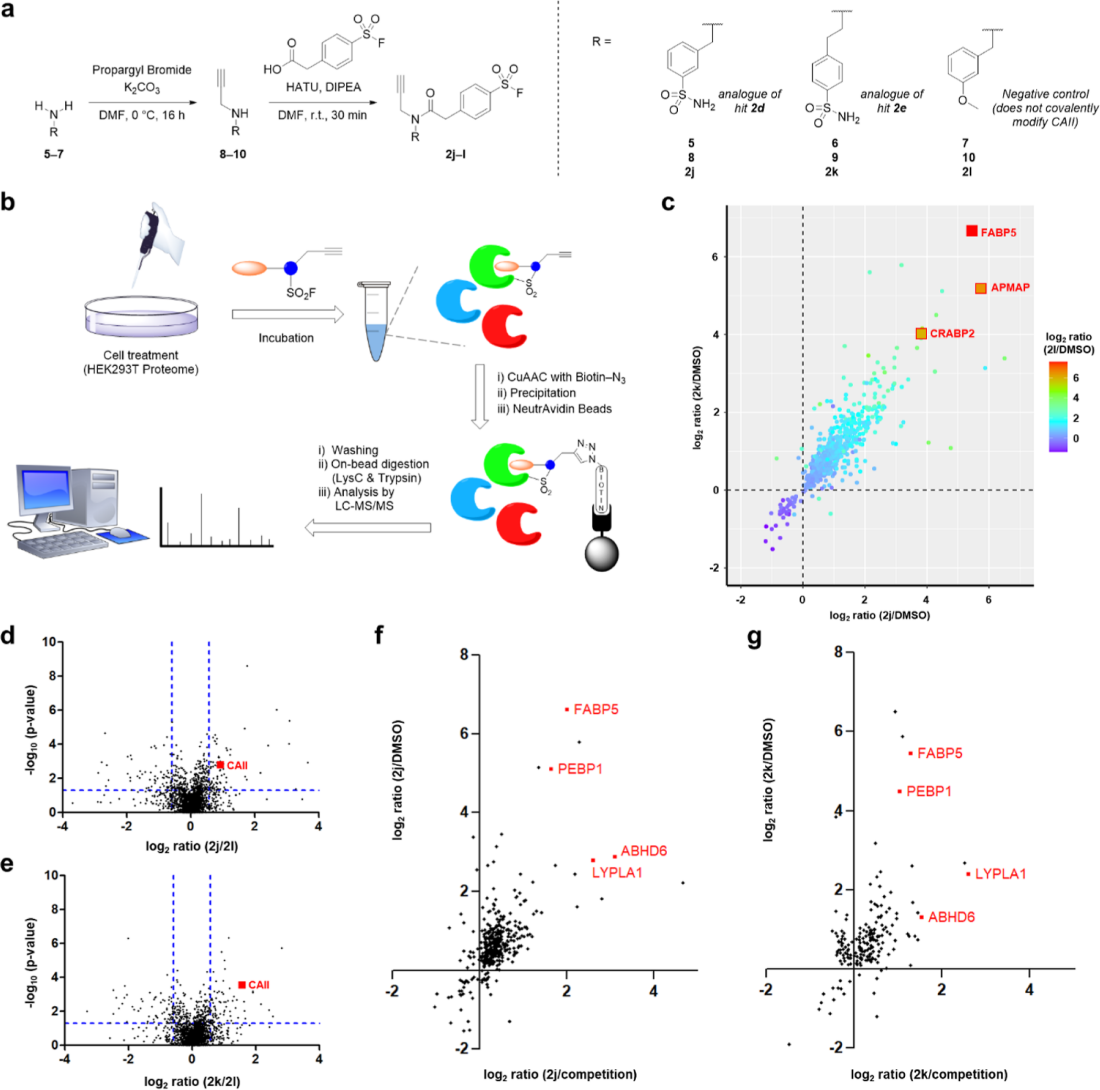
Assessing target engagement in cells with chemoproteomics. (a) Scheme depicting the synthesis of probes **2j** and **2k** (alkyne-functionalized analogues of CAII hits **2e** and **2d**, respectively) and negative control **2l**. (b) Schematic representation of MS-based proteomic workflow used to assess target engagement in cells. (c) Plot of **2j** and **2k** log_2_ ratios colored by **2l** log_2_ ratio, highlighting commonly enriched proteins. (d) Volcano plot highlighting CAII enrichment by SF **2j**, plotted as a log_2_ ratio compared to negative control **2l**. Blue dashed lines correspond to thresholds: log_2_ ratio ≥0.58; *p*-value ≤0.05. (e) Volcano plot highlighting CAII enrichment by SF **2k**, plotted as a log_2_ ratio compared to negative control **2l**. Blue dashed lines correspond to thresholds: log_2_ ratio ≥0.58; *p*-value ≤0.05. (f) Log_2_ representation of fold difference between probe **2j**/competition and **2j**/DMSO, highlighting commonly enriched proteins. (g) Log_2_ representation of fold difference between probe **2k**/competition and **2k**/DMSO, highlighting commonly enriched proteins.

Hits from the CAII screen were progressed to chemoproteomic profiling to determine cellular target engagement and to measure their off-target profiles. SFs **2d** and **2e** were functionalized with an alkyne handle at the linking amide group to give probes **2j** and **2k**. A structurally similar negative control was also designed by substitution of the sulfonamide for a methoxy group, **2l** (Figure 4a). The three probes were first incubated with purified CAII, which confirmed that SFs **2j** and **2k** covalently modified CAII following addition of the alkyne group and that the methoxy group-containing negative control **2l** did not. The proteins engaged by these three probes were subsequently studied in HEK293T cells.

HEK293T cells were treated for 1 h with probes **2j**, **2k**, or **2l** (10 μM) or the DMSO vehicle. Competition-based experiments were also conducted where cells were pretreated with parent hits **2d** (40 μM) or **2e** (40 μM) for 1 h, before the addition of alkyne-containing probes **2j** (10 μM) or **2k** (10 μM), respectively. Following incubation, treated cells were lysed and conjugated with biotin-azide by Cu-click, and biotinylated proteins were enriched using NeutrAvidin beads. Enriched proteins were digested with LysC and trypsin, prior to analysis by LC–MS/MS (Figure 4b).

All three probes (**2j**–**l**) were found to enrich multiple proteins by comparison to the DMSO control (523, 501, and 447, respectively, log_2_ ratio ≥0.58, *p*-value ≤0.05, and #unique peptides ≥2), highlighting the promiscuity of the reactive fragments in this environment. A good correlation was observed between all three probes and the proteins enriched, indicating that most of the enrichment was driven by the SF moiety, rather than the fragment portion of the probes (Figure 4c). The most significantly enriched proteins included FABP5, APMAP, and CRABP2. Previous reports identified that FABP5 and CRABP2 were targeted by an arylfluorosulfate probe by reaction at a tyrosine residue in each of these proteins.^36^ While some enrichment of CAII was observed for **2j** and **2k**, this was poorly resolved among the many other enriched proteins. To further investigate CAII engagement of the sulfonamide-containing active probes (**2j** and **2k**) in live cells, we compared the enriched proteins to those enriched by the methoxy-containing negative control (**2l**). This highlighted CAII as one of the few differentially enriched proteins, suggesting that the aryl sulfonamide fragment hits were driving cellular engagement of CAII (Figure 4d,e).

Interestingly, competition experiments with **2d** and **2e** showed relatively few significantly competed proteins, suggesting that many of the interactions involved substoichiometric binding. This was true for CAII, where neither probes **2j** nor **2k** showed competition with parents **2d** or **2e**, respectively. This is consistent with the slow rate of covalent modification of CAII by **2d** and **2e** in the biochemical kinetic analyses, which suggested that the protein would only be partially modified after a 1 h incubation. Together, these results indicate that while the aryl sulfonamide fragment hits did engage CAII in cells, it was with low stoichiometry and poor selectivity over many additional off-targets.

Further investigation of the off-targets revealed that some proteins were competed in the presence of a parent, indicating high levels of engagement; these included FABP5, PEBP1, ABHD6, and LYPLA1 (Figure 4f,g).^37^ ABHD6 and LYPLA1 catalyze the hydrolysis of esters and thioesters, respectively, and have nucleophilic catalytic residues which are likely to react with the SF moiety.^38,39^ Similarly, PEBP1 is a phospholipid binder and is the prototype of a novel family of serine protease inhibitors.^40^ More specifically, each of these proteins bind molecules containing hydrophobic alkyl chains, consistent with previous reports involving SF-containing probes targeting FABP5 in cells.^36^ An alternative explanation for these observations is the presence of a consistent benzene SF group on each of the probes, which may be preferentially bound by these proteins. The high levels of occupancy at these targets suggest that they may be amenable to the development of potent SF-based covalent inhibitors.

### BCL6 Follow-Up Studies: Site(s) of Binding and Hit Expansion

**Figure 5:**
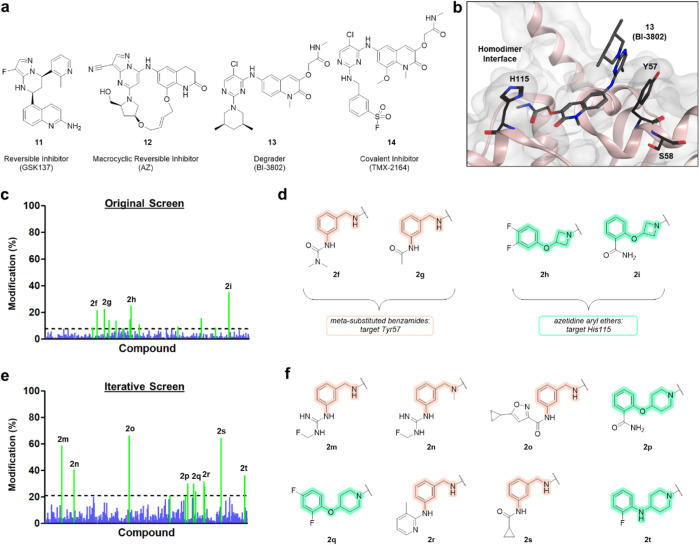
BCL6 hits validated and expanded upon with iterative screen. (a) Structures of reported BCL6 binders **11**–**14**. (b) Crystal structure of compound **13** (BI-3802) in complex with the BCL6 BTB/POZ domain, highlighting proximal nucleophilic amino acid residues (PDB: 5MW2). (c) Summary of original screen against BCL6 with reactive moiety **2**. The dashed line shows hit threshold at 10%; hits colored green and nonhits colored blue. (d) Structures of hits **2f**–**i** involving two chemotypes: meta-substituted benzamide fragments (highlighted peach) and azetidinyl/piperidinyl fragments (highlighted turquoise). (e) Summary of iterative screen against BCL6 with the library of hit fragment analogues. Conditions of original screen: [protein] = 1 μM, [SF] = 20 μM, 24 h, 4 °C. Conditions of iterative screen: [protein] = 1 μM, [SF] = 50 μM, 24 h, 4 °C. The dashed line shows hit threshold at 21%; hits colored green and nonhits colored blue. (f) Structures of hits **2m**–**t** with two consistent chemotypes.

The transcription factor B-cell lymphoma 6 (BCL6) has been identified as a driver of oncogenesis in lymphoid malignancies. BCL6 is implicated in several protein–protein interactions with corepressors, and disruption of these interactions is currently being investigated as a strategy for cancer treatment.^41^ A range of small molecules that target BCL6 have been reported, including reversible inhibitors such as compound **11** (GSK137) and macrocyclic compound **12**.^41,42^ Compounds with alternative mechanisms have also been reported, including degraders such as compound **13** (BI-3802) and more recently rationally designed covalent inhibitor **14** (TMX-2164) that targets Tyr57 (Figure 5a,b).^43,44^

The top hits from the SF screen with BCL6 represented two distinct chemotypes: meta-substituted benzamides (**2f** and **2g**) and azetidine aryl ethers (**2h** and **2i**). These were resynthesized and purified for follow-up studies. Tandem MS was used to identify the amino acid residue(s) responsible for covalent modification, and interestingly, the two different chemotypes were found to target two different residues (Figure 5c,d). The results showed that meta-substituted benzamides **2f** (373 Da) and **2g** (344 Da) modified Tyr57 on the peptide _47_TVLMACSGLFY*SIFTDQLKR_66_, and azetidine aryl ethers **2h** (365 Da) and **2i** (372 Da) modified His115 on the peptide _98_EGNIMAVMATAMYLQMEH*VVDTCR_121_ (see Figure S12). Tyr57 has previously been targeted by a BCL6 inhibitor (**14**, TMX-2164); however, the targeting of His115 is a novel modification; these residues are located on opposite sides of the binding site of previously reported BCL6 inhibitors (**11**–**14**) (see Figure 5b).

An iterative screen was subsequently carried out with the intention of expanding the pool of hit compounds and identifying BCL6 hits with higher covalent modification yields. For this, a new 352-membered library of SF-based reactive fragments was designed based on a similarity search of available amine-functionalized fragments using the top four original hits (**2f**–**i**). The library was generated by HTC, incubated with BCL6 in a D2B fashion, and subsequently analyzed by intact protein LC–MS. The extent of covalent modifications observed for this screen was markedly higher, with many further hits discovered (**2m**–**t**). This included five reactive fragments which gave covalent modification yields greater than the maximum modification yields observed in the original screen and three hits with over 50% modification (Figure 5e,f). The meta-substituted benzamides gave the higher modification yields, and several different groups in the meta position were well-tolerated. Other hits showed that different N-containing saturated heterocycles with alternative heteroatom links to aryl groups were also tolerated by the binding pocket.

### Structural and Biophysical Investigations into BCL6 Hits

**Figure 6:**
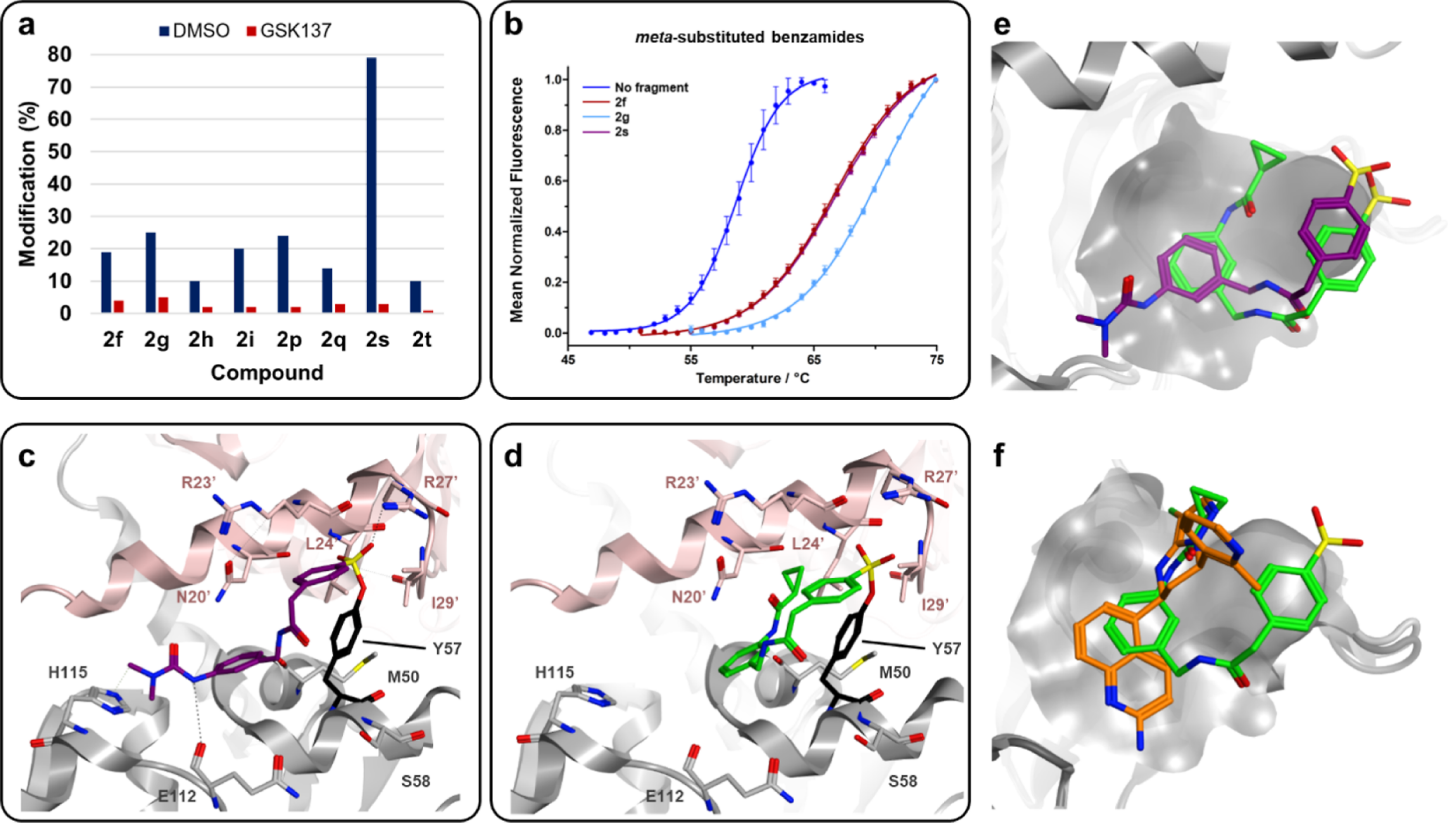
Investigations of BCL6 hits provide further structural insights into binding modes of hit SFs. (a) Incubation of GSK137 with BCL6 abolishes covalent modification by meta-substituted benzamide and azetidinyl/piperidinyl SF fragments. (b) Covalent modification by the meta-substituted benzamide hits increases the stability of BCL6 in the conventional DSF assay. Each point represents an average of three replicates. (c) Cocrystal structure of SF **2f** with the BCL6 BTB/POZ dimer. One monomer is shown in gray; one monomer is shown in pink. Compound **2f** is shown in stick representation with carbon atoms colored purple. (d) Cocrystal structure of SF **2s** with the BCL6 BTB/POZ dimer. One monomer is shown in gray; one monomer is shown in pink. Compound **2s** is shown in stick representation with carbon atoms colored green. (e) Overlaid crystal structures of **2f** and **2s** showing the aryl group of respective benzamides sharing a common binding surface. Tyr57 residue is omitted for clarity. Compounds **2f** and **2s** are shown in stick representation, with carbon atoms colored purple and green, respectively. (f) Overlaid crystal structures of GSK137 (PDB: 7BDE) and **2s** which have overlapping binding sites in the final crystallographic state. Tyr57 is omitted for clarity. Compounds GSK137 and **2s** are shown in stick representation, with carbon atoms colored orange and green, respectively.

With additional hits identified from the iterative screen, we sought to obtain further structural and biophysical information to better understand how these reactive fragments bind to BCL6. A total of eight SF hits were resynthesized and purified: the four from the original screen (**2f**–**i**) and four from the iterative screen (**2p**, **2q**, **2s**, and **2t**). To confirm that these eight hits targeted the same binding site as previously reported binders, GSK137 (biochemical pIC_50_ = 8) was used as a known inhibitor for a displacement experiment.^42^ The SFs (100 μM) were incubated for 24 h with BCL6 in the presence of GSK137 (100 μM) or DMSO as a control, and the samples were analyzed by intact protein LC–MS. The resultant mass spectra showed that covalent modification was attenuated by 78–96% for all eight SFs, indicating that all hits were competing for the same site (Figure 6a).^42^

We subsequently analyzed protein-fragment interactions using differential scanning fluorimetry (DSF) to explore the impact of these binding events on protein stability.^45^ The azetidinyl/piperidinyl fragments exhibited negligible thermal shifts (see Figure S13b,c); however, the meta-substituted benzamide complexes were highly thermally stabilized (Δ*T*_m_ ∼ 7 °C) relative to unmodified BCL6, which implied the presence of intermolecular interactions from ligand binding (Figure 6b). This level of stabilization was comparable to that observed for the potent, noncovalent inhibitor GSK137 (Δ*T*_m_ ∼ 12 °C, see Figure S13d).^42^ Kinetic analyses of the four hits from the original screen (**2f**–**i**) revealed that for the meta-substituted benzamides, the reversible affinities of these fragments were relatively weak, with *K*_I_ = 56 and 80 μM for **2f** and **2g**, respectively (see Figure S14). Therefore, it was interesting to observe a thermal shift similar to an inhibitor with biochemical pIC50 = 8; this illustrates the impact of covalent modification on protein–ligand binding.

Crystal structures of SFs **2f** and **2s** within BCL6 were generated to further investigate the binding mode of the fragments; this used a previously published protocol.^42^ Cocrystal structures of **2f** and **2s** with BCL6 (solved to 1.6 and 1.8 Å resolution, respectively) revealed Tyr57 covalently conjugated to the fragments via a sulfonate ester, consistent with tandem MS studies for SF **2f** (Figure 6c,d). The residue Arg27 was observed to be in close proximity to the sulfonyl groups of **2f** and **2s** which may have catalyzed the covalent modification of Tyr57 via hydrogen-bonding interactions. While the meta-substitutions of the benzamides showed opposite trajectories, a common binding surface of the benzamide aryl ring was observed, suggesting that a trisubstituted aryl group might be tolerated here (Figure 6e). The twisted conformation of **2s** could also represent a premodified state of the protein–SF complex, which may have become disrupted for **2f** upon covalent modification with Tyr57 to afford the extended conformation observed. The structures of GSK137 and **2s** were overlaid, revealing that the two binding sites had significant overlap in the final crystallographic state (Figure 6f). This suggested that **2s** may have similar inhibitory action to GSK137 on the BCL6 BTB/POZ domain.^42^

## Conclusions

Covalent inhibitors are of high interest in chemical biology and drug discovery for expansion of the liganded proteome and for liganding challenging therapeutic targets.^46^ The majority of efforts to develop covalent inhibitors have focused on the targeting of cysteine residues in the vicinity of protein pockets. However, to tackle the increasing number of genetically validated targets that drug discovery teams are presented with, approaches to covalent ligand discovery without reliance on cysteine are highly sought after. SFs offer the opportunity to target a broad repertoire of nucleophilic amino acids and hence are effective reactive moieties to consider for “beyond-cysteine” covalent inhibitors. To date, SF-containing chemical probes have been rationally designed based on structure-guided approaches; this typically entails the installation of an SF group onto a potent reversible scaffold.^47^ Here, we have demonstrated that sulfonyl fluoride-reactive fragments offer an expedient approach to identify novel “beyond-cysteine” covalent ligands.

Screening a modest library of 352 fragments afforded hits for two of the three proteins assessed, which were found to interact with functionally relevant pockets. An advantage of screening with reactive fragments over classical reversible fragments is the facile identification of the site(s) of covalent modification using tandem MS. Hits identified here were found to modify tyrosine and histidine residues. The observation of histidine modification under the denaturing MS workflows was perhaps surprising since previous reports have suggested that histidine adducts of SFs can be unstable. The multiple examples of histidine modification provide evidence that this is not always the case and that SFs are suitable electrophiles for targeting histidine residues.^10,19,48,49^

Kinetic analyses of the CAII hits provided a measure of covalent modification efficiencies and parameters *k*_inact_ and *K*_I_. Interestingly, the CAII original hits exhibited good noncovalent affinity (*K*_I_) but slow rates of covalent modification (*k*_inact_). This suggested either low nucleophilicity of the histidine residue or suboptimal geometry for covalent modification. An HTC-D2B screen of analogues close to the original hits identified fragments that gave fast rates of modification (>70% covalent modification in <1 h), highlighting the potential roles of electrophile position and reactivity in the design of covalent inhibitors and the utility of the HTC-D2B approach as a means to optimize both *k*_inact_ and *K*_I_.

The chemoproteomic analyses of the CAII hits revealed that they engaged CAII in cells, however, at low stoichiometry, likely due to the low *k*_inact_ rates for these hits. The two CAII hits and the negative control were also found to interact with many additional proteins in cells (>100), many of which were common to all three SF compounds. This highlighted that specificity is likely to be a key challenge to overcome when developing SF-based inhibitors. Previous work in our group has demonstrated that the reactivity of the SF electrophile is highly tunable.^14^ Therefore, it is anticipated that selective target engagement in cells would be achievable through reduction of the intrinsic reactivity of the SF while optimizing *k*_inact_ and *K*_I_ for the target. The HTC-D2B approach described here provides a useful strategy for performing this optimization.

The SF fragment screen also identified novel hits for BCL6, a therapeutic target under investigation for conditions such as blood, breast, and lung cancers.^42^ Interestingly, the screen afforded two hit series that were found to covalently modify residues on opposite sides of the same pocket, one to a tyrosine and one to a histidine, highlighting the versatility of the approach in targeting nucleophilic amino acid residues within a binding pocket. In this case, the pool of hit compounds was rapidly expanded upon by an iterative screen, and the hits represented two novel chemotypes for BCL6. The attenuation of covalent modification when incubated in the presence of GSK137, a published BCL6 inhibitor, implied that the fragments target a known small-molecule binding surface.^42^ Cocrystal structures of BCL6 with hit SFs were obtained, which confirmed that the binding conformations overlapped with that of GSK137, positing the fragment as a covalent probe to aid the discovery of novel inhibitors of the BCL6 BTB/POZ domain.

Collectively, these results demonstrate that SF-reactive fragment screening offers an efficient approach for the discovery of “beyond-cysteine” covalent ligands. The HTC-D2B approach enables rapid iterative design-make-test cycles to drive toward more potent and selective chemical tools. This HTC-D2B workflow facilitates the exploration of alternative SF groups as the reactive moiety, which can be exploited to reduce the intrinsic reactivity, and thus promiscuity, of the fragments while simultaneously optimizing *k*_inact_ and *K*_I_ for the target of interest. This will support the development of covalent chemical probes to expand the liganded proteome and ultimately further our understanding of disease biology.

## Methods

Full experimental details including synthesis and data processing are provided in the Supporting Information.

### HTC-D2B Protocol

To a 384-well plate containing 352 amine-functionalized fragments (10 mM) in DMSO (5 μL per well) was added a stock solution of OSu **2a** (10 mM) and NEM (30 mM) in DMSO (5 μL per well). The plate was sealed, centrifuged (1 min, 1000 rpm), and allowed to sit at room temperature for 1 h. After the reaction (assumed product concentration: 5 mM), the library of SFs to a Greiner 384 was echo dispensed into a low volume plate. Purified protein (see the Supporting Information for concentration) was subsequently added across the plate. The plate was sealed, centrifuged, incubated for 24 h, and then analyzed by intact protein LC–MS.

### Identification of the Site of Modification

The (resynthesized and purified) SF hits (**2d** and **2e**, 10 mM) were plated into a 384-well plate. Purified CAII was subsequently added across the plate. Final concentrations: 2 μM protein; 50 μM SF. The plate was sealed, centrifuged, and incubated at 20 °C for 24 h, and 15 μL aliquots were subsequently removed and analyzed by intact protein LC–MS. The remaining samples (1 μg) were separated by sodium dodecyl sulfate-polyacrylamide gel electrophoresis to remove excess unbound compounds. Gels were stained with colloidal Comassie InstantBlue, and bands corresponding to CAII were excised, reduced with TCEP (10 mM, 65 °C, 30 min), and alkylated with iodoacetamide (10 mM, rt, 30 min, dark). Samples were digested with trypsin (1:10 E/S, 37 °C, 16 h) in ammonium bicarbonate (100 mM). After removal of the supernatant, peptides were extracted using acetonitrile. Combined supernatants were centrifuged and acidified prior to injection into the LC–MS/MS system. Tandem MS spectra were searched for peptide matches against the sequence for CAII. Raw files were searched using trypsin as the enzyme. Masses corresponding to [SF – HF] were allowed as variable modification(s) on cysteine, histidine, lysine, tyrosine, serine, and threonine as well as the protein N-terminus. MS/MS spectra were manually validated and annotated.

### Kinetic Analyses

The (resynthesized and purified) SF hits (**2b**–**e**, 10 mM) were plated into seven 384-well plates with DMSO to make up identical plates with final concentrations of 100, 50, 20, 10, or 5 μM after adding 15 μL of protein stock solution per well. The percentage of DMSO was kept constant. Purified CAII was subsequently added across all plates. Final concentrations were 0.5 μM protein; 100, 50, 20, 10, or 5 μM SF. The plates were sealed and centrifuged, and the first six plates were immediately queued for analysis by intact protein LC–MS at a temperature of 20 °C with the final plate paused for a 24 h timepoint.

## Supporting Information Available

Full details of materials and methods, including compound synthesis and characterization data, full structures, summary of LC–MS analyses, binned properties, summary of screen against CAII, MS/MS spectrum, crystalography, exemplar time course, two-step binding mechanism, summary of iterative screen, DSF traces, kinetic analyses, and details of biological studies (PDF)
