# Supplementary material for: Efficient Ligand Discovery Using Sulfur(VI) Fluoride Reactive Fragments
Source: ACS Chem Biol. 2023 Apr 21;18(9):1926–37. doi: 10.1021/acschembio.3c00034 (PMC10510102; doi:10.1021/acschembio.3c00034)
Supplement: Supplementary file 1 — cb3c00034_si_001.pdf [file cb3c00034_si_001.pdf]

SUPPORTING INFORMATION

Efficient Ligand Discovery Using Sulfur(VI) Fluoride Reactive Fragments

Arron Aatkar[†‡], Aini Vuorinen[†ȡ], Oliver E. Longfield[†‡], Katharine Gilbert[†‡], Rachel Peltier-Heap[ǁ], Craig D. Wagner[ǁ], Francesca Zappacosta[ǁ], Katrin Rittinger[ȡ], Chun-wa Chung[†], David House[†ȡ], Nicholas C. O. Tomkinson[‡], Jacob T. Bush[†ȡ*].

[†]GSK, Gunnels Wood Road, Stevenage, Hertfordshire, SG1 2NY, UK. [‡]Department of Pure and Applied Chemistry, University of Strathclyde, 295 Cathedral Street, Glasgow, G1 1XL, UK. [ȡ]The Francis Crick Institute, London, NW1 1AT, UK. [ǁ]GSK, South Collegeville Road, Collegeville, PA 19426, USA.

*Corresponding authors: nicholas.tomkinson@strath.ac.uk, jacob.x.bush@gsk.com

Contents

1. **Supplemental Figures** ... 3
2. **General Experimentation** ... 9
   2.1. Solvents; reagents; consumables; materials ... 9
   2.2. Liquid chromatography-mass spectrometry (LC-MS) for small molecules ... 9
   2.3. Flash column chromatography (FCC) ... 10
   2.4. Mass-directed automated preparative HPLC (MDAP) ... 10
   2.5. Nuclear magnetic resonance (NMR) spectroscopy ... 10
   2.6. Infrared (IR) spectroscopy ... 10
   2.7. Centrifuge ... 11
   2.8. Intact protein LC-MS ... 11
3. **High-throughput chemistry (HTC) and direct-to-biology (D2B) protocols** ... 12
   3.1. HTC protocol trial ... 12
   3.2. Selection of 352 amine functionalized fragments ... 12
   3.3. HTC-D2B protocol ... 13
4. **CAII – site(s) of binding studies** ... 14
   4.1. Displacement study with ethoxzolamide ... 14
   4.2. Identification of the site of covalent modification by tandem MS (CAII) ... 14
   4.3. Virtual docking of hit compounds ... 14
5. **CAII – kinetic analyzes** ... 16
   5.1. Protocol for kinetic analyzes (CAII) ... 16
   5.2. Protocol for iterative screen against CAII ... 16
6. **CAII – chemoproteomics** ... 17
   6.1. Single shot screen of chemoproteomic probes against CAII ... 17
   6.2. Chemoproteomics workflow ... 17
   6.3. LC-MS/MS analysis ... 18
   6.4. Data analysis ... 18
   6.5. Data availability ... 18
7. **BCL6 – site of binding studies and iterative screen** ... 19
   7.1. Identification of the site(s) of covalent modification by tandem MS (BCL6) ... 19
   7.2. Protocol for iterative screen against BCL6 ... 19
8. **BCL6 – Further investigations into hits by structural and biophysical studies** ... 21
   8.1. Displacement study with GSK137 ... 21
   8.2. Conventional differential scanning fluorimetry (DSF) ... 21
   8.3. Protocol for kinetic analyzes (BCL6) ... 22
   8.4. Protocol for crystallography ... 22
9. **Compounds** ... 25
10. **References** ... 32

1. Supplemental Figures

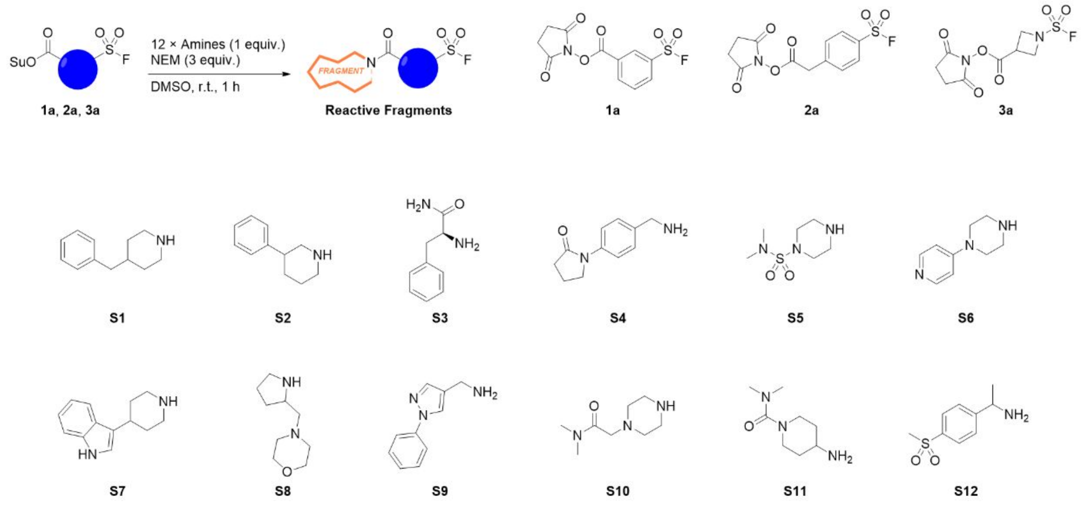

Figure S1. Full structures of reactive moieties and amine-functionalized fragments used to trial the HTC conditions.

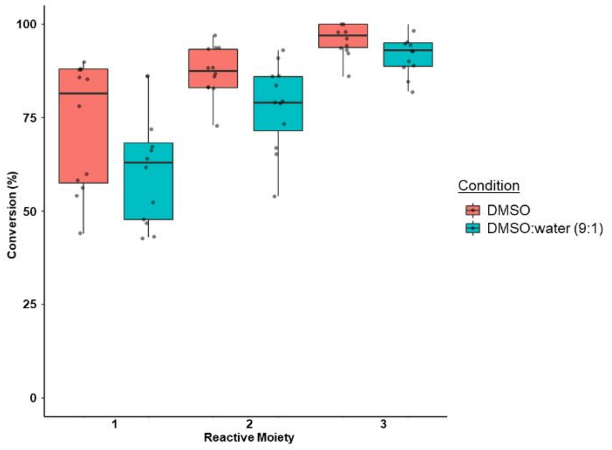

Figure S2. Summary of LC-MS analyzes across 12 wells (reactions with amines **S1–S12**) used to trial the high-throughput chemistry protocol. DMSO condition shown in red; DMSO:water (9:1) condition shown in blue.

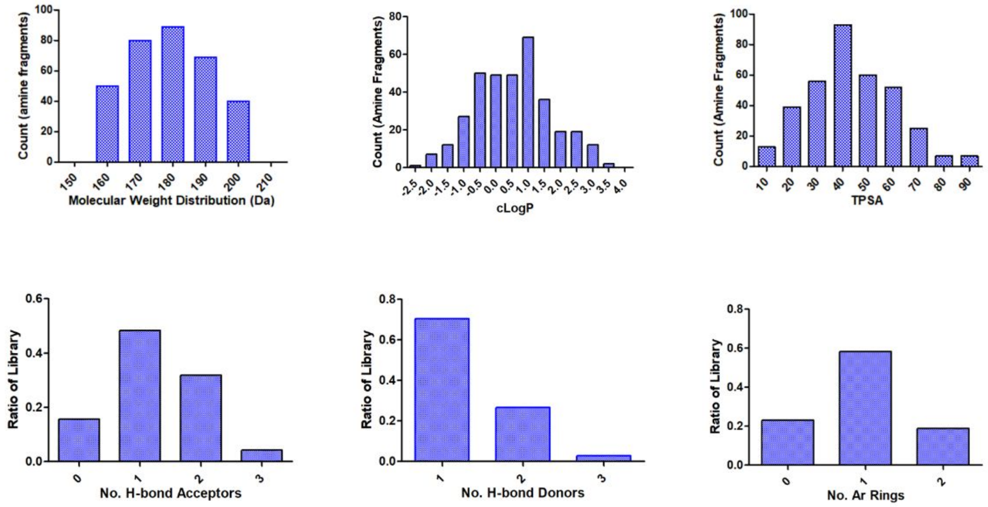

Figure S3. Binned properties of the original 352-membered amine-functionalized fragment library.

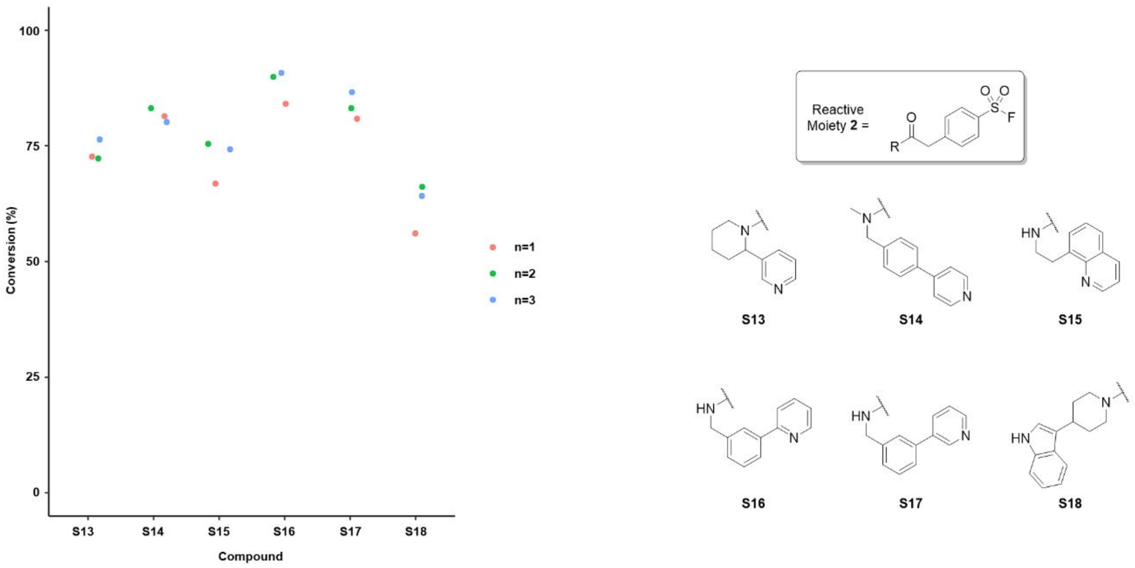

Figure S4. LC-MS analysis of six wells selected at random to assess the conversion of the HTC protocol in a plate-based format.

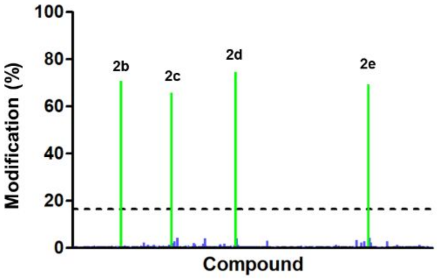

Figure S5. Summary of screen against CAII with reactive moiety **2**. Dashed line shows hit threshold at 17% (mean + 2 SDs); hits colored green; non-hits colored blue.

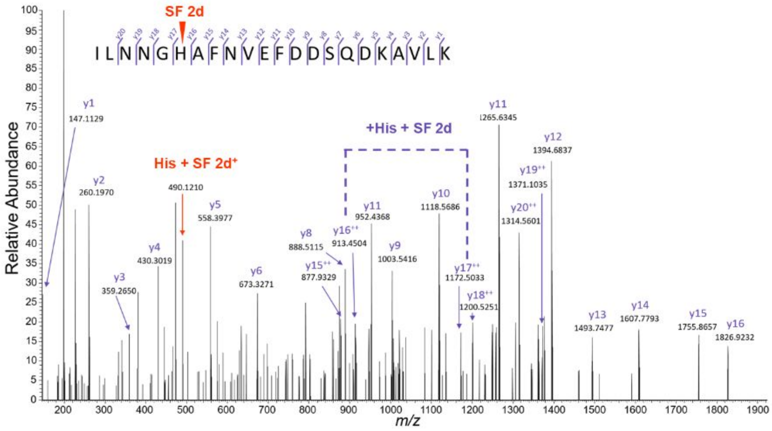

Figure S6. MS/MS spectrum of peptide $_{59}$ILNNGH*AFNVEFDDSQDKAVLK$_{80}$ modified by SF **2d** confirming His64 as the site of covalent modification.

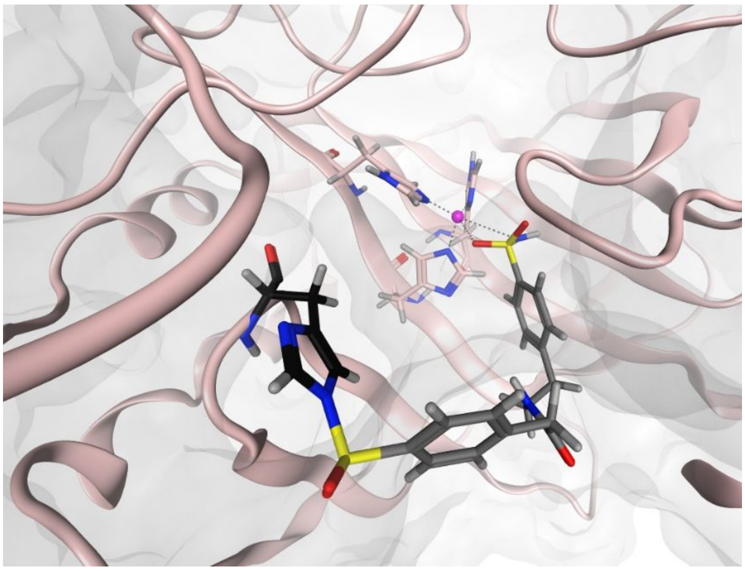

Figure S7. X-ray crystal structure of CAII (PDB: 3CAJ), and virtual docking showing SF **2e** covalently bound to His64 in the CAII pocket.

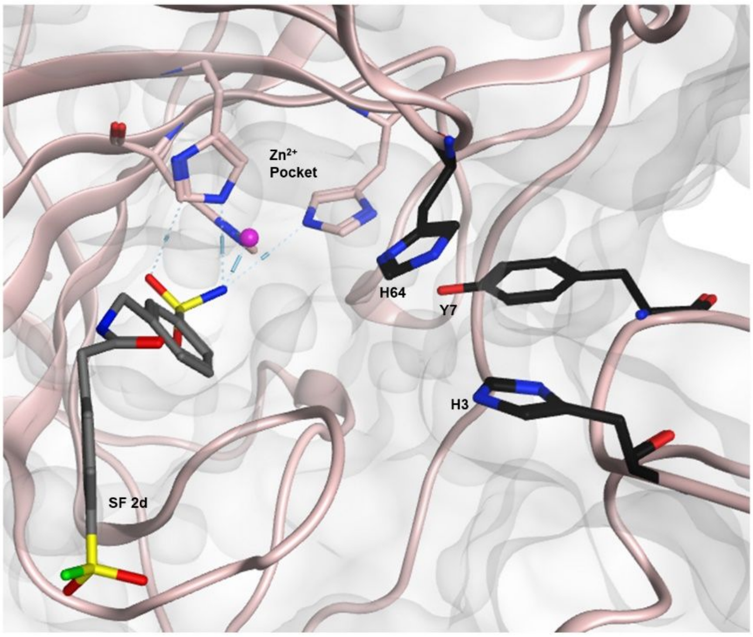

Figure S8. X-ray crystal structure of CAII (PDB: 3CAJ), and virtual docking showing SF **2d** in the CAII pocket, highlighting residues His64, His3, and Tyr7.

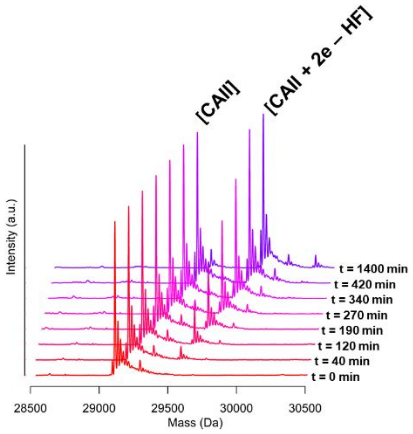

Figure S9. Exemplar time course (intact protein LC-MS) showing time-dependent modification of CAII with **2e** at 100 μM.

$$[P] + [L] \underset{}{\overset{K_I}{\rightleftharpoons}} [P \cdot L] \xrightarrow{k_{inact}} [P\text{–}L]$$

$$\text{Modification (\%)} = 100(1 - \exp(-k_{obs}t)) \qquad (1)$$

$$k_{obs} = \frac{k_{inact}[L]}{K_I + [L]} \qquad (2)$$

Figure S10. Two-step binding mechanism for irreversible electrophilic protein modifiers and equations (1) and (2) for the calculation of kinetic parameters.

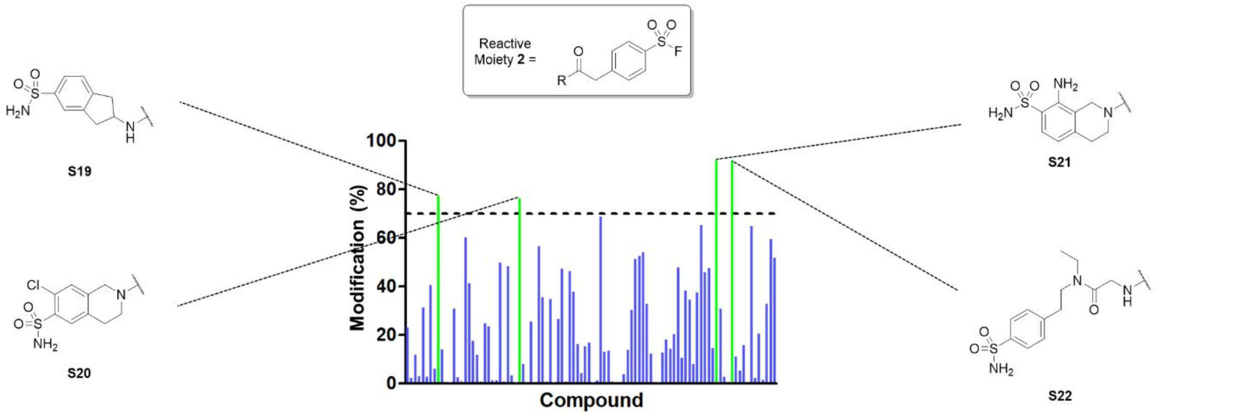

Figure S11. Summary of iterative screen against CAII with reactive moiety **2** and library of sulfonamide-containing fragments. Dashed line shows hit threshold at 70% (mean + 2 SDs); hits colored green; non-hits colored blue.

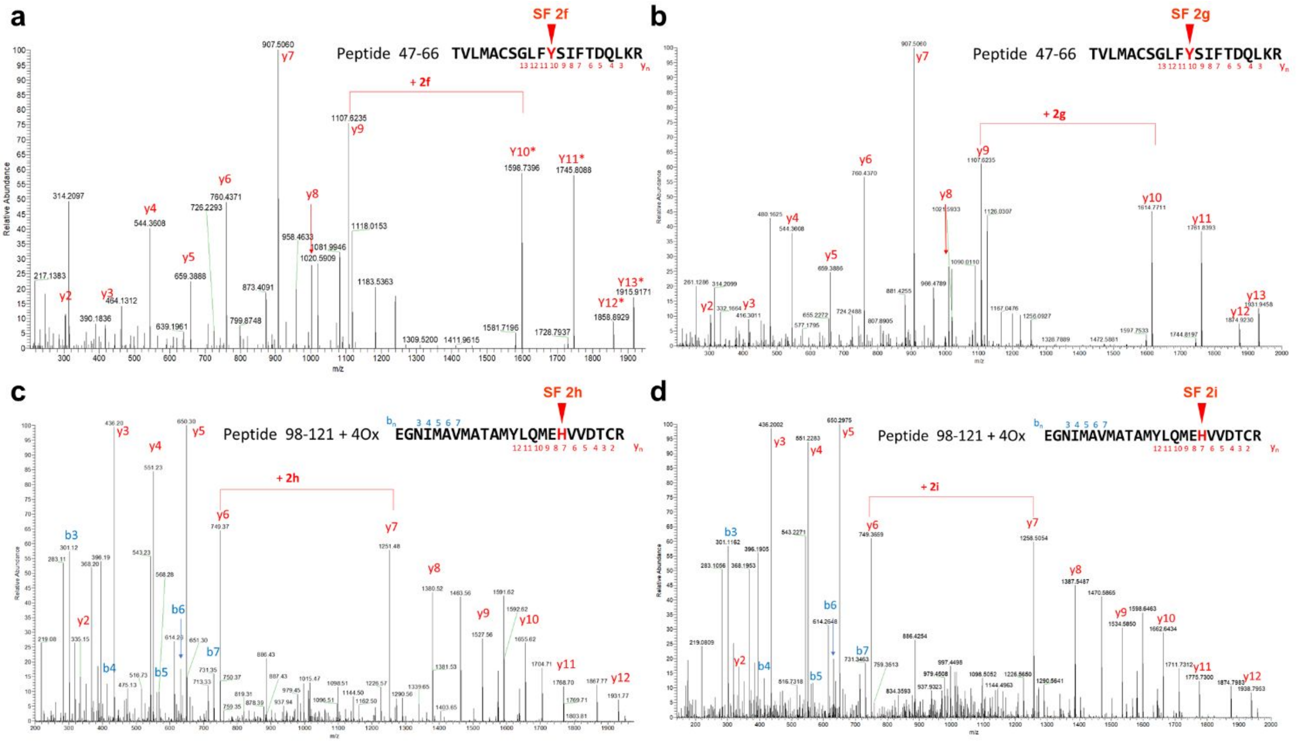

Figure S12. MS/MS spectra for identification of the sites of covalent modification for BCL6 hits 2f–i. a) MS/MS spectrum of peptide $_{47}$TVLMACSGLFY*SIFTDQLKR$_{66}$ modified by SF **2f** indicating Tyr57 as the site of covalent modification. For compound **2f**, a strong neutral loss of 45.05 was observed following MS/MS fragmentation (ions noted by the asterisk). The nature of this loss was not further investigated. b) MS/MS spectrum of peptide $_{47}$TVLMACSGLFY*SIFTDQLKR$_{66}$ modified by SF **2g** indicating Tyr57 as the site of covalent modification. c) MS/MS spectrum of peptide $_{98}$EGNIMAVMATAMYLQMEH*VVDTCR$_{121}$ modified by SF **2h** indicating His115 as the site of covalent modification. For compound **2h**, minor modification was observed at Y110; this was not investigated further. d) MS/MS spectrum of peptide $_{98}$EGNIMAVMATAMYLQMEH*VVDTCR$_{121}$ modified by SF **2i** indicating His115 as the site of covalent modification. For compound **2i**, minor modification was observed at Y110; this was not investigated further.

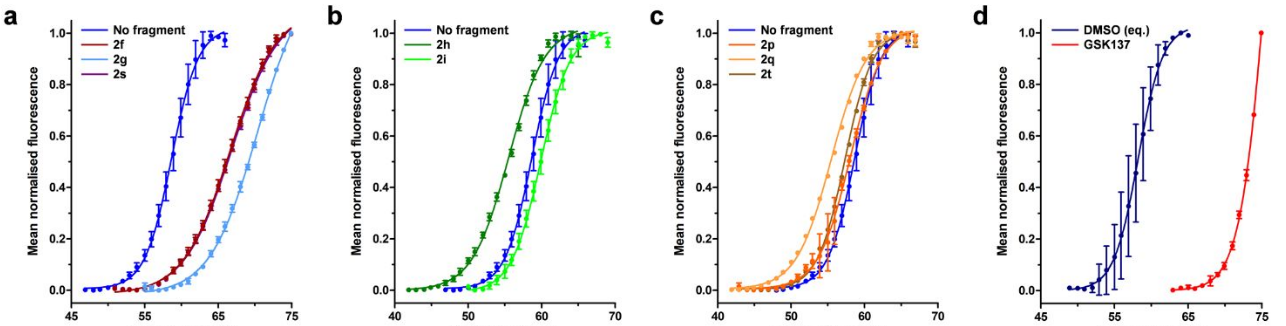

Figure S13. DSF traces for BCL6 BTB/POZ domain modified by SF fragments or binding of GSK137. a) The modification reaction of three selected *meta*-substituted benzamide fragments thermally stabilized the protein. b) The modification reaction of two azetidinyl fragments gave different effects on the thermal stabilization of BCL6. c) The modification reaction of three piperidinyl fragments thermally destabilized the protein. d) The binding of GSK137 thermally stabilized the protein.

a

b

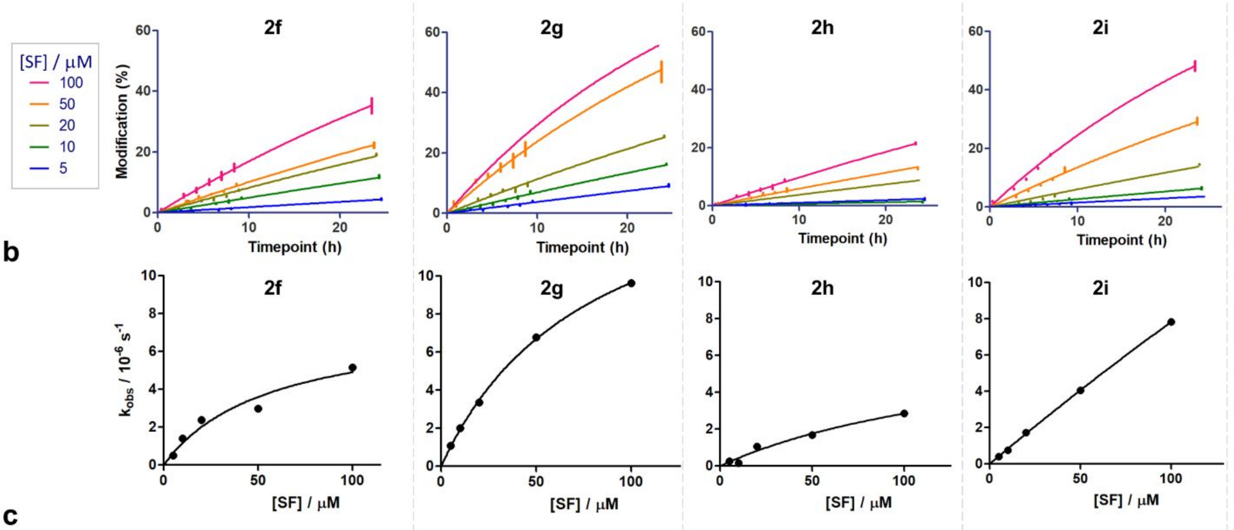

c

SF	2f	2g	2h	2i
k_{inact} / 10^{-5} s^{-1}	0.76	1.7	0.74	-
K_I / μM	56.0	80.0	160	-
(k_{inact} / K_I) / (M^{-1} s^{-1})	0.14	0.22	0.046	0.085

Figure S14. Kinetic analyzes for BCL6 hits. a) Time courses (various concentrations plotted against time and fitted to a single exponential function to determine k_{obs}) showing concentration-dependent modification of SFs **2f–i** with BCL6. b) k_{obs} measurements plotted against the measured concentrations of SFs **2f–i** to determine k_{inact} and K_I. c) Table displaying k_{inact}, K_I, and hence k_{inact}/K_I – a parameter to describe the overall modification efficiencies of SFs **2f–i**.

2. General Experimentation

2.1. Solvents; reagents; consumables; materials

Solvents were anhydrous and reagents purchased from commercial suppliers were used as received.

Plates used:

Greiner 384 white low volume plates (#784075); Greiner 384 PP F-bottom plates (#781201); Labcyte ECHO Qualified 384LDV Plus (LPL-0200).

Protein stock solutions:

Carbonic anhydrase II human, recombinant, expressed in *E. coli* (Sigma Aldrich, C6624-500UG, Lot: 069M4082V); storage buffer: 20 mM Tris, 150 mM NaCl, pH 7.5

KRas4B-G12D-C118S (1–169) produced as part of the GSK/GenScript collaboration; storage buffer: 25 mM Tris, 100 mM NaCl, 5 mM $MgCl_2$, 1 mM TCEP, pH 8.0

hBCL6-Flag-6H-Avi 3Cmut (5–129) produced as part of the GSK/GenScript collaboration; storage buffer: 20 mM Tris, 250 mM NaCl, 5 mM DTT, 5% glycerol, pH 8.5

hBCL6-Flag 3Cmut (5-129) produced in house; storage buffer: 20 mM Tris, 250 mM NaCl, 5 mM DTT, 5% glycerol, pH 8.5 – for crystallography

2.2. Liquid chromatography-mass spectrometry (LC-MS) for small molecules

LC-MS for small molecules was carried out on an Acquity UPLC CSH C-18 column (internal diameter: 50 mm × 2.1 mm, packing diameter: 1.7 µm) at 40 °C with a 0.5 µL injection volume. The UV detection was a summed signal from wavelengths between 210 nm and 350 nm. Mass detection was performed with Alternate-scan Positive and Negative Electrospray on a Waters ZQ instrument, with a scan range of 100–1000 Da or 100–1200 Da (high mass range method). Scan time: 0.27 s; inter-scan delay: 0.10 s.

LC-MS with acidic modifier (low pH method): Solvent A (0.1% *v/v* solution of formic acid in water) and solvent B (0.1% *v/v* solution of formic acid in acetonitrile). Sample was eluted with a flow rate of 1.0 mL/min using the following gradient:

Table S1. Low pH gradient for small molecule LC-MS analysis

Time / min	Solvent A (%)	Solvent B (%)
**0**	97	3
**1.5**	5	95
**1.9**	5	95
**2.0**	97	3

LC-MS with basic modifier (high pH method): Solvent A (0.1% *v/v* 10 mM ammonium bicarbonate in water adjusted to pH 10 with ammonia solution) and solvent B (0.1% *v/v* ammonia in acetonitrile). Sample was eluted with a flow rate of 1.0 mL/min using the following gradient:

Table S2. High pH gradient for small molecule LC-MS analysis

Time / min	Solvent A (%)	Solvent B (%)
**0.00**	97	3
**0.05**	97	3
**1.50**	5	95
**1.90**	5	95
**2.00**	97	3

2.3. Flash column chromatography (FCC)

FCC was conducted using Teledyne ISCO CombiFlash® R_f + apparatus with RediSep® silica cartridges. Solvent systems for FCC are reported in solvent:solvent ratios.

2.4. Mass-directed automated preparative HPLC (MDAP)

Mass directed Autoprep was carried out on a Waters® ZQ MS using alternate scan positive and negative electrospray ionization and a summed UV wavelength of 210–350 nm. Mass detection was performed over the range 150–1000 Da. Scan time: 0.5 s; inter-scan delay: 0.2 s.

MDAP with acidic modifier (low pH method): Sunfire® C18 column (100 mm × 19.0 mm, 5.00 µm packing diameter, 20.0 mL/min flow rate) using a gradient elution at ambient temperature with the mobile phases of water with 0.1% formic acid by volume (*v*/*v*) and acetonitrile containing 0.1% formic acid by volume (*v*/*v*). Flow rate: 40 mL/min.

MDAP with basic modifier (high pH method): High pH: XSelect C18 column (100 mm × 19.0 mm, 5.00 µm packing diameter, 20.0 mL/min flow rate) using a gradient elution at ambient temperature using mobile phases of water with 0.1% 10 mM ammonium bicarbonate by volume (*v*/*v*) adjusted to pH 10 with ammonia solution and 0.1% *v*/*v* ammonia in acetonitrile. Flow rate: 40 mL/min.

The gradient of acetonitrile required to elute product was determined by the LC-MS retention time of the desired material. The following methods were selected dependent on the retention time of desired material:

Table S3. MDAP Methods

Method	LC-MS t_R	Acetonitrile (%)
**A**	0.40–0.65	0–30
**B**	0.65–0.90	15–55
**C**	0.90–1.16	30–85
**D**	1.16–1.40	50–99
**E**	1.40–2.00	80–99

2.5. Nuclear magnetic resonance (NMR) spectroscopy

NMR spectroscopy was carried out at ambient temperature using standard pulse methods on a Bruker AVII600 (1H = 400 and 600 MHz) in DMSO-d_6 and referenced to residual undeuterated solvent. Multiplicity is reported as follows: s = singlet, d = doublet, t = triplet, q = quartet, m = multiplet etc. All spin-spin coupling constants (*J*) are reported in hertz to the nearest 0.1 Hz.

2.6. Infrared (IR) spectroscopy

IR spectra were recorded using a Perkin Elmer® spectrum 1 machine. Absorption maxima (ν_{max}) are reported in wavenumbers (cm^{-1}).

2.7. Centrifuge

Plates were centrifuged using a Sorvall Legend RT (401198833) model.

2.8. Intact protein LC-MS

Intact protein masses were recorded by LC-MS using an Agilent G230B time-of-flight (ToF) Accurate Mass Series mass spectrometer, interfaced with an Agilent 1290 infinity II series column oven (G7116B) and an Agilent 1290 infinity II series liquid chromatography high speed binary pump (G7120A). Protein samples were injected using an Agilent 1290 infinity II series multisampler with dual needles (G7167B) with a 0.5–10 µL injection volume and at a temperature of 4–20 °C. Chromatography was carried out on an Agilent Bio-HPLC PLRP-S (1000 Å, 5 µm × 50 mm × 1.0 mm, PL1312-1502) reverse phase HPLC column at 70 °C. The sample was eluted at 0.5 mL/min using a gradient system from Solvent A (water, 0.2% (*v*/*v*) formic acid) to Solvent B (acetonitrile, 0.2% (*v*/*v*) formic acid) according to the following conditions:

Table S4. Elution gradient used for intact protein LC-MS

Time / min	Solvent B (%)
**0.00**	20
**0.60**	20
**0.61**	50
**1.00**	100
**1.20**	100
**1.21**	20

The eluent was injected directly into an Agilent ToF mass spectrometer (G6230B) using a dual AJS ESI source and scanning between 600–3200 Da with a scan rate of 1.2 s in positive mode. The following MS parameters were used: capillary voltage limit: 4000 V; desolvation temperature: 350 °C; drying gas flow: 10 L/min. Data acquisition was carried out in 2 GHz Extended Dynamic range mode. Spectra were processed using Mass Hunter Qualitative Analysis™ B06.00 (Agilent) with the Maximum Entropy method employed. The total ion chromatograms (TIC) were extracted (region containing protein) and the summed scans were deconvoluted (using a maximum entropy algorithm) over a *m*/*z* range with an expected mass range dependent on the protein:

Table S5. Deconvolution conditions for proteins

Protein	*m/z* range	Expected Mass Range
**CAII**	850–2000	23500–32500
**KRAS4B^{G12D}**	850–2000	14000–23000
**BCL6**	850–2000	13000–22000

The deconvoluted spectra were exported as csv files and analyzed using R Studio (Version 3.6.3) software to generate PDF files of the spectra.[1] The median of the protein only controls were subtracted from the sample spectra to remove baseline signal. The peak height for unmodified protein and protein modified by a reactive fragment were recorded and used to calculate percentage modification using equation (3):

$$\% = ((\text{intensity of modified protein})/(\text{intensity of protein only}+(\text{intensity of modified protein})))*100 \qquad (3)$$

3. High-throughput chemistry (HTC) and direct-to-biology (D2B) protocols

3.1. HTC protocol trial

Six Labcyte 384LDV plates were charged with amines **S1**–**12** (10 mM) in DMSO (5 µL per well). To plates 1–3 was added a stock solution of OSu **1a**, **2a**, or **3a** (10 mM) and *N*-ethylmorpholine (NEM) (30 mM) in DMSO (5 µL). To plates 4–6 plate was added a stock solution of OSu **1a**, **2a**, or **3a** (10 mM and *N*-ethylmorpholine (NEM) (30 mM) in DMSO:water (9:1) (5 µL per well). The plates were sealed, centrifuged (1 min, 1000 rpm), and allowed to sit at room temperature for 1 h. Reaction concentrations: 5 mM amine; 5 mM OSu ester; 15 mM NEM. After the reaction, each individual well was analyzed by small molecule LC-MS to assess and compare conversions by area under curve of the product peak relative to the total area.

Table S6. Comparison of reactive moieties 1–3 under dry DMSO and DMSO:water (9:1) conditions. Values show percentage conversions by LC-MS.

	Reactive Moiety 1		Reactive Moiety 2		Reactive Moiety 3	
**Amine**	DMSO	DMSO:water (9:1)	DMSO	DMSO:water (9:1)	DMSO	DMSO:water (9:1)
**S1**	43	44	83	65	100	89
**S2**	56	48	93	67	82	86
**S3**	88	66	79	87	95	100
**S4**	90	86	93	97	98	98
**S5**	88	67	79	83	100	94
**S6**	78	64	84	88	100	100
**S7**	58	52	86	73	85	94
**S8**	60	47	73	54	88	92
**S9**	86	86	79	83	90	98
**S10**	88	72	91	94	93	93
**S11**	54	43	86	88	93	94
**S12**	85	62	94	86	96	95

3.2. Selection of 352 amine functionalized fragments

Selection was based on the molecular clustering approach used for the PhABit libraries.[2,3] A diverse set of compounds containing only one aliphatic amine group was considered. The starting set for the selection was the solution sample store of the GSK compound collection. However, compounds were also checked for the availability in eMolecules and Enamine building block databases. The selection was then carried out using Pipeline Pilot (Version 20.1.0.2208) software.[4] Initial selection criteria were applied: GSK compounds having at least one sample with a concentration of 10 mM in DMSO or above, and with at least 150 µL available. Unstable compounds and compounds with other liabilities were removed using proprietary GSK filters. Further selection criteria were then applied: aromatic ring count≤2; HBDs/HBAs≤4; heavy atoms≤15; 150<M_W≤250; BioByte cLogP<9; 1 aliphatic amine group.[5] The structures were tagged based on 6 aliphatic amine types, differentiating based on primary/secondary, hindered/non-hindered and cyclic/non-cyclic. The amines were also tagged by the calculated pK_a using ChemAxon pK_a.[6] All amines having acidic pK_a<7 and secondary hindered aliphatic amines were excluded. A ChemAxon LibMCS clustering was performed on the remaining molecules.[7] Since the aim of the current selection was to choose compounds which can have analogues, all compounds were excluded which did not belong to a cluster with at least 4 members. A diverse selection was then performed on the remaining molecules using the Diverse Molecules component from Pipeline Pilot and ECFP4 fingerprinting.[8] A 352-membered library was then selected from the remaining molecules. The selected amines (10 mM, 5 µL per well/screen) were ordered from GSK's solution stores in Labcyte 384LDV plates:

1 FS(=O)(=O)c1ccc(CC(=O)N2CCCCC2Cc2ccncc2)cc1
2 FS(=O)(=O)c1ccc(CC(=O)N2CCCCC2c2cccnc2)cc1
3 COc1cccc(c1)C(CNC(=O)OC(C)(C)C)NC(=O)Cc1ccc(cc1)S(F)(=O)=O
4 Cc1noc(n1)C1CCN(CC1)C(=O)Cc1ccc(cc1)S(F)(=O)=O

5 FS(=O)(=O)c1ccc(CC(=O)N2CCC(CC2)N2CCCCC2)cc1
6 Cc1noc(C)c1-c1cccc(CNC(=O)Cc2ccc(cc2)S(F)(=O)=O)c1
7 FS(=O)(=O)c1ccc(CC(=O)N2CCC3(CCCNC3=O)CC2)cc1
8 CC(=O)N1CCC(CC1)N(C1CC1)C(=O)Cc1ccc(cc1)S(F)(=O)=O
9 CN(Cc1ccc(cc1)-c1ccncc1)C(=O)Cc1ccc(cc1)S(F)(=O)=O
10 CN(Cc1cn2CCCCc2n1)C(=O)Cc1ccc(cc1)S(F)(=O)=O
11 FS(=O)(=O)c1ccc(CC(=O)N2CCN(CC2)c2ncccn2)cc1
12 CN(C(C)=O)c1cccc(CNC(=O)Cc2ccc(cc2)S(F)(=O)=O)c1
13 CC(C)CN1CCOC(CNC(=O)Cc2ccc(cc2)S(F)(=O)=O)C1
14 CC(C)(C)NC(=O)C1CCCCN1C(=O)Cc1ccc(cc1)S(F)(=O)=O
15 OC[C@H](Cc1ccc(O)cc1)NC(=O)Cc1ccc(cc1)S(F)(=O)=O
16 C[C@@H]([C@H](O)c1ccc(O)cc1)N(C)C(=O)Cc1ccc(cc1)S(F)(=O)=O
17 CCOCCN1CCN(CC1C)C(=O)Cc1ccc(cc1)S(F)(=O)=O
18 FS(=O)(=O)c1ccc(CC(=O)N[C@@H]2COCC[C@@H]2OCC2CC2)cc1
19 CNC(=O)[C@@H]1CN(C[C@H]1COC)C(=O)Cc1ccc(cc1)S(F)(=O)=O
20 COCCOC1CCC11CCN(CC1)C(=O)Cc1ccc(cc1)S(F)(=O)=O
21 FS(=O)(=O)c1ccc(CC(=O)NC2CCN(CC2)C2CCCCC2)cc1
22 CN1CCN(CC1)C1CCN(CC1)C(=O)Cc1ccc(cc1)S(F)(=O)=O
23 FS(=O)(=O)c1ccc(CC(=O)N2CCC3(CCc4ccccc34)CC2)cc1
24 CCc1ccccc1NC(=O)CN(C)C(=O)Cc1ccc(cc1)S(F)(=O)=O
25 FS(=O)(=O)c1ccc(CC(=O)NCC(c2ccccc2)c2ccccc2)cc1
26 FS(=O)(=O)c1ccc(CC(=O)NCCc2nc(no2)-c2ccccc2)cc1
27 OC1CCOC2(CCN(CC2)C(=O)Cc2ccc(cc2)S(F)(=O)=O)C1
28 Cn1c(CCNC(=O)Cc2ccc(cc2)S(F)(=O)=O)nc2ccccc12
29 FS(=O)(=O)c1ccc(CC(=O)N2CCC(C2)Oc2cnccn2)cc1
30 CC(C)NC(=O)c1cccc(CNC(=O)Cc2ccc(cc2)S(F)(=O)=O)c1
31 CC(=O)Nc1ccn(n1)C1CCN(C1)C(=O)Cc1ccc(cc1)S(F)(=O)=O
32 CN(C[C@@H]1C[C@H](F)CN1CC(N)=O)C(=O)Cc1ccc(cc1)S(F)(=O)=O
33 CN(C1CN(C1)C(=O)Cc1ccc(cc1)S(F)(=O)=O)c1ccccc1
34 FS(=O)(=O)c1ccc(CC(=O)N2CCCC2CN2CCCCC2)cc1
35 FS(=O)(=O)c1ccc(CC(=O)NC2CCc3ccccc3NC2=O)cc1
36 Cc1onc(c1CNC(=O)Cc1ccc(cc1)S(F)(=O)=O)-c1ccccc1
37 FS(=O)(=O)c1ccc(CC(=O)NCCn2c3ccccc3[nH]c2=O)cc1
38 FS(=O)(=O)c1ccc(CC(=O)N2CCN(CC2)C2CCCCC2)cc1
39 CC(C)(C)OC(=O)N1CC(CNC(=O)Cc2ccc(cc2)S(F)(=O)=O)Cn2nccc2C1
40 FS(=O)(=O)c1ccc(CC(=O)N2CCN(CC2)c2ncccn2)cc1
41 CN(C)S(=O)(=O)N1CCN(CC1)C(=O)Cc1ccc(cc1)S(F)(=O)=O
42 CCN(CC)C(=O)C1CCCN(C1)C(=O)Cc1ccc(cc1)S(F)(=O)=O
43 CC1CN(CCN1c1ccccc1)C(=O)Cc1ccc(cc1)S(F)(=O)=O
44 FS(=O)(=O)c1ccc(CC(=O)NC(Cc2ccccc2)c2ccccc2)cc1
45 FS(=O)(=O)c1ccc(CC(=O)NCc2nnc(o2)-c2ccccc2)cc1
46 FS(=O)(=O)c1ccc(CC(=O)NCc2nccc3ccccc23)cc1
47 CS(=O)(=O)Nc1cccc(CNC(=O)Cc2ccc(cc2)S(F)(=O)=O)c1
48 CC(NC(=O)Cc1ccc(cc1)S(F)(=O)=O)c1cccc(c1)S(N)(=O)=O
49 Oc1ccc2CCN(CCc2c1)C(=O)Cc1ccc(cc1)S(F)(=O)=O
50 CN1CCN(C2CCN(CC2)C(=O)Cc2ccc(cc2)S(F)(=O)=O)C1=O
51 CN(Cc1cn(C)c(=O)n(C)c1=O)C(=O)Cc1ccc(cc1)S(F)(=O)=O
52 FS(=O)(=O)c1ccc(CC(=O)NCc2ccc3ccccc3n2)cc1

53 COCC1CN(CC11CCN(CC1)C(=O)OC(C)(C)C)C(=O)Cc1ccc(cc1)S(F)(=O)=O
54 Cn1nc(cc1O)C1CCN(C1)C(=O)Cc1ccc(cc1)S(F)(=O)=O
55 FS(=O)(=O)c1ccc(CC(=O)NC(c2ccccc2)c2ccccn2)cc1
56 CC(C)(C)OC(=O)N1CCC(CC1)[C@@H]1NC(=O)CC[C@H]1NC(=O)Cc1ccc(cc1)S(F)(=O)=O
57 CN(CC1CCN(CCO)CC1)C(=O)Cc1ccc(cc1)S(F)(=O)=O
58 FS(=O)(=O)c1ccc(CC(=O)NCc2ccc(cc2)-c2cncnc2)cc1
59 CN1CCC(CC1)C1CCCCN1C(=O)Cc1ccc(cc1)S(F)(=O)=O
60 Cc1nc(CN2CCCC(C2)NC(=O)Cc2ccc(cc2)S(F)(=O)=O)no1
61 FS(=O)(=O)c1ccc(CC(=O)N2CCCN(CC2)c2cccnn2)cc1
62 Cc1nnc2CN(CCn12)C1CN(C1)C(=O)Cc1ccc(cc1)S(F)(=O)=O
63 FS(=O)(=O)c1ccc(CC(=O)NCc2ccc(cc2)N2CCOCC2)cc1
64 FS(=O)(=O)c1ccc(CC(=O)NC2CCN(Cc3cccnc3)C2)cc1
65 Cc1noc(COC2CCN(CC2)C(=O)Cc2ccc(cc2)S(F)(=O)=O)n1
66 CC(C)c1nc2CN(CCc2n1C)C(=O)Cc1ccc(cc1)S(F)(=O)=O
67 CCc1nnc([nH]1)C1CCCN1C(=O)Cc1ccc(cc1)S(F)(=O)=O
68 FS(=O)(=O)c1ccc(CC(=O)NCCn2ccnc2-c2ccccc2)cc1
69 CN(Cc1cccc(c1)-c1cncnc1)C(=O)Cc1ccc(cc1)S(F)(=O)=O
70 COCCN(C1CCN(C)CC1)C(=O)Cc1ccc(cc1)S(F)(=O)=O
71 FS(=O)(=O)c1ccc(CC(=O)N2CCN(CC2)c2ccccc2)cc1
72 Cc1noc(C)c1CN1CCN(CC1)C(=O)Cc1ccc(cc1)S(F)(=O)=O
73 Oc1ccc(cc1)C1CCCN1C(=O)Cc1ccc(cc1)S(F)(=O)=O
74 CC(C)(C)OC(=O)N1CC(CCNC(=O)Cc2ccc(cc2)S(F)(=O)=O)n2nccc2C1
75 Cc1cccn2cc(CNC(=O)Cc3ccc(cc3)S(F)(=O)=O)nc12
76 Cn1ccnc1CN1CCCN(CC1)C(=O)Cc1ccc(cc1)S(F)(=O)=O
77 FS(=O)(=O)c1ccc(CC(=O)N2CCC3(CCc4ccccc34)CC2)cc1
78 FS(=O)(=O)c1ccc(CC(=O)NCc2ccccc2-n2cccn2)cc1
79 CC(C)N([C@@H](CO)c1ccccc1)C(=O)Cc1ccc(cc1)S(F)(=O)=O
80 FS(=O)(=O)c1ccc(CC(=O)NC(c2ccccc2)c2cccnc2)cc1
81 CN(C[C@H](O)c1cccc(O)c1)C(=O)Cc1ccc(cc1)S(F)(=O)=O
82 FS(=O)(=O)c1ccc(CC(=O)N2CCCCC2c2ccncc2)cc1
83 FS(=O)(=O)c1ccc(CC(=O)N[C@H]2COCC[C@H]2Oc2ccccc2)cc1
84 C[C@@H](NC(=O)Cc1ccc(cc1)S(F)(=O)=O)[C@@H](O)c1cccc(O)c1
85 CC(C)(C)OC(=O)N1CCN(CC1)C(=O)C1CCN(CC1)C(=O)Cc1ccc(cc1)S(F)(=O)=O
86 Cc1c(cc(c(O)c1C(=O)Nc1ccccc1S(F)(=O)=O)C(C)(C)C)N(=O)=O
87 OCC1C(OCCN1C(=O)Cc1ccc(cc1)S(F)(=O)=O)c1ccccc1
88 OC1(CCN(CC1)C(=O)Cc1ccc(cc1)S(F)(=O)=O)c1ccccc1
89 Cc1nc(no1)C1CCCCN1C(=O)Cc1ccc(cc1)S(F)(=O)=O
90 Fc1ccc(OC2CN(C2)C(=O)Cc2ccc(cc2)S(F)(=O)=O)cc1F
91 FS(=O)(=O)c1ccc(CC(=O)NCc2ccc(Oc3ccccc3)cc2)cc1
92 CN(C)C(=O)[C@H](Cc1ccccc1)NC(=O)Cc1ccc(cc1)S(F)(=O)=O
93 CNC(=O)c1ccccc1CNC(=O)Cc1ccc(cc1)S(F)(=O)=O
94 CC1CC(C)CN(CCCNC(=O)Cc2ccc(cc2)S(F)(=O)=O)C1
95 CC(C)(C)OC(=O)N1CCCC(C1)C1CCCCN1C(=O)Cc1ccc(cc1)S(F)(=O)=O
96 FS(=O)(=O)c1ccc(CC(=O)N2CCCN(Cc3ccccc3)CC2)cc1
97 NS(=O)(=O)Cc1cccc(CNC(=O)Cc2ccc(cc2)S(F)(=O)=O)c1
98 FS(=O)(=O)c1ccc(CC(=O)NCc2ccc(CN3CCCC3)cc2)cc1
99 Cc1nnc(o1)C1COCCN1C(=O)Cc1ccc(cc1)S(F)(=O)=O
100 FS(=O)(=O)c1ccc(CC(=O)NC(C2CCC2)c2ccccc2)cc1

101 Cn1cc(cn1)[C@H]1NC(=O)CC[C@@H]1NC(=O)Cc1ccc(cc1)S(F)(=O)=O

102 FS(=O)(=O)c1ccc(CC(=O)NCCc2nc3ccccc3[nH]2)cc1

103 FS(=O)(=O)c1ccc(CC(=O)N2CCN(CCn3ccnc3)CC2)cc1

104 OCC1(Cc2ccccc2)CCCN1C(=O)Cc1ccc(cc1)S(F)(=O)=O

105 CNC(=O)c1cccc(CNC(=O)Cc2ccc(cc2)S(F)(=O)=O)c1

106 CC(C)c1nnc2CN(CCn12)C(=O)Cc1ccc(cc1)S(F)(=O)=O

107 CC1(CCCN(C1)C(=O)Cc1ccc(cc1)S(F)(=O)=O)c1cc(O)[nH]n1

108 FS(=O)(=O)c1ccc(CC(=O)NCc2ccc(Oc3ccccn3)cc2)cc1

109 Cn1cc(cn1)[C@H]1OCC[C@@H]1NC(=O)Cc1ccc(cc1)S(F)(=O)=O

110 FS(=O)(=O)c1ccc(CC(=O)NCc2ccccc2-c2cccnc2)cc1

111 Oc1ccccc1N1CCN(CC1)C(=O)Cc1ccc(cc1)S(F)(=O)=O

112 FS(=O)(=O)c1ccc(CC(=O)N2CCN(CC2)C(=O)c2ccccn2)cc1

113 OC[C@@H]1Cc2ccccc2CN1C(=O)Cc1ccc(cc1)S(F)(=O)=O

114 OC(C1COCCN1C(=O)Cc1ccc(cc1)S(F)(=O)=O)c1ccccc1

115 CC1CCN(CC2CN(CCO2)C(=O)Cc2ccc(cc2)S(F)(=O)=O)CC1

116 FS(=O)(=O)c1ccc(CC(=O)N2CCCC2CN2CCCCC2)cc1

117 Cn1nccc1C1CCCN(C1)C(=O)Cc1ccc(cc1)S(F)(=O)=O

118 CCC1CN(CCN1CC(C)O)C(=O)Cc1ccc(cc1)S(F)(=O)=O

119 CS(=O)(=O)c1ccc(CCNC(=O)Cc2ccc(cc2)S(F)(=O)=O)cc1

120 Oc1cccc(c1)C1CCN(CC1)C(=O)Cc1ccc(cc1)S(F)(=O)=O

121 FS(=O)(=O)c1ccc(CC(=O)N2CCN(CC2)c2ncccn2)cc1

122 OC1CCCC1N1CCN(CC1)C(=O)Cc1ccc(cc1)S(F)(=O)=O

123 Fc1cccc(c1)C1CCN(C1)C(=O)Cc1ccc(cc1)S(F)(=O)=O

124 CN(CC1CCn2ccnc2C1)C(=O)Cc1ccc(cc1)S(F)(=O)=O

125 CN1CCN(CC1c1nccn1C)C(=O)Cc1ccc(cc1)S(F)(=O)=O

126 CCS(=O)(=O)N1CCN(C(C)C1)C(=O)Cc1ccc(cc1)S(F)(=O)=O

127 Cn1cc(cn1)[C@@H]1CN(C[C@H]1CO)C(=O)Cc1ccc(cc1)S(F)(=O)=O

128 CC(C)(C)OC(=O)N1CCC(C1)OC1CCN(CC1)C(=O)Cc1ccc(cc1)S(F)(=O)=O

129 CN(C)c1cc2CN(CCc2nn1)C(=O)Cc1ccc(cc1)S(F)(=O)=O

130 CC(C)(C)OC(=O)N1CCOC2(C1)COCCN(C2)C(=O)Cc1ccc(cc1)S(F)(=O)=O

131 FS(=O)(=O)c1ccc(CC(=O)NCc2cccc3cnccc23)cc1

132 CC(C)Oc1ccccc1CNC(=O)Cc1ccc(cc1)S(F)(=O)=O

133 Cc1nnc(o1)C1CN(CCO1)C(=O)Cc1ccc(cc1)S(F)(=O)=O

134 FS(=O)(=O)c1ccc(CC(=O)NC2CCN(CC2)c2ncccn2)cc1

135 FS(=O)(=O)c1ccc(CC(=O)NCc2cccc3cccnc23)cc1

136 FS(=O)(=O)c1ccc(CC(=O)N2CCC(Cc3ccccc3)CC2)cc1

137 FS(=O)(=O)c1ccc(CC(=O)N2CCC(Cc3ccccc3)CC2)cc1

138 Cc1ccccc1C1CCCN1C(=O)Cc1ccc(cc1)S(F)(=O)=O

139 FS(=O)(=O)c1ccc(CC(=O)N2CCC(CC2)C(=O)N2CCCC2)cc1

140 Cc1noc(n1)C(NC(=O)Cc1ccc(cc1)S(F)(=O)=O)C1CCOCC1

141 FS(=O)(=O)c1ccc(CC(=O)N[C@H]2CC[C@@H](CC2)Oc2ncccn2)cc1

142 Oc1cccc(c1)N1CCN(CC1)C(=O)Cc1ccc(cc1)S(F)(=O)=O

143 Cc1noc(C)c1-c1cccc(CNC(=O)Cc2ccc(cc2)S(F)(=O)=O)c1

144 FS(=O)(=O)c1ccc(CC(=O)NCc2cncc3ccccc23)cc1

145 FS(=O)(=O)c1ccc(CC(=O)NCc2cccc(c2)-c2nnn[nH]2)cc1

146 FS(=O)(=O)c1ccc(CC(=O)NCc2cc(no2)-c2ccccc2)cc1

147 CC(=O)Nc1cccc(CNC(=O)Cc2ccc(cc2)S(F)(=O)=O)c1

148 FS(=O)(=O)c1ccc(CC(=O)NCc2ccc3ncccc3c2)cc1

149 FS(=O)(=O)c1ccc(CC(=O)NCc2cccc(Cn3ccnc3)c2)cc1

150 FS(=O)(=O)c1ccc(CC(=O)NC(c2ccccc2)c2ccccn2)cc1

151 FS(=O)(=O)c1ccc(CC(=O)NCc2ccc(cc2)-c2ccccn2)cc1

152 CC(C)(C)OC(=O)N1C(CNC(=O)Cc2ccc(cc2)S(F)(=O)=O)CCc2ccccc12

153 C[C@@H]([C@H](O)c1ccc(O)cc1)N(C)C(=O)Cc1ccc(cc1)S(F)(=O)=O

154 COC[C@H]1CN(C[C@@H]1c1ccccn1)C(=O)Cc1ccc(cc1)S(F)(=O)=O

155 CS(=O)(=O)N1CCCC(CNC(=O)Cc2ccc(cc2)S(F)(=O)=O)C1

156 FS(=O)(=O)c1ccc(CC(=O)N2CCCC(C2)c2ccccc2)cc1

157 FS(=O)(=O)c1ccc(CC(=O)N2CCCCC2Cc2ccccn2)cc1

158 CC(C)(C)OC(=O)N1CC(CCNC(=O)Cc2ccc(cc2)S(F)(=O)=O)Cn2nccc2C1

159 Oc1cccc(c1)N1CCN(CC1)C(=O)Cc1ccc(cc1)S(F)(=O)=O

160 NS(=O)(=O)c1cccc(CNC(=O)Cc2ccc(cc2)S(F)(=O)=O)c1

161 COc1ccccc1C1CCCN1C(=O)Cc1ccc(cc1)S(F)(=O)=O

162 Cn1nc2CCC(CCn2c1=O)NC(=O)Cc1ccc(cc1)S(F)(=O)=O

163 FS(=O)(=O)c1ccc(CC(=O)N2CCC(CC2)Oc2ccccc2)cc1

164 CNC(=O)c1ccc(CN(C)C(=O)Cc2ccc(cc2)S(F)(=O)=O)cc1

165 CC(NC(=O)Cc1ccc(cc1)S(F)(=O)=O)c1ccc(cc1)-n1cncn1

166 NC(=O)[C@H](Cc1ccccc1)NC(=O)Cc1ccc(cc1)S(F)(=O)=O

167 Cc1nc2ccccn2c1CNC(=O)Cc1ccc(cc1)S(F)(=O)=O

168 FS(=O)(=O)c1ccc(CC(=O)N[C@H]2CCO[C@@H]2c2cccnc2)cc1

169 FS(=O)(=O)c1ccc(CC(=O)NCc2nc(no2)-c2cccnc2)cc1

170 FS(=O)(=O)c1ccc(CC(=O)N2CCCC(Cc3ccccc3)C2)cc1

171 Cc1noc(n1)[C@@H]1CN(C[C@H]1CO)C(=O)Cc1ccc(cc1)S(F)(=O)=O

172 CN(Cc1cccc(c1)-n1cccn1)C(=O)Cc1ccc(cc1)S(F)(=O)=O

173 Cn1nnc2cc(CNC(=O)Cc3ccc(cc3)S(F)(=O)=O)ccc12

174 NC(=O)[C@@H](Cc1ccccc1)NC(=O)Cc1ccc(cc1)S(F)(=O)=O

175 FS(=O)(=O)c1ccc(CC(=O)N2CCC(CC2)N2CCCNC2=O)cc1

176 FS(=O)(=O)c1ccc(CC(=O)NCC(N2CCOCC2)c2ccco2)cc1

177 CCc1ncc2CCN(Cc2n1)C(=O)Cc1ccc(cc1)S(F)(=O)=O

178 FS(=O)(=O)c1ccc(CC(=O)NCc2ccc(cc2)N2CCCC2=O)cc1

179 FS(=O)(=O)c1ccc(CC(=O)NCCNC(=O)Nc2ccccc2)cc1

180 COc1ccnc(c1)N1CCN(CC1)C(=O)Cc1ccc(cc1)S(F)(=O)=O

181 OC(C1CCN(CC1)C(=O)Cc1ccc(cc1)S(F)(=O)=O)C(=O)NC1CC1

182 FS(=O)(=O)c1ccc(CC(=O)N2CCC(CC2)N2CCNC2=O)cc1

183 Cn1nccc1C1CCN(CC1)C(=O)Cc1ccc(cc1)S(F)(=O)=O

184 CC(C)N1CCN(CC1)C1CCN(C1)C(=O)Cc1ccc(cc1)S(F)(=O)=O

185 FS(=O)(=O)c1ccc(CC(=O)N2CCCC(C2)C(=O)N2CCOCC2)cc1

186 FS(=O)(=O)c1ccc(CC(=O)N2CCOCC2c2cccnc2)cc1

187 CN(C)S(=O)(=O)N1CCN(CC1)C(=O)Cc1ccc(cc1)S(F)(=O)=O

188 FS(=O)(=O)c1ccc(CC(=O)NCc2ccc(cc2)-n2cccn2)cc1

189 CN(Cc1ncccn1)C1CCN(C1)C(=O)Cc1ccc(cc1)S(F)(=O)=O

190 FS(=O)(=O)c1ccc(CC(=O)N2CCN(CC2)c2ccccc2)cc1

191 CC(C)(C)OC(=O)N1CCC(CC1)N1CCN(CC1)C(=O)Cc1ccc(cc1)S(F)(=O)=O

192 FS(=O)(=O)c1ccc(CC(=O)N[C@@H]2CCC[C@H]2OCc2ccccc2)cc1

193 CN1CCCC1CC1CCCCN1C(=O)Cc1ccc(cc1)S(F)(=O)=O

194 FS(=O)(=O)c1ccc(CC(=O)N2CCCC2Cc2ccccc2)cc1

195 FS(=O)(=O)c1ccc(CC(=O)N2CCN(CC2)c2ccncc2)cc1

196 FS(=O)(=O)c1ccc(CC(=O)N2CCC(CC2)C2CCOCC2)cc1

197 FS(=O)(=O)c1ccc(CC(=O)N2CCC(C2)Oc2cccnc2)cc1
198 Oc1ccccc1N1CCN(CC1)C(=O)Cc1ccc(cc1)S(F)(=O)=O
199 CC(C)(C)OC(=O)N1CCN(CC1Cc1ccccc1)C(=O)Cc1ccc(cc1)S(F)(=O)=O
200 Cc1[nH]c2ccccc2c1CNC(=O)Cc1ccc(cc1)S(F)(=O)=O
201 CCC1CN(CC1N1CCOCC1)C(=O)Cc1ccc(cc1)S(F)(=O)=O
202 COc1cccc(OC)c1CNC(=O)Cc1ccc(cc1)S(F)(=O)=O
203 FS(=O)(=O)c1ccc(CC(=O)NCC2CN(Cc3cncnc3)C2)cc1
204 Cn1nccc1N1CCCC(NC(=O)Cc2ccc(cc2)S(F)(=O)=O)C1=O
205 FS(=O)(=O)c1ccc(CC(=O)N2CCC(C2)Oc2cccnn2)cc1
206 CN(Cc1cccc(c1)C(N)=O)C(=O)Cc1ccc(cc1)S(F)(=O)=O
207 FS(=O)(=O)c1ccc(CC(=O)N2CCn3c(C2)nnc3-c2ccccc2)cc1
208 Cn1cc(CN(C2CCCCC2)C(=O)Cc2ccc(cc2)S(F)(=O)=O)cn1
209 CC(C)(C)NC(=O)[C@@H]1CN(CCN1C(=O)Cc1ccc(cc1)S(F)(=O)=O)C(=O)OC(C)(C)C
210 Cc1noc(C)c1-c1ccc(CNC(=O)Cc2ccc(cc2)S(F)(=O)=O)cc1
211 FS(=O)(=O)c1ccc(CC(=O)NCc2cccc(c2)-c2ccccn2)cc1
212 Cn1cc(CN2CCCN(CC2)C(=O)Cc2ccc(cc2)S(F)(=O)=O)cn1
213 FS(=O)(=O)c1ccc(CC(=O)N2CCC(CC2)c2c[nH]c3ccccc23)cc1
214 FS(=O)(=O)c1ccc(CC(=O)NC(c2ccccc2)c2ccncc2)cc1
215 CCC1CN(CCC1NC(=O)Cc1ccc(cc1)S(F)(=O)=O)C(=O)C(C)C
216 FS(=O)(=O)c1ccc(CC(=O)N2CCCC2CN2CCOCC2)cc1
217 COc1ccnc(OC)c1CNC(=O)Cc1ccc(cc1)S(F)(=O)=O
218 FS(=O)(=O)c1ccc(CC(=O)NCc2cnn(c2)-c2ccccc2)cc1
219 FS(=O)(=O)c1ccc(CC(=O)N2CCC(CC2)c2ccccc2)cc1
220 OC[C@@H](Cc1c[nH]c2ccccc12)NC(=O)Cc1ccc(cc1)S(F)(=O)=O
221 Cn1cnnc1C1CN(CCO1)C(=O)Cc1ccc(cc1)S(F)(=O)=O
222 CN(Cc1nc2ccsc2c(=O)[nH]1)C(=O)Cc1ccc(cc1)S(F)(=O)=O
223 FS(=O)(=O)c1ccc(CC(=O)N2CCC(CC2)c2ccccn2)cc1
224 Cn1nc(cc1O)C1CCCN1C(=O)Cc1ccc(cc1)S(F)(=O)=O
225 NC(=O)c1cnn2CC(CNC(=O)Cc3ccc(cc3)S(F)(=O)=O)CCc12
226 FS(=O)(=O)c1ccc(CC(=O)N2CCC(CCc3ccccc3)C2)cc1
227 FS(=O)(=O)c1ccc(CC(=O)N2CCN(CC2)C(=O)C2CCCCC2)cc1
228 CCc1nnc([nH]1)C1CCN(CC1)C(=O)Cc1ccc(cc1)S(F)(=O)=O
229 OC(CN1CCN(CC1)C(=O)Cc1ccc(cc1)S(F)(=O)=O)C1CC1
230 FS(=O)(=O)c1ccc(CC(=O)N2CCN(CC2)c2cnccn2)cc1
231 FS(=O)(=O)c1ccc(CC(=O)NC(c2ccccc2)c2ccccc2)cc1
232 CN(Cc1ccc2nccn2c1)C(=O)Cc1ccc(cc1)S(F)(=O)=O
233 Fc1ccc(cc1)N1CC(CC1=O)NC(=O)Cc1ccc(cc1)S(F)(=O)=O
234 FS(=O)(=O)c1ccc(CC(=O)N2CCCC3(CCCNC3=O)C2)cc1
235 FS(=O)(=O)c1ccc(CC(=O)N[C@@H]2COCC[C@H]2Oc2ccccn2)cc1
236 Oc1cccc(c1)-c1cccc(CNC(=O)Cc2ccc(cc2)S(F)(=O)=O)c1
237 FS(=O)(=O)c1ccc(CC(=O)NCCN2CCc3ccccc3C2)cc1
238 FS(=O)(=O)c1ccc(CC(=O)N2CCC(CC2)c2ccccc2)cc1
239 FS(=O)(=O)c1ccc(CC(=O)NCCc2nc3ccccc3[nH]2)cc1
240 CS(=O)(=O)N1CCOC(CNC(=O)Cc2ccc(cc2)S(F)(=O)=O)C1
241 CN(C)C(=O)CN1CCN(CC1)C(=O)Cc1ccc(cc1)S(F)(=O)=O
242 CN(CC(=O)N1CCC(O)CC1)C(=O)Cc1ccc(cc1)S(F)(=O)=O
243 CC(C)(C)OC(=O)N1CCN(CC1)c1ccc(CNC(=O)Cc2ccc(cc2)S(F)(=O)=O)cc1
244 FS(=O)(=O)c1ccc(CC(=O)NCc2ccc(cc2)N2CCCCC2)cc1

245 CC(=O)Nc1ccc(CCNC(=O)Cc2ccc(cc2)S(F)(=O)=O)cc1
246 CN1CCN(CC1c1ncc[nH]1)C(=O)Cc1ccc(cc1)S(F)(=O)=O
247 FS(=O)(=O)c1ccc(CC(=O)N2CCCC2COCc2ccccc2)cc1
248 CN1CCN(CC1)C(=O)CCNC(=O)Cc1ccc(cc1)S(F)(=O)=O
249 CC(C)(C)OC(=O)N1CCCn2nnc(CNC(=O)Cc3ccc(cc3)S(F)(=O)=O)c2C1
250 FS(=O)(=O)c1ccc(CC(=O)N2CCC(CC2)N2CCOCC2)cc1
251 FS(=O)(=O)c1ccc(CC(=O)N2CCC(C2)n2cnc3CCCCc23)cc1
252 FS(=O)(=O)c1ccc(CC(=O)NCc2ccccc2N2CCOCC2)cc1
253 CNC(=O)[C@H](C)N1CC[C@H](NC(=O)Cc2ccc(cc2)S(F)(=O)=O)C1=O
254 Cc1noc(COC2CCCN(C2)C(=O)Cc2ccc(cc2)S(F)(=O)=O)n1
255 CC1(CCCCC1)NC(=O)CNC(=O)Cc1ccc(cc1)S(F)(=O)=O
256 Cn1nc(cc1O)C1CCN(CC1)C(=O)Cc1ccc(cc1)S(F)(=O)=O
257 FS(=O)(=O)c1ccc(CC(=O)NCc2cccc(Oc3ccccn3)c2)cc1
258 CS(=O)(=O)c1cccc(CNC(=O)Cc2ccc(cc2)S(F)(=O)=O)c1
259 FS(=O)(=O)c1ccc(CC(=O)N2CCC3(CCCC(=O)N3)CC2)cc1
260 FS(=O)(=O)c1ccc(CC(=O)N2CCN(C(=O)C2)c2ccccc2)cc1
261 COc1ccc(CN(C)C(=O)Cc2ccc(cc2)S(F)(=O)=O)cc1O
262 Oc1ccc(cc1)N1CCN(CC1)C(=O)Cc1ccc(cc1)S(F)(=O)=O
263 CC1CCC(CNC(=O)Cc2ccc(cc2)S(F)(=O)=O)(CC1)N(C)C
264 FS(=O)(=O)c1ccc(CC(=O)N2CCCC2c2nc3CCCCc3[nH]2)cc1
265 Cn1nc2CCN(CCn2c1=O)C(=O)Cc1ccc(cc1)S(F)(=O)=O
266 Cn1c(n[nH]c1=O)C1CCCN(C1)C(=O)Cc1ccc(cc1)S(F)(=O)=O
267 FS(=O)(=O)c1ccc(CC(=O)NCc2ccc(nc2)N2CCOCC2)cc1
268 CN(Cc1nn(C)c2ccccc12)C(=O)Cc1ccc(cc1)S(F)(=O)=O
269 CC(C)C(=O)N1CCC(CC1)NC(=O)Cc1ccc(cc1)S(F)(=O)=O
270 CN(C)C(=O)Nc1cccc(CNC(=O)Cc2ccc(cc2)S(F)(=O)=O)c1
271 FS(=O)(=O)c1ccc(CC(=O)N2CCCCCC2c2ccncc2)cc1
272 Cn1cnc2CN(CCc2c1=O)C(=O)Cc1ccc(cc1)S(F)(=O)=O
273 COc1cccc(c1)C(O)CNC(=O)Cc1ccc(cc1)S(F)(=O)=O
274 FS(=O)(=O)c1ccc(CC(=O)NCC(c2ccccc2)c2ccccc2)cc1
275 FS(=O)(=O)c1ccc(CC(=O)NC(c2ccccc2)c2ccccc2)cc1
276 CN(C(C)=O)c1ccccc1CNC(=O)Cc1ccc(cc1)S(F)(=O)=O
277 CC(C)(C)OC(=O)N1CCN(CC1)c1ccccc1CNC(=O)Cc1ccc(cc1)S(F)(=O)=O
278 CC(NC(=O)Cc1ccc(cc1)S(F)(=O)=O)c1ccc(cc1)S(C)(=O)=O
279 FS(=O)(=O)c1ccc(CC(=O)NCc2cccc(c2)-c2cccnc2)cc1
280 Cc1cccc(c1)C1CCCN1C(=O)Cc1ccc(cc1)S(F)(=O)=O
281 CC(=O)N(CC1CCCN1C(=O)Cc1ccc(cc1)S(F)(=O)=O)C1CC1
282 CN1CCOC(CN(CC(N)=O)C(=O)Cc2ccc(cc2)S(F)(=O)=O)C1
283 CC(C)(C)OC(=O)N1CCC(CC1)N(Cc1cccnc1)C(=O)Cc1ccc(cc1)S(F)(=O)=O
284 CC(=O)N1CCOC(CCNC(=O)Cc2ccc(cc2)S(F)(=O)=O)C1
285 FS(=O)(=O)c1ccc(CC(=O)N2CCCCC2c2ccccn2)cc1
286 CC(C)(C)OC(=O)N1CCCCC1C1CCCCN1C(=O)Cc1ccc(cc1)S(F)(=O)=O
287 Cc1ccc2[nH]c(CNC(=O)Cc3ccc(cc3)S(F)(=O)=O)nc2c1
288 FS(=O)(=O)c1ccc(CC(=O)NCCNS(=O)(=O)c2ccccc2)cc1
289 Cn1cc(cn1)C1CN(CCO1)C(=O)Cc1ccc(cc1)S(F)(=O)=O
290 NS(=O)(=O)c1ccc(CCNC(=O)Cc2ccc(cc2)S(F)(=O)=O)cc1
291 CC(C)(C)OC(=O)N1CCC(CC1)N(C1CCCCC1)C(=O)Cc1ccc(cc1)S(F)(=O)=O
292 OCCn1nccc1C1CCN(CC1)C(=O)Cc1ccc(cc1)S(F)(=O)=O

293 FS(=O)(=O)c1ccc(CC(=O)N2CCOC(Cn3cncn3)C2)cc1

294 FS(=O)(=O)c1ccc(CC(=O)N2CCN(C3CCCCC3)C(=O)C2)cc1

295 FS(=O)(=O)c1ccc(CC(=O)N[C@H]2CC[C@@H](CC2)Oc2ccccn2)cc1

296 FS(=O)(=O)c1ccc(CC(=O)N2CCN(Cc3cccnc3)CC2)cc1

297 CN(C)C(=O)c1cccc(CNC(=O)Cc2ccc(cc2)S(F)(=O)=O)c1

298 FS(=O)(=O)c1ccc(CC(=O)N2CCCC(Cn3cncn3)C2)cc1

299 FS(=O)(=O)c1ccc(CC(=O)NC(Cc2ccccc2)c2ccccc2)cc1

300 OCCn1nccc1C1CCCN(C1)C(=O)Cc1ccc(cc1)S(F)(=O)=O

301 Cc1cccc(C)c1OCCNC(=O)Cc1ccc(cc1)S(F)(=O)=O

302 FS(=O)(=O)c1ccc(CC(=O)N2CCOC(COc3ccccc3)C2)cc1

303 CC(=O)N1CCN(CC1)C(=O)Cc1ccc(cc1)S(F)(=O)=O

304 FS(=O)(=O)c1ccc(CC(=O)N2CCN(CC2)c2ccccc2)cc1

305 CN1CCC(CN2CCN(CC2)C(=O)Cc2ccc(cc2)S(F)(=O)=O)CC1

306 FS(=O)(=O)c1ccc(CC(=O)N2CCN(CC2)C(=O)N2CCCC2)cc1

307 CN(CC1CCc2nccn2C1)C(=O)Cc1ccc(cc1)S(F)(=O)=O

308 CC(C)(C)OC(=O)N1CCC(CC1)N(C1CCCC1)C(=O)Cc1ccc(cc1)S(F)(=O)=O

309 CC(C)C(=O)Nc1cccc(CNC(=O)Cc2ccc(cc2)S(F)(=O)=O)c1

310 NC(=O)C1CCN(CC1)C(=O)CNC(=O)Cc1ccc(cc1)S(F)(=O)=O

311 FS(=O)(=O)c1ccc(CC(=O)N2CCCCC2CCN2CCCC2)cc1

312 FS(=O)(=O)c1ccc(CC(=O)NC2(CN3CCOCC3)CCCC2)cc1

313 Cc1nc2ccccc2n1CCNC(=O)Cc1ccc(cc1)S(F)(=O)=O

314 FS(=O)(=O)c1ccc(CC(=O)NCCn2nnc3ccccc23)cc1

315 FS(=O)(=O)c1ccc(CC(=O)NCCNC(=O)c2ccncc2)cc1

316 COCCN(C1CCS(=O)(=O)C1)C(=O)Cc1ccc(cc1)S(F)(=O)=O

317 Cc1noc(n1)C1(O)CCN(C1)C(=O)Cc1ccc(cc1)S(F)(=O)=O

318 NC(=O)c1ccccc1OC1CN(C1)C(=O)Cc1ccc(cc1)S(F)(=O)=O

319 CCCN1CCC(CC1)N(C1CC1)C(=O)Cc1ccc(cc1)S(F)(=O)=O

320 FS(=O)(=O)c1ccc(CC(=O)N2CCC(CC2)c2ccncc2)cc1

321 FS(=O)(=O)c1ccc(CC(=O)N2CCOC(Cn3cccn3)C2)cc1

322 FS(=O)(=O)c1ccc(CC(=O)N2CCC(CC2)N2C(=O)CNC2=O)cc1

323 FS(=O)(=O)c1ccc(CC(=O)N2CCNC(=O)C2c2ccccc2)cc1

324 FS(=O)(=O)c1ccc(CC(=O)N2CCN(Cc3ccccn3)CC2)cc1

325 Cn1cc(cn1)C1CCN(CC1)C(=O)Cc1ccc(cc1)S(F)(=O)=O

326 FS(=O)(=O)c1ccc(CC(=O)NCCNC(=O)Nc2ccccc2)cc1

327 FS(=O)(=O)c1ccc(CC(=O)NCc2ccc(cc2)-n2ccnc2)cc1

328 Cn1ccnc1[C@H]1OCCC[C@@H]1NC(=O)Cc1ccc(cc1)S(F)(=O)=O

329 FS(=O)(=O)c1ccc(CC(=O)N2CCN(Cc3ccncc3)CC2)cc1

330 FS(=O)(=O)c1ccc(CC(=O)N2CCCC2Cc2cccnc2)cc1

331 FS(=O)(=O)c1ccc(CC(=O)NCCc2nc3ccccc3[nH]2)cc1

332 Cn1cnnc1C1CCN(CC1)C(=O)Cc1ccc(cc1)S(F)(=O)=O

333 FS(=O)(=O)c1ccc(CC(=O)NC2CCc3n[nH]c(=O)n3CC2)cc1

334 Cn1nc(cc1O)C1CN(CCO1)C(=O)Cc1ccc(cc1)S(F)(=O)=O

335 Cc1cnn(c1)C1CCCN(C1)C(=O)Cc1ccc(cc1)S(F)(=O)=O

336 FS(=O)(=O)c1ccc(CC(=O)N[C@H]2CC[C@@H](CC2)Oc2cnccn2)cc1

337 CN1C(=O)NC(=O)C11CCN(CC1)C(=O)Cc1ccc(cc1)S(F)(=O)=O

338 CS(=O)(=O)c1ccc(CNC(=O)Cc2ccc(cc2)S(F)(=O)=O)cc1

339 FS(=O)(=O)c1ccc(CC(=O)NC2CCCN(C2)c2ncc[nH]c2=O)cc1

340 CC(C)(C)OC(=O)NCCCC(=O)N1CCN(CC1)C(=O)Cc1ccc(cc1)S(F)(=O)=O

341 FS(=O)(=O)c1ccc(CC(=O)NCCn2ccnc2-c2ccccc2)cc1
342 COc1ncnc2CCN(Cc12)C(=O)Cc1ccc(cc1)S(F)(=O)=O
343 FS(=O)(=O)c1ccc(CC(=O)N2CCC(CC2)c2c[nH]c3ccccc23)cc1
344 FS(=O)(=O)c1ccc(CC(=O)N[C@H]2C[C@H](C2)c2cc(=O)[nH]cn2)cc1
345 CN1CCCC(C1)C1CCCN1C(=O)Cc1ccc(cc1)S(F)(=O)=O
346 FS(=O)(=O)c1ccc(CC(=O)NCC2CN(C(=O)C2)c2ccccc2)cc1
347 FS(=O)(=O)c1ccc(CC(=O)NCCCn2cnc3ccccc23)cc1
348 CC(O)c1cc2CN(CCn2n1)C(=O)Cc1ccc(cc1)S(F)(=O)=O
349 CC(C)(C)OC(=O)N1CC2(CCN(CC2)C(=O)Cc2ccc(cc2)S(F)(=O)=O)c2ccccc12
350 Cn1nccc1C(=O)NCCNC(=O)Cc1ccc(cc1)S(F)(=O)=O
351 CC(C)(C)c1cn2CC(CCc2n1)NC(=O)Cc1ccc(cc1)S(F)(=O)=O
352 CC(C)C1NC(=O)CN1C(=O)CNC(=O)Cc1ccc(cc1)S(F)(=O)=O

3.3. HTC-D2B protocol

To a Labcyte 384LDV plate containing 352 amine functionalized fragments (10 mM) in DMSO (5 µL per well) was added a stock solution of OSu **2a** (10 mM) and NEM (30 mM) in DMSO (5 µL per well). The plate was sealed, centrifuged (1 min, 1000 rpm), and allowed to sit at room temperature for 1 h. After the reaction (assumed product concentration: 5 mM), a Labcyte ECHO© 555 Liquid Handler was used to transfer the library of SFs to a Greiner 384 white low volume plate. Purified protein diluted from original stock into a buffer made up of HEPES (pH 7.5, 25 mM) and NaCl (50 mM) in distilled MS-grade water (15 µL per well) was subsequently added across the plate. The plate was sealed, centrifuged (1 min, 1000 rpm), incubated for 24 h, and subsequently analyzed by intact protein LC-MS.

An analogous protocol was also carried out using OSu **3a** for the initial screen against CAII, and the screening conditions for the panel of purified proteins were as follows:

Table S7. Screening conditions used for the HTC-D2B protocol against a range of purified proteins.

**Screen**	[Protein] / µM	Incubation Temperature / °C	Incubation Duration / h	[SF] / µM
CAII (with reactive moiety **3**)	0.5	20	24	20
CAII (with reactive moiety **2**)	0.5	20	24	20
KRAS4B^{G12D} (with reactive moiety **2**)	1	20	24	20
BCL6 (with reactive moiety **2**)	1	4	24	50

4. CAII – site(s) of binding studies

4.1. Displacement study with ethoxzolamide

The (resynthesized and purified) SF hits (**2b**–**e**) were plated into a Greiner 384 white low volume plate from a 10 mM source in DMSO. Ethoxzolamide (**4**) (supplied by Sigma Aldrich) was also added from a 10 mM source in DMSO, or DMSO only as a control. Purified CAII diluted from original stock into a buffer made up of HEPES (pH 7.5, 25 mM) and NaCl (50 mM) in distilled MS-grade water (10 µL per well) was subsequently added across the plate. Final concentrations: 0.5 µM protein; 50 µM SF; 50 µM ethoxzolamide. The plate was sealed, centrifuged (1 min, 1000 rpm), incubated at 20 °C for 24 h, and subsequently analyzed by intact protein LC-MS. Negligible modification was observed for **2b**–**e** when incubated with ethoxzolamide.

4.2. Identification of the site of covalent modification by tandem MS (CAII)

Methodology:

The (resynthesized and purified) SF hits (**2d** and **2e**) were plated into a Greiner 384 PP F-bottom plate from a 10 mM source in DMSO. Purified CAII diluted from original stock into a buffer made up of HEPES (pH 7.5, 25 mM) and NaCl (50 mM) in distilled MS-grade water (50 µL per well) was subsequently added across the plate. Final concentrations: 2 µM protein; 50 µM SF. The plate was sealed, centrifuged (1 min, 1000 rpm), incubated at 20 °C for 24 h, and 15 µL aliquots were subsequently removed and analyzed by intact protein LC-MS. The remaining samples (1 µg) were separated by SDS-PAGE to remove excess unbound compound. Gels were stained with colloidal Comassie InstantBlue and bands corresponding to CAII were excised, reduced with 10 mM TCEP (65 °C, 30 mins), and alkylated with 10 mM iodoacetamide (r.t., 30 mins, dark). Samples were digested with trypsin (Promega) 1:10 E:S (37 °C, 16 h) in 100 mM ammonium bicarbonate. After removal of the supernatant, peptides were extracted using acetonitrile. Combined supernatants were concentrated in a SpeedVac centrifuge and acidified (0.1% formic acid, 0.05% trifluoroacetic acid) prior to injection into the LC-MS/MS system.

LC-MS/MS analysis:

Digested samples were injected on an Easy-nLC 1000 UHPLC system (Thermo Scientific). The nanoLC was interfaced to a Q-Exactive Hybrid Quadrupole-Orbitrap Mass Spectrometer (Thermo Scientific). Tryptic peptides were loaded on a 2 cm × 75 µm Acclaim PepMap 100 C18 trapping column (Thermo Scientific) and separated on a 25 cm × 75 µm, 2 µm particles, PepMap C18, 2 µm particle column (Thermo Scientific) using a 50 min gradient of 2–38% acetonitrile, 0.2% formic acid and a flow of 300 nL/min. LC-MS/MS based peptide sequencing was performed by data dependent analysis (DDA). Full MS 400–1600 Da at 70,000 resolution, MS AGC target 1e6, MS Maximum IT 200 ms, followed by MS/MS top 10 HCD fragmentation, stepped normalized CE 23, 27 and 30 V, Isolation window 1.5 *m/z*, fixed first mass 145 *m/z*, 17,500 resolution, MS/MS AGC target 5e4 and MS/MS Maximum IT 200 ms.

Data analysis:

Uninterpreted tandem MS spectra were searched for peptide matches against the sequence for CAII using the Mascot (Version 2.6.0) software with a 5 ppm mass tolerance for peptide precursors and 20 mDa mass tolerance for fragment ions.[9] Raw files were searched using trypsin as the enzyme with up to 2 missed cleavages and the variable modifications carbamidomethylation on cysteine and oxidation on methionine were allowed. Masses corresponding to [SF−HF] were allowed as variable modification(s) on cysteine, histidine, lysine, tyrosine, serine, and threonine as well as the protein N-terminus. MS/MS spectra were manually validated and annotated.

4.3. Virtual docking of hit compounds

Virtual molecular docking of SF **2e** (or **2d**) was carried out in Molecular Operating Environment (MOE) (Version 2019.0101).[10] PDB file 3CAJ (human CAII complexed with ethoxzolamide) was used. The docking tool was used to map SF **2e** (or **2d**) in the Zn^{2+} binding site

using ethoxzolamide as a template. The methods used were Placement: Triangle Matcher; Score: London dG and Refinement: Rigid Receptor; Score: GBVI/WSA dG.

5. CAII – kinetic analyzes

5.1. Protocol for kinetic analyzes (CAII)

The (resynthesized and purified) SF hits (**2b**–**e**) were plated into seven Greiner 384 white low volume plate from a 10 mM source in DMSO. Differing volumes were added such that the final concentrations for each compound would be: 100, 50, 20, 10, or 5 µM after adding 15 µL of protein stock solution per well. Volumes of DMSO were made to be consistent for every well. Purified CAII diluted from original stock into a buffer made up of HEPES (pH 7.5, 25 mM) and NaCl (50 mM) in distilled MS-grade water (15 µL per well) was subsequently added across the plates. Final concentrations: 0.5 µM protein; 100, 50, 20, 10, or 5 µM SF. The plates were sealed, centrifuged (1 min, 1000 rpm), and the first six plates were immediately queued for analysis by intact protein LC-MS at a temperature of 20 °C with the final plate paused for a 24 h timepoint. The resulting modification yields were plotted in GraphPad Prism (Version 5.0.4) software against the time of sampling.[11] Non-linear regression analyzes were conducted using a 'one-phase association' model, with the y=0 value constrained to '0' and plateau value constrained to '100'. The observed rate constants (k_{obs}, value 'K' in GraphPad) were extracted for each compound at each concentration, then replotted against the corresponding compound concentration. Further non-linear regression analyzes were conducted using a 'Michaelis-Menten' model, from which k_{inact} values ('Vmax' in GraphPad) and K_I values ('Km') were extracted.

5.2. Protocol for iterative screen against CAII

Amine selection:

Amines which were close analogues of initial hit SFs **2b**–**e** were selected. Initial selection criteria were applied: GSK compounds having at least one sample with concentration 10 mM in DMSO or above and at least 50 µL sample size. Unstable compounds and compounds with other liabilities were removed using proprietary GSK filters. Further selection criteria were then applied: $150<M_W<350$, BioByte cLogP<9 and only 1 aliphatic amine group.[5] 96 Amine-functionalized fragments were selected from the GSK compound collection based on Tanimoto similarity to original hits, and these comprised 79 primary sulfonamides and 17 secondary/tertiary sulfonamides or aryl sulfones.

HTC-D2B iterative screen against CAII:

To a Labcyte 384LDV plate containing 96 amine functionalized fragments (10 mM) in DMSO (5 µL per well) was added a stock solution of OSu **2a** (10 mM) and NEM (30 mM) in DMSO (5 µL per well). The plate was sealed, centrifuged (1 min, 1000 rpm), and allowed to sit at room temperature for 1 h. After the reaction (assumed product concentration: 5 mM), a Labcyte ECHO© 555 Liquid Handler was used to transfer the library of SFs to a Greiner 384 white low volume plate. Purified BCL6 diluted from original stock into a buffer made up of HEPES (pH 7.5, 25 mM) and NaCl (50 mM) in distilled MS-grade water (15 µL per well) was subsequently added across the plate. Final concentrations: 0.5 µM protein; 20 µM SF. The plate was sealed, centrifuged (1 min, 1000 rpm), incubated at 20 °C for 24 h, and subsequently analyzed by intact protein LC-MS.

6. CAII – chemoproteomics

6.1. Single shot screen of chemoproteomic probes against CAII

The (synthesized and purified) active probes **2j**, and **2k**, as well as negative control **2l**, were plated into a Greiner 384 white low volume plate from a 10 mM source in DMSO. Purified CAII diluted from original stock into a buffer made up of HEPES (pH 7.5, 25 mM) and NaCl (50 mM) in distilled MS-grade water (15 µL per well) was subsequently added across the plate. Final concentrations: 1 µM protein; 50 µM SF. The plate was sealed, centrifuged (1 min, 1000 rpm), incubated at 4 °C for 24 h, and subsequently analyzed by intact protein LC-MS. Modification yields were measured as 78% for **2j** and 95% for **2k**. Negligible modification was observed for **2l**.

6.2. Chemoproteomics workflow

Acetylation of NeutrAvidin agarose slurry:

NeutrAvidin agarose slurry (10 mL) (Thermo Fisher Scientific, 29204) was centrifuged (2 min, 2000 rpm) prior to removal of the supernatant. The beads were washed three times with PBS. PBS (9 mL) and Sulfo-NHS-acetate (482 µL, 400 mM in DMSO) (Thermo Fisher Scientific, 26777) were added, the beads were incubated at room temperature for 30 min on a falcon tube roller, and then centrifuged (2 min, 2000 rpm). Supernatant was removed and the incubation step with freshly made NHS-acetate was repeated. The reaction was quenched by adding Tris (2 mL, 1 M, pH 7.5). The beads were washed once with PBS and twice with 20% EtOH. 20% EtOH (5 mL) was added, and the beads were stored at 4 °C.

Chemoproteomics workflow:

HEK293T cell line (female human origin, The Francis Crick Institute cell service) was used for this study. The cells were maintained at 37 °C with 5% CO_2 in DMEM media supplemented with 10% fetal bovine serum and 1% L-Glutamine–Penicillin–Streptomycin solution (200 mM L-glutamine, 10,000 U/mL penicillin and 10 mg/mL streptomycin).

HEK293T cells were treated in triplicate with the parent compounds **2d** and **2e** (40 µM final concentration) or DMSO vehicle for 1 h at 37 °C in serum free media followed by a 1 h treatment with the probes **2j**, **2k** and **2l** (10 µM final concentration) or DMSO vehicle. Media was removed and cells were washed with PBS. Cells were lysed in lysis buffer containing 0.1% SDS, 1% IGEPAL, 0.5% Na-deoxycholate, 150 mM NaCl, 50 mM HEPES pH 8.0, 1× EDTA-free protease inhibitor cocktail (1:100) and benzonase (1:1000). The lysates were clarified by centrifugation (5 min, 10,000 rpm, 4 °C). Protein concentration of each lysate was determined using a BCA assay (Thermo Fisher Scientific, 23227).

Each lysate (376 µL, concentrations adjusted to 2.7 µg/µL) was treated with premixed click chemistry mixture (24 µL, final concentrations of biotin-PEG3-azide (100 µM) (Sigma-Aldrich, 762024), $CuSO_4$ (1 mM), TCEP (1 mM), BTTAA (100 µM)) for 1 h. The click reaction was quenched by adding EDTA (8 µL, 500 mM, final concentration of 10 mM).

Proteins were precipitated using ice-cold acetone and the resulting pellets were washed twice with ice-cold 80% acetone. The air-dried pellets were dissolved in 0.2% SDS (400 µL) in HEPES (50 mM, pH 8.0) by vortexing and sonicating.

Samples were incubated with acetylated NeutrAvidin agarose slurry (100 µL) (pre-washed three times with 0.2% SDS in HEPES (50 mM, pH 8.0)) on a combinatorial microlute plate (Porvair, 240002) for 2 h. The plate was centrifuged (1 min, 700 g) to remove supernatants. The beads were washed three times with lysis buffer and 0.2% SDS in HEPES (50 mM, pH 8.0). The proteins were digested on-bead overnight at 37 °C with LysC (60 µL, 0.004 µg/µL) in HEPES (50 mM, pH 8.0). The supernatants were collected (1 min, 700 g) on a fresh plate and trypsin (30 µL, 0.006 µg/µL) in HEPES (50 mM, pH 8.0) was added to each sample. The samples were incubated for 4 h at 37 °C and acidified with 20% formic acid (10 µL).

The peptide samples were cleaned-up using C18 96-well plate (BioPureSPE Macro 96-Well, 100 mg PROTO C18, The Nest Group). The wells were conditioned with acetonitrile (300 µL) (centrifuged for 1 min at 50 g) and equilibrated twice with 0.1% TFA (300 µL) (centrifuged for 1 min at 150 g). 0.1% TFA (100 µL) was added to digest samples, and these were subsequently loaded on the plate (centrifuged for 1 min at 150 g). The samples were washed twice with 0.1% TFA (200 µL) (centrifuged for 1 min at 150 g). The plate was centrifuged once more at 200 g for 1 min. The peptides were eluted in two steps with 0.1% TFA (150 µL) in 50% acetonitrile

(centrifuged for 1 min at 200 g) into a collection plate. The plate was frozen, and the samples were dried in Labconco CentriVap Benchtop Vacuum Concentrator at 35 °C.

6.3. LC-MS/MS analysis

Peptides were redissolved in 0.1% formic acid in water and samples were loaded with iRT standard (Biognosys AG) onto Evotips (as prepared according to manufacturer's instructions, EV2001) followed by loading onto the Evosep One LC system in front of the Orbitrap Fusion Lumos (Thermo Fisher Scientific). The Evosep One was fitted with a 15 cm column (EV1113) and the predefined method for a 44 min run was employed. Data for all samples was acquired in Data Independent Acquisition mode (DIA). Data for one replicate of each condition was also acquired by Data Dependent Acquisition mode (DDA). DIA Lumos settings were as follows: Transfer capillary set to 300 °C and 2.2 kV applied to the nanospray needle (Evosep). MS1 data acquired in the Orbitrap with a resolution of 120k, max injection time of 20 ms, AGC target of 1e6, in positive ion mode, in profile mode, over the mass range 393–907 m/z. DIA segments over this mass range (20 m/z wide/1 Da overlap/27 in total) were acquired in the Orbitrap following fragmentation in the HCD cell (32%), with 30k resolution over the mass range 200–2000 m/z and with a max injection time of 54 ms (dynamic) and AGC target of 1e6. DDA data used the same source settings with the following MS method changes: MS1 resolution = 60k, charge state inclusion 2–6^{+}, MIPS mode (Peptide), dynamic exclusion of 15 s, intensity threshold of 5e4, DDA carried out with quadrupole isolation of 1.4 Da, HCD energy of 32%, MS2 acquired in the Orbitrap with 15k resolution, max injection time of 22 ms, AGC target of 1e6 in centroid mode.

6.4. Data analysis

The DDA and DIA data was searched using Pulsar search engine inside Spectronaut (Version 14.10.201222.47784).[12] A spectral library was first generated by searching the DDA and DIA data against the Homo Sapiens (August 2019), common contaminants and avidin fasta files.[12] BGS factory settings (default) were used, except no fixed modifications were selected. The library contained 12860 precursors that correspond to 10695 peptides from 2163 protein groups.

The DIA data was then searched against the generated library using BGS factory settings (default). The data was normalized using global average normalization strategy with automatic row selection. Run wise imputation (Q-value = 30%) was applied to the data set. The sample t-test was carried out in Spectronaut to assess differential abundances (probe/DMSO, probe/competition and probe/negative control).[12]

Data was exported and the following thresholds were used for statistical significance:

- log2 ratio≥0.58
- p-value≤0.05
- #unique peptides≥2

Data visualization of exported candidates table was carried out in GraphPad Prism (Version 5.0.4) software.[11]

6.5. Data availability

The raw mass spectrometry proteomics files and database search results will be deposited at the ProteomeXchange Consortium (http://proteomecentral.proteomexchange.org) via the PRIDE partner repository.[13]

7. BCL6 – site of binding studies and iterative screen

7.1. Identification of the site(s) of covalent modification by tandem MS (BCL6)

Methodology:

The (resynthesized and purified) SF hits (**2f**–**i**) were plated into a Greiner 384 PP F-bottom plate from a 10 mM source in DMSO. Purified BCL6 diluted from original stock into a buffer made up of HEPES (pH 7.5, 25 mM) and NaCl (50 mM) in distilled MS-grade water (50 µL per well) was subsequently added across the plate. Final concentrations: 2 µM protein; 100 µM SF. The plate was sealed, centrifuged (1 min, 1000 rpm), incubated at 4 °C for 24 h, and 15 µL aliquots were subsequently removed and analyzed by intact protein LC-MS. The remaining samples (1 µg) were separated by SDS-PAGE to remove excess unbound compound. Gels were stained with colloidal Comassie InstantBlue and bands corresponding to BCL6 were excised, reduced with 10 mM TCEP (65 °C, 30 mins), and alkylated with 10 mM iodoacetamide (r.t., 30 mins, dark). Samples were digested with trypsin (Promega) 1:10 E:S (37 °C, 16 h) in 100 mM ammonium bicarbonate. After removal of the supernatant, peptides were extracted using acetonitrile. Combined supernatants were concentrated in a SpeedVac centrifuge and acidified (0.1% formic acid, 0.05% trifluoroacetic acid) prior to injection into the LC-MS/MS system.

LC-MS/MS analysis:

Digested samples were injected on an Easy-nLC 1200 UHPLC system (Thermo Scientific). The nanoLC was interfaced to an Orbitrap Fusion Lumos Mass Spectrometer (Thermo Scientific). Tryptic peptides were loaded on a 2 cm × 75 µm Acclaim PepMap 100 C18 trapping column (Thermo Scientific) and separated on a 25 cm × 75 µm, 2 µm particles, PepMap C18, 2 µm particle column (Thermo Scientific) using a 50 min gradient of 2–38% acetonitrile, 0.2% formic acid and a flow of 300 nL/min. LC-MS/MS based peptide sequencing was performed by data dependent analysis (DDA). Full MS 350–1600 Da at 120,000 resolution, MS AGC target 1e6, MS Maximum IT 50 ms, followed by MS/MS using a 3 sec. cycle time, stepped collision energy mode at 25, 29 and 32 CE%, Isolation window 1.6 *m/z*, fixed first mass 145 *m/z*, 15,000 orbitrap resolution, MS/MS AGC target 1e5 and MS/MS Maximum IT 200 ms. Alternatively, samples were analyzed by Parallel Reaction Monitoring (PRM) targeting precursor ions relative to the modified peptides. Full MS 380–1500 Da at 120,000 resolution, MS AGC target 1e6, MS Maximum IT 50 ms, followed by PRM scans using a fixed collision energy of 30 CE%, Isolation window 1.2 *m/z*, scan range of 140–2000 *m/z*, 30,000 orbitrap resolution, MS/MS AGC target 1e5 and MS/MS Maximum IT 100 ms.

Data analysis:

Uninterpreted tandem MS spectra were searched for peptide matches against the sequence for BCL6 using the Mascot (Version 2.6.0) software with a 5 ppm mass tolerance for peptide precursors and 20 mDa mass tolerance for fragment ions.[9] Raw files were searched using trypsin as the enzyme with up to 2 missed cleavages and the variable modifications carbamidomethylation on cysteine and oxidation on methionine were allowed. Masses corresponding to [SF−HF] were allowed as variable modification(s) on cysteine, histidine, lysine, tyrosine, serine, and threonine as well as the protein N-terminus. MS/MS spectra were manually validated and annotated.

7.2. Protocol for iterative screen against BCL6

Amine selection:

Amines were selected to include those that had molecular similarity to initial hit SFs **2f**–**i**. The similarity searching was performed using a Pipeline Pilot (Version 20.1.0.2208) software protocol.[4] Initial selection criteria were applied: GSK compounds having at least one sample with concentration 10 mM in DMSO or above and at least 50 µL sample size. Unstable compounds and compounds with other liabilities were removed using proprietary GSK filters. Further selection criteria were then applied: $150<M_W<350$, BioByte cLogP<9 and only 1 aliphatic amine group.[5] Substructure filters were used to create a pool of available molecules. SmallWorld clustering was used with the original query compound(s) and the pooled compounds, and the compounds were tagged as to whether they were generated with BioDig molecule generator from the original query compound. A 352-membered library was selected from the remaining molecules. The selected amines (10 mM, 5 µL per well/screen) were ordered from GSK's solution stores in Labcyte 384LDV plates.

HTC-D2B iterative screen against BCL6:

To a Labcyte 384LDV plate containing the selected 352 amine functionalized fragments (10 mM) in DMSO (5 µL per well) was added a stock solution of OSu **2a** (10 mM) and NEM (30 mM) in DMSO (5 µL per well). The plate was sealed, centrifuged (1 min, 1000 rpm), and allowed to sit at room temperature for 1 h. After the reaction (assumed product concentration: 5 mM), a Labcyte ECHO© 555 Liquid Handler was used to transfer the library of SFs to a Greiner 384 white low volume plate. Purified BCL6 diluted from original stock into a buffer made up of HEPES (pH 7.5, 25 mM) and NaCl (50 mM) in distilled MS-grade water (15 µL per well) was subsequently added across the plate. Final concentrations: 1 µM protein; 50 µM SF. The plate was sealed, centrifuged (1 min, 1000 rpm), incubated at 4 °C for 24 h, and subsequently analyzed by intact protein LC-MS.

8. BCL6 – Further investigations into hits by structural and biophysical studies

8.1. Displacement study with GSK137

The (resynthesized and purified) SF hits (**2f–i**, **2p**, **2q**, **2s**, and **2t**) were plated into a Greiner 384 white low volume plate from a 10 mM source in DMSO, or DMSO-d_6 only as a control. GSK137 (**11**) was also added from a 10 mM source in DMSO. Purified BCL6 diluted from original stock into a buffer made up of HEPES (pH 7.5, 25 mM) and NaCl (50 mM) in distilled MS-grade water (15 µL per well) was subsequently added across the plate. Final concentrations: 1 µM protein; 50 µM SF; 50 µM GSK137. The plate was sealed, centrifuged (1 min, 1000 rpm), incubated at 20 °C for 24 h, and subsequently analyzed by intact protein LC-MS. This was performed in duplicate and average covalent modification yields were used for analysis.

Table S8. Quantitative output from intact LC-MS analysis of BCL6 BTB/POZ domain reacted with SFs after incubation of DMSO-d_6 or GSK137. Extent of displacement measured as (DModification/Modification(DMSO-d_6 treatment)).

Compound	Modification (GSK137 treatment) (%)	Modification (DMSO-d_6 treatment) (%)	DModification (%)	Displacement Ratio	Displacement (%)
**2f**	3.8	17.6	13.8	0.78	78
**2g**	4.8	25.0	20.2	0.81	81
**2h**	1.6	10.3	8.7	0.84	84
**2i**	1.6	19.7	18.1	0.92	92
**2p**	2.2	24.2	22	0.91	91
**2q**	3.0	14.4	11.4	0.79	79
**2s**	2.8	78.9	76.1	0.96	96
**2t**	0.4	10.4	10.0	0.96	96

8.2. Conventional differential scanning fluorimetry (DSF)

BCL6-SF covalent complex preparation:

SFs **2f–i**, **2p**, **2q**, **2s**, and **2t** in DMSO-d_6 (200 mM stock) or pure DMSO-d_6 were added to crystallizable BCL6 BTB/POZ domain (10 µM) in 20 mM Tris pH 8.5 and 200 mM NaCl. The reactions were incubated at 20 °C for 6 h (*meta*-substituted benzamide fragments) or 24 h (azetidinyl/piperidinyl fragments). Final concentrations: 10 µM protein, 500 µM SF, DMSO 0.25% *v/v* in 200 µL. Unreacted compound and DMSO-d_6 were removed by buffer-exchanging protein solutions into 20 mM Tris pH 8.5, 200 mM NaCl and 5% *v/v* glycerol inside Vivaspin 500 PES Centrifugal Concentration units. The protein was concentrated to 0.6–1.3 mg/mL within the units, according to UV-vis absorption spectrophotometry via a Nanodrop ND-1000 instrument. Protein was flash frozen using a dry ice/ethanol bath prior to storage at −80 °C.

Differential scanning fluorimetry assay:

GSK137 (40 mM stock) or pure DMSO-d_6 were added to crystallizable BCL6 BTB/POZ domain (11 µM) in 20 mM Tris pH 8.5, 200 mM NaCl and 5% *v/v* glycerol to give solutions containing: 11 µM protein, DMSO 0.63% *v/v* and 222 µM GSK137 (where applicable). Concentrated protein-SF complexes were also thawed and diluted to 11 µM in 20 mM Tris pH 8.5, 150 mM NaCl and 5% *v/v* glycerol.

SYPRO Orange dye (Invitrogen S6650) in extra dilution buffer (50×, *eq*. 1% *v/v* DMSO) was added to give assay-ready solutions with final concentrations: 10 µM protein, SYPRO 5×, DMSO 0.1% *v/v* in volume 80 µL for the protein-SF covalent complexes or 10 µM protein, SYPRO 5×, DMSO 0.6% *v/v* in volume 100 µL with and without 200 µM GSK137.

Each solution was aliquoted (3 × 20 µL) into wells of an opaque 384-well PCR microplate (Axygen PCR-384-LC480-W) and coated with silicone oil DC 200 (5 µL; Fluka Chemicals 85413). The microplate was centrifuged (1 min, 200 g) prior to insertion into a Fluodia T70 spectrophotometer (Photal Instruments). The fluorescence intensity was detected (emission wavelength: 486 nm; excitation wavelength: 610 nm) as the plate instrument interior was heated from 27–75 °C using a temperature stepping mode (waiting time before measurement: 60 s; fluorescence measurement interval: 45 s). The approximate temperature gradient was 0.5 °C min^{-1}.

The fluorescence intensity data from the individual replicates of each sample type were normalized and identically truncated within GraphPad Prism 5.0.4.[11] These values were subsequently fitted to equation (4) corresponding to a Boltzmann sigmoid in order to obtain the melting temperature (“*V50*”; T_m) of the protein:

$$Y=BOTTOM+((TOP+BOTTOM)/(1+exp((V50–X)/SLOPE))) \quad (4)$$

The three obtained T_m values were averaged to give a final T_m value in each case. The normalized fluorescence data for the three replicates of each sample were averaged to yield a single dataset for each case, which were then plotted in GraphPad Prism 5.0.4 to give the plots shown in the manuscript.[11]

Table S9. Thermal melting temperatures of unmodified and covalently modified BCL6 BTB/POZ domain as determined by differential scanning fluorimetry measurements. The arithmetic mean and standard deviation from three replicates are shown.

Hit Chemotype	Protein Species	T_m / °C	ΔT_m / °C
--	BCL6 only	58.7 ± 0.6	--
	BCL6 + DMSO-d_6	58.1 ± 1.2	−0.5
--	BCL6 + GSK137	>72	>12.3
meta-Substituted Benzamide Fragments	BCL6 + **2f**	66.6 ± 0.3	+7.9
	BCL6 + **2g**	70.4 ± 0.3	+11.7
	BCL6 + **2s**	66.8 ± 0.2	+8.1
Azetidinyl Fragments	BCL6 + **2h**	55.5 ± 0.1	−3.2
	BCL6 + **2i**	59.9 ± 0.4	+1.2
Piperidinyl Fragments	BCL6 + **2p**	58.0 ± 0.2	−0.7
	BCL6 + **2q**	55.5 ± 0.1	−3.2
	BCL6 + **2t**	57.3 ± 0.1	−1.4

8.3. Protocol for kinetic analyzes (BCL6)

The (resynthesized and purified) SF hits (**2f**–**i**) were plated into seven Greiner 384 white low volume plate from a 10 mM source in DMSO. Differing volumes were added such that the final concentrations for each compound would be: 100, 50, 20, 10, or 5 µM after adding 15 µL of protein stock solution per well. Volumes of DMSO were made to be consistent for every well. Purified BCL6 diluted from original stock into a buffer made up of HEPES (pH 7.5, 25 mM) and NaCl (50 mM) in distilled MS-grade water (15 µL per well) was subsequently added across the plates. Final concentrations: 0.5 µM protein; 100, 50, 20, 10, or 5 µM SF. The plates were sealed, centrifuged (1 min, 1000 rpm), and the first six plates were immediately queued for analysis by intact protein LC-MS at a temperature of 4 °C with the final plate paused for a 24 h timepoint. The resulting modification yields were plotted in GraphPad Prism (Version 5.0.4) software against the time of sampling.[11] Non-linear regression analyzes were conducted using a ‘one-phase association’ model, with the y=0 value constrained to ‘0’. The observed rate constants (k_{obs}, value ‘K’ in GraphPad) were extracted for each compound at each concentration, then replotted against the corresponding compound concentration. Further non-linear regression analyzes were conducted using a ‘Michaelis-Menten’ model, from which k_{inact} values (‘Vmax’ in GraphPad) and K_I values (‘Km’) were extracted.

8.4. Protocol for crystallography

Methodology

BCL6 BTB domain (BCL6$^{5–129(C8Q,C67R,C84N)-FLAG}$, 10 µM) was incubated with each compound (500 µM, *eq*. 0.25% *v*/*v* DMSO) in 20 mM Tris pH 8.5 and 150 mM NaCl (2 mL) at 20 °C for 6 h. To remove unreacted compound, the protein-fragment complex was buffer exchanged into 20 mM Tris pH 8.5, 150 mM NaCl and 5% *v*/*v* glycerol by centrifugation. The protein-fragment complex was concentrated to >6 mg/mL, as determined by spectrophotometry. Sitting drop crystallization was performed at 20 °C by combining the protein-

fragment complex solution, precipitant solution (11% or 12% *w/v* polyethylene glycol (PEG) 3350, 16.8% *v/v* glycerol and 0.1 M bis-tris propane pH 7.2–7.3) and apo BCL6 seeds (in 17% *w/v* PEG 3350, 20% *v/v* glycerol and 0.1 M bis-tris propane pH 7.3) in a 10:8:2 ratio, respectively. Crystals were typically obtained within a few days. Precipitant solution was added to the crystallization drops to provide cryoprotection. Single crystals were directly flash frozen in liquid nitrogen from the drops for diffraction data collection at 100 K on the ID-30A beamline at the European Synchrotron Radiation Facility. Datasets were processed using XDS and scaled using aimless or STARANISO within AutoPROC.[14–17] In all instances, including for twinned data the correct symmetry was established to be $P3_221$. The structures were solved using an in-house search model with one protein molecule in the asymmetric unit of the $P3_221$ space group. Difference density was found near Tyr57 which could be modeled as the covalent ligand. Whilst the covalent bond was clear in all instances, the density for the rest of the ligand was variable in quality between the structures. The ligand was initially built with Grade.[18] The structure was iteratively modeled in COOT and refined using REFMAC, with appropriate TWIN refinement as required.[19–21] Electron density maps were created in CCP4MG within the CCP4 suite, and further structural figures in the manuscript were prepared in MOE (Version 2019.0101).[10,20]

Data deposition: The atomic coordinates and structure factors will be deposited in the Protein Data Bank, www.rcsb.org.

Table S10. Data collection and refinement statistics. Values in parentheses are for the highest resolution shell.

	**BCL6 BTB / SF 2f**	**BCL6 BTB / SF 2s**
**Data collection**		
**Space group**	$P3_221$	$P3_221$
**Cell dimensions**		
***a*, *b*, *c* (Å)**	48.859, 48.859, 123.878	49.398, 49.398, 124.630
***α*, *β*, *γ* (°)**	90.000, 90.000, 120.000	90.000, 90.000, 120.000
**Resolution (Å)**	43.32-1.61 (1.77-1.61)	42.78-1.82 (1.85-1.82)
R_{merge}	0.113 (1.971)	0.122 (1.480)
CC (1/2)	0.997 (0.716)	0.997 (0.657)
$I/\sigma(I)$	14.5 (1.6)	16.0 (2.1)
**Completeness (%)**	66.4 (13.7)	93.7 (100.0)
**Completeness ellipsoidal (%)**	87.8 (72.4)	/
**Redundancy**	18.8 (18.6)	18.7 (19.2)
**Refinement**		
**Resolution (Å)**	43.32-1.61	42.78-1.82
**No. reflections**	286715 (14142)	291004 (15815)
**No. uniq. reflections**	15242 (762)	15586 (822)
R_{work} / R_{free}	0.180/0.214	0.152/0.213
**No. atoms**	1291	1300
**Protein**	1117	1107
**Ligand/ion**	26	26
**Water**	128	167
**B-factors**		
**Protein**	40.359	40.057
**Ligand/ion**	80.682	56.638
**Water**	52.743	48.810
**R.m.s deviations**		
**Bond lengths (Å)**	0.004	0.002
**Bond angles (º)**	1.237	1.164
**TWIN fraction**	/	0.496/0.504

Ligand density:

The BCL6 BTB monomer is shown in a blue ribbon representation. Compounds are shown in ball-and-stick representation with green carbon atoms.

BCL6 BTB domain + compound SF **2f**<br>$2F_o$-F_c map in the active site contoured at +1.0σ (blue); +0.7σ (cyan)	BCL6 BTB domain + compound SF **2s**<br>F_o-F_c map in the active site contoured at +1.0σ (blue)
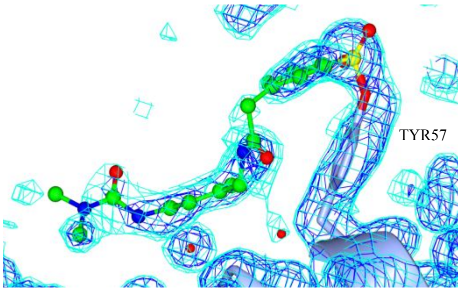<br>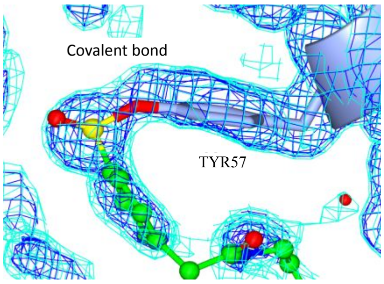	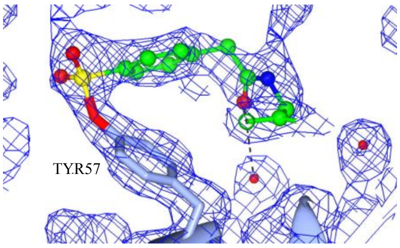<br>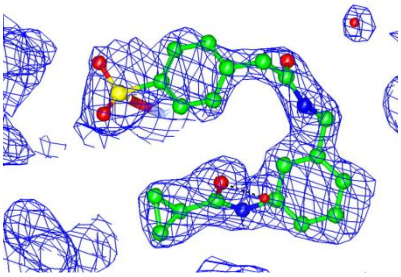
BCL6 BTB domain + compound SF **2f**<br>F_o-F_c map in the active site contoured at ±3.0σ (blue/red); +2.0σ (cyan)	-
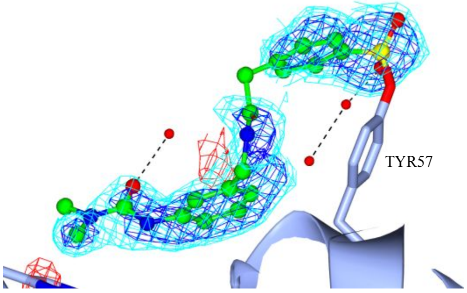<br>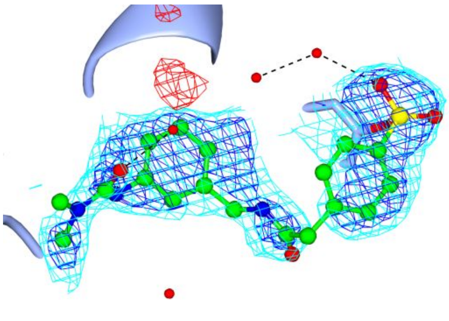	-

9. Compounds

Sulfur(VI) fluoride carboxylic acid precursors were supplied by Enamine.

Amine-functionalized fragments were obtained from the GSK compound collection.

Compounds listed in the order in which they were discussed in the manuscript:

2,5-Dioxopyrrolidin-1-yl 3-(fluorosulfonyl)benzoate, **1a**

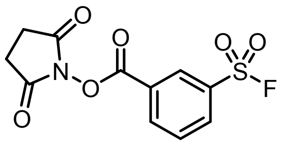

To a solution of 3-(fluorosulfonyl)benzoic acid (200 mg, 0.98 mmol) in acetonitrile (8.0 mL) was added DMAP (132 mg, 1.1 mmol) and TSTU (324 mg, 1.1 mmol). The solution was stirred for 15 mins at room temperature. Deionized water (20 mL) was added, and the aqueous phase was extracted with EtOAc (3 × 20 mL). The combined organic extracts were dried (hydrophobic frit) and concentrated *in vacuo* to afford a colorless gum. The crude product was purified by FCC (silica, 0–100% EtOAc:cyclohexane) to yield the title compound (171 mg, 0.57 mmol, 58%) as a white solid. **LC-MS** (formic acid modifier): t_r = 0.90 mins, 98% by UV. **^{1}H NMR** (400 MHz, DMSO-d_6): δ 8.66–8.63 (1H, m, 1 × ArC<u>H</u>), 8.63–8.61 (1H, m, 1 × ArC<u>H</u>), 8.61–8.59 (1H, m, 1 × ArC<u>H</u>), 8.12–8.07 (1H, m, 1 × ArC<u>H</u>), 2.94 (4H, s, 2 × C<u>H</u>$_2$). **^{13}C NMR** (101 MHz, DMSO-d_6): δ 170.4 (2C), 160.6 (1C), 137.9 (1C), 135.2 (1C), 133.3 (1C), 132.7 (1C), 129.8 (1C), 126.9 (1C), 26.1 (2C). **^{19}F NMR** (376 MHz, DMSO-d_6): δ 66.4 (1F, s). **IR** ν_{max} (cm^{-1}): 1773, 1733, 1600.

2,5-Dioxopyrrolidin-1-yl 2-(4-(fluorosulfonyl)phenyl)acetate, **2a**

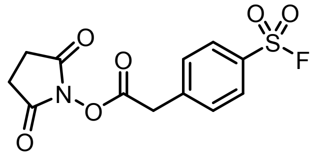

To a solution of 2-(4-(fluorosulfonyl)phenyl)acetic acid (150 mg, 0.69 mmol) in acetonitrile (6.5 mL) was added DMAP (92 mg, 0.76 mmol) and TSTU (228 mg, 0.76 mmol). The solution was stirred for 15 mins at room temperature. Deionized water (20 mL) was added, and the aqueous phase was extracted with EtOAc (3 × 20 mL). The combined organic extracts were dried (hydrophobic frit) and concentrated *in vacuo* to afford a colorless gum. The crude product was purified by FCC (silica, 0–100% EtOAc:cyclohexane). The isolated material was subsequently re-purified by FCC (silica, 30–100% EtOAc:cyclohexane) to yield the title compound (100 mg, 0.32 mmol, 46%) as a white solid. **LC-MS** (formic acid modifier): t_r = 0.94 mins, 96% by UV. **^{1}H NMR** (400 MHz, DMSO-d_6): δ 8.18 (2H, d, *J* = 8.4 Hz, 2 × ArC<u>H</u>), 7.79 (2H, d, *J* = 8.4 Hz, 2 × ArC<u>H</u>), 4.43 (2H, s, 1 × C<u>H</u>$_2$), 2.83 (4H, s, 2 × C<u>H</u>$_2$). **^{13}C NMR** (101 MHz, DMSO-d_6): δ 170.5 (2C), 167.1 (1C), 142.2 (1C), 132.0, (2C), 131.1 (1C), 129.2 (2C), 36.8 (1C), 25.9 (2C). **^{19}F NMR** (376 MHz, DMSO-d_6): δ 66.4 (1F, s). **HRMS** (ESI, positive ion mode): *m/z* for $[C_{12}H_{10}FNO_6S+Na]^+$ = 338.0121, found 338.0107. **IR** ν_{max} (cm^{-1}): 1815, 1780, 1727, 1596.

2,5-Dioxopyrrolidin-1-yl 1-(fluorosulfonyl)azetidine-3-carboxylate, **3a**

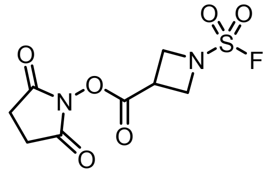

To a solution of 1-(fluorosulfonyl)azetidine-3-carboxylic acid (200 mg, 1.1 mmol) in acetonitrile (8.0 mL) was added DMAP (147 mg, 1.2 mmol) and TSTU (362 mg, 1.2 mmol). The solution was stirred for 15 mins at room temperature. Deionized water (20 mL) was added, and the aqueous phase was extracted with EtOAc (3 × 20 mL). The combined organic extracts were dried (hydrophobic frit) and concentrated *in vacuo* to afford a colorless gum. The crude product was purified by FCC (silica, 0–100% EtOAc:cyclohexane). To the isolated material was added saturated sodium bicarbonate solution (20 mL) and the aqueous phase was extracted with DCM (3 × 20 mL). The combined organic extracts were dried (hydrophobic frit) and concentrated *in vacuo* to yield the title compound (123 mg, 0.44 mmol, 40%) as a white solid. **LC-MS** (formic acid modifier): t_r = 0.76 mins. **^{1}H NMR** (400 MHz, DMSO-d_6): δ 4.63 (2H, ddd, *J* = 8.9, 6.0, 2.4 Hz, 2 × C<u>H</u>), 4.30 (2H, ddd, *J* = 8.9, 6.0, 2.4 Hz, 2 × C<u>H</u>), 4.19–4.15 (1H, m, 1 × C<u>H</u>), 2.86 (4H, s, 2 × C<u>H</u>$_2$). **^{13}C NMR** (101 MHz, DMSO-d_6): δ 170.4 (1C), 168.1 (2C), 55.1 (2C), 30.2 (1C), 26.0 (2C). **^{19}F NMR** (376 MHz, DMSO-d_6): δ 31.2 (1F, s). **IR** ν_{max} (cm^{-1}): 1813, 1779, 1726, 1417.

4-(2-Oxo-2-((1-(3-sulfamoylphenyl)ethyl)amino)ethyl)benzenesulfonyl fluoride, **2b**

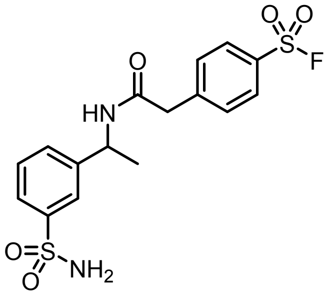

2-(4-(Fluorosulfonyl)phenyl)acetic acid (60 mg, 0.28 mmol), 3-(1-aminoethyl)benzenesulfonamide (72 mg, 0.36 mmol), and HATU (209 mg, 0.55 mmol) were dissolved in DMF (1.0 mL). To this solution was added DIPEA (0.062 mL, 0.36 mmol). The solution was stirred for 30 mins at room temperature. The crude reaction mixture was directly purified by MDAP (formic acid modifier). The solvent was dried under a stream of nitrogen to yield the title compound (9 mg, 0.022 mmol, 8%) as a white solid. **LC-MS** (formic acid modifier): t_r = 0.85 mins, >99% by UV, $[M+H]^+$ found 400.9. **^{1}H NMR** (400 MHz, DMSO-d_6): δ 8.80 (1H, d, *J* = 7.9 Hz, 1 × NH), 8.07 (2H, d, *J* = 8.4 Hz, 2 × ArCH), 7.81–7.77 (1H, m, 1 × ArCH), 7.74–7.69 (1H, m, 1 × ArCH), 7.68–7.64 (2H, d, *J* = 8.4 Hz, 2 × ArCH), 7.54–7.52 (1H, m, 1 × ArCH), 7.52–7.50 (1H, m, 1 × ArCH), 7.34 (2H, s, 1 × NH_2), 5.01–4.92 (1H, m, 1 × CH), 3.70 (2H, s, 1 × CH_2), 1.40 (3H, d, *J* = 6.9 Hz, 1 × CH_3). **^{13}C NMR** (101 MHz, DMSO-d_6): δ 168.5 (1C), 146.3 (1C), 146.0 (1C), 144.7 (1C), 131.5 (2C), 130.1 (1C), 129.9 (1C), 129.4 (1C), 128.8 (2C), 124.6 (1C), 123.4 (1C), 48.5 (1C), 42.4 (1C), 22.7 (1C). **^{19}F NMR** (376 MHz, DMSO-d_6): δ 66.6 (1F, s). **HRMS** (ESI, positive ion mode): *m/z* for $[C_{16}H_{17}FN_2O_5S_2+H]^+$ = 401.0641, found 401.0634. **IR** ν_{max} (cm^{-1}): 3330, 3254, 1637, 1541, 1416.

4-(2-Oxo-2-((3-(sulfamoylmethyl)benzyl)amino)ethyl)benzenesulfonyl fluoride, **2c**

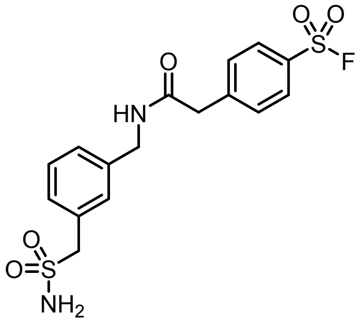

This compound was supplied by Enamine.

4-(2-Oxo-2-((3-sulfamoylbenzyl)amino)ethyl)benzenesulfonyl fluoride, **2d**

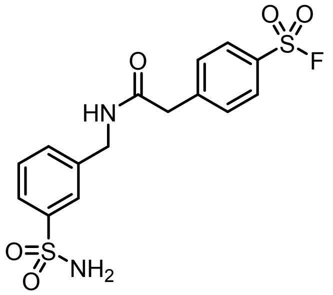

2-(4-(Fluorosulfonyl)phenyl)acetic acid (60 mg, 0.26 mmol), 3-(aminomethyl)benzenesulfonamide (67 mg, 0.36 mmol), and HATU (178 mg, 0.47 mmol) were dissolved in DMF (2.5 mL). To this solution was added DIPEA (72 µL, 0.41 mmol). The solution was stirred for 30 mins at room temperature. The crude reaction mixture was concentrated under a stream of nitrogen and subsequently purified by MDAP (formic acid modifier). The solvent was dried under a stream of nitrogen to yield the title compound (53 mg, 0.14 mmol, 50%) as a white solid. **LC-MS** (formic acid modifier): t_r = 0.83 mins, >99% by UV, $[M+H]^+$ found 387.2. **^{1}H NMR** (400 MHz, DMSO-d_6): δ 8.80 (1H, t, *J* = 5.7 Hz, 1 × NH), 8.11–8.08 (2H, m, 2 × ArCH), 7.75–7.73 (2H, m, 2 × ArCH), 7.72–7.70 (1H, m, 1 × ArCH), 7.69–7.67 (1H, m, 1 × ArCH), 7.54–7.51 (1H, m, 1 × ArCH), 7.49–7.45 (1H, m, 1 × ArCH), 7.35 (2H, s, 1 × NH_2), 4.37 (2H, d, *J* = 5.7 Hz, 1 × CH_2), 3.74 (2H, s, 1 × CH_2). **^{13}C NMR** (101 MHz, DMSO-d_6): δ 169.4 (1C), 146.2 (1C), 144.8 (1C), 140.8 (1C), 132.0 (1C), 131.5 (2C), 131.0 (1C), 129.4 (1C), 128.9 (1C), 128.8 (1C), 124.7 (2C), 42.5 (1C), 42.4 (1C). **^{19}F NMR** (376 MHz, DMSO-d_6): δ 66.6 (1F, s). **HRMS** (ESI, positive ion mode): *m/z* for $[C_{15}H_{15}FN_2O_5S_2+H]^+$ = 387.0485, found 387.0482. **IR** ν_{max} (cm^{-1}): 3332, 3263, 1646, 1546, 1413.

4-(2-Oxo-2-((4-sulfamoylphenethyl)amino)ethyl)benzenesulfonyl fluoride, **2e**

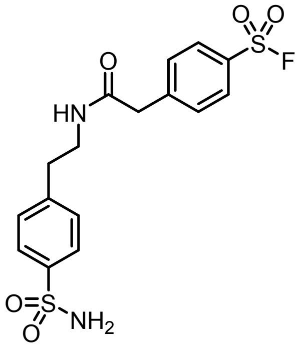

2-(4-(Fluorosulfonyl)phenyl)acetic acid (35 mg, 0.16 mmol), 4-(2-aminoethyl)benzenesulfonamide (42 mg, 0.21 mmol), and HATU (104 mg, 0.27 mmol) were dissolved in DMF (1.0 mL). To this solution was added DIPEA (42 µL, 0.24 mmol). The solution was stirred for 30 mins at room temperature. The crude reaction mixture was directly purified by MDAP (formic acid modifier). The solvent was dried under a stream of nitrogen to yield the title compound (29 mg, 0.071 mmol, 44%) as a white solid. **LC-MS** (formic acid modifier): t_r = 0.82 mins, 94% by UV, $[M+H]^+$ found 401.2. **^{1}H NMR** (400 MHz, DMSO-d_6): δ 8.27 (1H, t, *J* = 5.7 Hz, 1 × NH), 8.08 (2H, d, *J* = 7.9 Hz, 2 × ArCH), 7.74 (2H, d, *J* = 7.9 Hz, 2 × ArCH), 7.61 (2H, d, *J* = 8.4 Hz, 2 × ArCH), 7.38 (2H, d, *J* = 8.4 Hz, 2 × ArCH), 7.29 (2H, s, 1 × NH_2), 3.61 (2H, s, 1 × CH_2), 3.38–3.30 (2H, m, 1 × CH_2), 2.80 (2H, t, *J* = 6.9 Hz , 1 × CH_2). **^{13}C NMR** (101 MHz, DMSO-d_6): δ 169.2 (1C), 146.4 (1C), 144.0 (1C), 142.6 (1C), 131.4 (2C), 130.1 (1C), 129.6 (2C), 128.8 (2C), 126.1 (2C), 42.5 (1C), 40.4 (1C), 35.2 (1C). **^{19}F NMR** (376 MHz, DMSO-d_6): δ 66.6 (1F, s). **HRMS** (ESI, positive ion mode): *m/z* for $[C_{16}H_{17}FN_2O_5S_2+H]^+$ = 401.0641, found 401.0642. **IR** ν_{max} (cm^{-1}): 3330, 3224, 1637, 1541, 1416.

3-((Prop-2-yn-1-ylamino)methyl)benzenesulfonamide, **8**

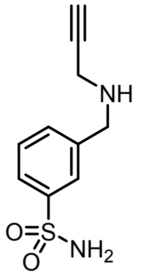

To a solution of 3-(aminomethyl)benzenesulfonamide (525 mg, 2.82 mmol) in DMF (25 mL) was added potassium carbonate (818 mg, 5.92 mmol) and propargyl bromide (80 wt. % in toluene) (440 mg, 2.96 mmol). The solution was stirred for 16 h at room temperature. Aqueous sodium bicarbonate solution (100 mL) was added, and the aqueous phase was extracted with EtOAc (3 × 100 mL). The combined organic extracts were dried (hydrophobic frit) and concentrated *in vacuo* to afford a yellow solid. The crude product was purified by FCC (silica, 0–100% EtOAc(+1% triethylamine):cyclohexane). The isolated material was subsequently re-purified by MDAP (HpH modifier). The solvent was dried under a stream of nitrogen to yield the title compound (18 mg, 0.080 mmol, 3%) as an orange gum. **LC-MS** (HpH modifier): t_r = 0.58 mins, >99% by UV, $[M+H]^+$ found 224.9. **^{1}H NMR** (400 MHz, DMSO-d_6): δ 7.82–7.80 (1H, m, 1 × ArCH), 7.71–7.69 (1H, m, 1 × ArCH), 7.55–7.53 (1H, m, 1 × ArCH), 7.52–7.49 (1H, m, 1 × ArCH), 7.31 (2H, s, 1 × NH_2), 3.82 (2H, s, 1 × CH_2), 3.31–3.30 (2H, m, 1 × CH_2), 3.10 (1H, t, *J* = 2.4 Hz, 1 × CH). **^{13}C NMR** (101 MHz, DMSO-d_6): δ 144.6 (1C), 141.8 (1C), 131.8 (1C), 129.2 (1C), 125.5 (1C), 124.5 (1C), 83.1 (1C), 74.4 (1C), 51.3 (1C), 37.2 (1C). **HRMS** (ESI, positive ion mode): *m/z* for $[C_{10}H_{12}N_2O_2S+H]^+$ = 225.0698, found 225.0702. **IR** ν_{max} (cm^{-1}): 3295, 3270, 1660, 1305.

4-(2-(Prop-2-yn-1-ylamino)ethyl)benzenesulfonamide, **9**

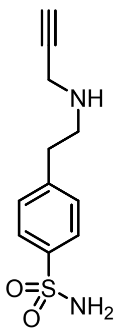

To a solution of 4-(2-aminoethyl)benzenesulfonamide (1000 mg, 5.0 mmol) in DMF (50 mL) was added potassium carbonate (1450 mg, 10.5 mmol) and propargyl bromide (80 wt. % in toluene) (780 mg, 5.2 mmol). The solution was stirred for 16 h at room temperature. Aqueous sodium bicarbonate solution (100 mL) was added, and the aqueous phase was extracted with EtOAc (3 × 100 mL). The combined organic extracts were dried (hydrophobic frit) and concentrated *in vacuo* to afford an orange solid. The crude product was purified by FCC (silica, 0–100% EtOAc(+1% triethylamine):cyclohexane) to yield the title compound (355 mg, 1.1 mmol, 21%) as an orange solid. **LC-MS** (HpH modifier): t_r = 0.61 mins, 93% by UV, $[M+H]^+$ found 339.0. **^{1}H NMR** (400 MHz, DMSO-d_6): δ 7.75–7.72 (2H, m, 2 × ArCH), 7.43–7.39 (2H, m, 2 × ArCH), 7.26 (2H, s, 1 × NH_2), 3.33 (2H, d, *J* = 2.5 Hz, 1 × CH_2), 3.02 (1H, t, *J* = 2.5 Hz, 1 × CH), 2.84–2.80 (2H, m, 1 × CH_2), 2.79–2.77 (2H, m, 1 × CH_2). **^{13}C NMR** (101 MHz, DMSO-d_6): δ 145.3 (1C), 142.3 (1C), 129.5 (2C), 126.1 (2C), 83.4 (1C), 74.0 (1C), 49.6 (1C), 37.7 (1C), 35.6 (1C). **HRMS** (ESI, positive ion mode): *m/z* for $[C_{11}H_{14}N_2O_2S+H]^+$ = 239.0854, found 239.0858. **IR** ν_{max} (cm^{-1}): 3293, 3272, 1659, 1307.

_N_-(3-methoxybenzyl)prop-2-yn-1-amine, **10**

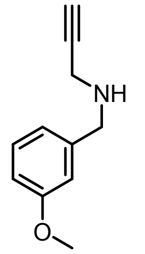

To a solution of 3-methoxybenzylamine (0.94 mL, 7.3 mmol) in DMF (20 mL) was added potassium carbonate (2120 mg, 15 mmol) and propargyl bromide (80 wt. % in toluene) (1140 mg, 7.7 mmol). The solution was stirred for 48 h at room temperature. Aqueous sodium bicarbonate solution (50 mL) and aqueous 5% lithium chloride solution (50 mL) were added, and the aqueous phase was extracted with EtOAc (3 × 100 mL). The combined organic extracts were dried (hydrophobic frit) and concentrated *in vacuo* to afford an orange gum. The crude product was purified by FCC (silica, 30–100% EtOAc(+1% triethylamine):cyclohexane) to yield the title compound (113 mg, 0.65 mmol, 9%) as a yellow oil. **LC-MS** (HpH modifier): t_r = 0.86 mins, 99% by UV, [M+H]$^+$ found 176.0. **^{1}H NMR** (400 MHz, DMSO-d_6): δ 7.25–7.19 (1H, m, 1 × ArCH), 6.92–6.87 (1H, m, 1 × ArCH), 6.91–6.89 (1H, m, 1 × ArCH), 6.82–6.78 (1H, m, 1 × ArCH), 3.75 (3H, s, 1 × CH_3), 3.72 (2H, s, 1 × CH_2), 3.29 (2H, d, *J* = 2.4 Hz, 1 × CH_2), 3.07 (1H, t, *J* = 2.4 Hz, 1 × CH). **^{13}C NMR** (101 MHz, DMSO-d_6): δ 159.7 (1C), 142.2 (1C), 129.6 (1C), 120.7 (1C), 113.9 (1C), 112.7 (1C), 83.3 (1C), 74.2 (1C), 55.4 (1C), 51.7 (1C), 37.1 (1C). **HRMS** (ESI, positive ion mode): *m/z* for $[C_{11}H_{13}NO+H]^+$ = 176.1075, found 176.1068. **IR** ν_{max} (cm^{-1}): 3287, 2835, 1262.

4-(2-Oxo-2-(prop-2-yn-1-yl(3-sulfamoylbenzyl)amino)ethyl)benzenesulfonyl fluoride, **2j**

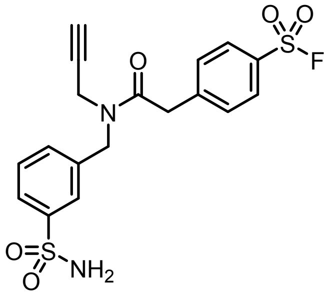

2-(4-(Fluorosulfonyl)phenyl)acetic acid (12 mg, 0.055 mmol), 3-((prop-2-yn-1-ylamino)methyl)benzenesulfonamide (16 mg, 0.071 mmol), and HATU (36 mg, 0.093 mmol) were dissolved in DMF (1.0 mL). To this solution was added DIPEA (6.3 µL, 0.036 mmol). The solution was stirred for 30 mins at room temperature. The crude reaction mixture was directly purified by MDAP (formic acid modifier) to yield the title compound (18 mg, 0.041 mmol, 75%) as a white solid.**LC-MS** (formic acid modifier): t_r = 0.96 mins, >99% by UV, [M+H]$^+$ found 424.9. **^{1}H NMR** (400 MHz, DMSO-d_6): δ 8.90, 8.06 (2H, d, *J* = 8.4 Hz, 2 × ArCH, 2 rotamers), 7.80–7.76, 7.75–7.72 (1H, m, 1 × ArCH), 7.68, 7.60 (2H, d, *J* = 8.4 Hz, 2 × ArCH, 2 rotamers), 7.59–7.58, 7.57–7.56 (1H, m, 1 × ArCH, 2 rotamers), 7.55–7.54, 7.53–7.52 (1H, m, 1 × ArCH, 2 rotamers), 7.51–7.50, 7.49–7.48 (1H, m, 1 × ArCH, 2 rotamers), 7.39, 7.34 (2H, s, 1 × NH_2, 2 rotamers), 4.89, 4.67 (2H, s, 1 × CH_2, 2 rotamers), 4.33, 4.17 (2H, d, *J* = 2.0 Hz, 1 × CH_2, 2 rotamers), 4.13, 3.99 (2H, s, 1 × CH_2, 2 rotamers), 3.36, 3.19 (1H, t, *J* = 2.0 Hz, 1 × CH, 2 rotamers). **^{13}C NMR** (101 MHz, DMSO-d_6): δ 170.2, 170.0 (1C, 2 rotamers), 145.7, 145.6 (1C, 2 rotamers), 145.0, 144.8 (1C, 2 rotamers), 138.8, 138.4 (1C, 2 rotamers), 132.2, 132.1 (2C, 2 rotamers), 131.4, 130.7 (1C, 2 rotamers), 130.2, 130.0 (1C, 2 rotamers), 129.8, 129.5 (1C, 2 rotamers), 128.7, 128.6 (2C, 2 rotamers), 125.3, 125.2 (1C, 2 rotamers), 125.0, 124.3 (1C, 2 rotamers), 79.7, 79.6 (1C, 2 rotamers), 76.2, 75.1 (1C, 2 rotamers), 50.5, 49.2 (1C, 2 rotamers), 40.1, 40.6, (1C, 2 rotamers), 38.0, 35.3 (1C, 2 rotamers). **^{19}F NMR** (376 MHz, DMSO-d_6): δ 66.6 (1F, s). **HRMS** (ESI, positive ion mode): *m/z* for $[C_{18}H_{17}FN_2O_5S_2+H]^+$ = 425.0641, found 425.0660. **IR** ν_{max} (cm^{-1}): 3287, 1643, 1403.

4-(2-Oxo-2-(prop-2-yn-1-yl(4-sulfamoylphenethyl)amino)ethyl)benzenesulfonyl fluoride, **2k**

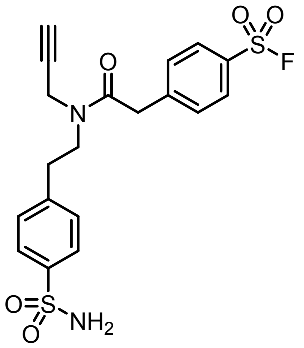

2-(4-(Fluorosulfonyl)phenyl)acetic acid (80 mg, 0.37 mmol), 4-(2-(prop-2-yn-1-ylamino)ethyl)benzenesulfonamide (114 mg, 0.48 mmol), and HATU (237 mg, 0.62 mmol) were dissolved in DMF (1.2 mL). To this solution was added DIPEA (42 µL, 0.24 mmol). The solution was stirred for 30 mins at room temperature. The crude reaction mixture was directly purified by MDAP (formic acid modifier). The isolated material was subsequently re-purified by FCC (silica, 0–100% EtOAc:cyclohexane) to yield the title compound (68 mg, 0.15 mmol, 42%) as a white solid. **LC-MS** (formic acid modifier): t_r = 0.96 mins, 99% by UV, [M+H]$^+$ found 438.9. **^{1}H NMR** (400 MHz, DMSO-d_6): δ 8.08, 8.05 (2H, d, *J* = 8.4 Hz, 2 × ArCH, 2 rotamers), 7.81, 7.75 (2H, d, *J* = 8.4 Hz, 2 × ArCH, 2 rotamers), 7.59, 7.53 (2H, d, *J* = 7.9 Hz, 2 × ArCH, 2 rotamers), 7.43, 7.42 (2H, d, *J* = 7.9 Hz, 2 × ArCH, 2 rotamers), 7.36, 7.29 (2H, s, 1 × NH_2, 2 rotamers), 4.35, 4.25 (2H, d, *J* = 2.4, 1 × CH_2, 2 rotamers), 4.00, 3.76 (2H, s, 1× CH_2, 2 rotamers), 3.73–3.61 (2H, m, 1 × CH_2, 2 rotamers), 3.38, 3.22 (1H, t, *J* = 2.4, 1 × CH, 2 rotamers), 3.04, 2.91 (2H, t, *J* = 7.1 Hz, 1 × CH_2, 2 rotamers). **^{13}C NMR** (101 MHz, DMSO-d_6): δ 169.6, 169.3 (1C, 2 rotamers), 145.9, 145.8 (1C, 2 rotamers), 143.6, 143.2 (1C, 2 rotamers), 143.0, 142.7 (1C, 2 rotamers), 132.2, 132.1 (2C, 2 rotamers), 130.2, 130.1, (2C, 2 rotamers), 129.7, 129.6 (1C, 2 rotamers), 128.6, 128.5 (2C, 2 rotamers), 126.3, 126.2 (2C, 2 rotamers), 80.6, 80.2 (1C, 2 rotamers), 75.8, 74.6 (1C, 2 rotamers), 48.7, 47.7 (1C, 2 rotamers), 39.1, 38.0 (1C, 2 rotamers), 34.7, 34.6 (1C, 2 rotamers), 34.0, 33.4 (1C, 2 rotamers). **^{19}F NMR** (376 MHz, DMSO-d_6): δ 66.6 (1F, s). **HRMS** (ESI, positive ion mode): *m/z* for $[C_{19}H_{19}FN_2O_5S_2+H]^+$ = 439.0798, found 439.0798. **IR** ν_{max} (cm^{-1}): 3295, 1635, 1400, 1309.

4-(2-((3-Methoxybenzyl)(prop-2-yn-1-yl)amino)-2-oxoethyl)benzenesulfonyl fluoride, **2l**

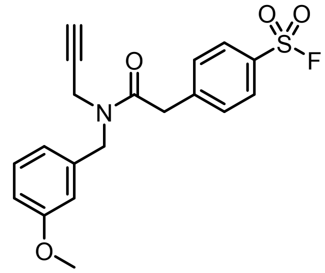

2-(4-(Fluorosulfonyl)phenyl)acetic acid (65 mg, 0.30 mmol), *N*-(3-methoxybenzyl)prop-2-yn-1-amine (67.9 mg, 0.39 mmol), and HATU (193 mg, 0.51 mmol) were dissolved in DMF (1.0 mL). To this solution was added DIPEA (34 µL, 0.20 mmol). The solution was stirred for 30 mins at room temperature. The crude reaction mixture was directly purified by MDAP (formic acid modifier) to yield the title compound (74 mg, 0.20 mmol, 66%) as a yellow oil. **LC-MS** (formic acid modifier): t_r = 1.17 mins, 98% by UV, $[M+H]^+$ found 375.9. **^{1}H NMR** (400 MHz, DMSO-d_6): δ 8.09, 8.05 (2H, d, *J* = 8.4 Hz, 2 × ArCH, 2 rotamers), 7.68, 7.59 (2H, d, *J* = 8.4 Hz, 2 × ArCH, 2 rotamers), 7.31–7.28, 7.27–7.23 (1H, m, 1 × ArCH, 2 rotamers), 6.90–6.87, 6.87–6.86 (1H, m, 1 × ArCH, 2 rotamers), 6.85–6.84, 6.84–6.82 (1H, m, 1 × ArCH, 2 rotamers), 6.81–6.80, 6.80–6.78 (1H, m, 1 × ArCH, 2 rotamers), 4.76, 4.57 (2H, s, 1 × CH_2, 2 rotamers), 4.28, 4.14 (2H, d, *J* = 2.1 Hz, 1 × CH_2, 2 rotamers), 4.11, 3.98 (2H, s, 1 × CH_2, 2 rotamers), 3.74, 3.73 (3H, s, 1 × CH_3, 2 rotamers), 3.36, 3.19 (1H, t, *J* = 2.1 Hz, 1 × CH, 2 rotamers). **^{13}C NMR** (101 MHz, DMSO-d_6): δ 170.0, 169.9 (1C, 2 rotamers), 160.1, 159.8 (1C, 2 rotamers), 145.9, 145.7 (1C, 2 rotamers), 139.2, 138.8 (1C, 2 rotamers), 132.2, 131.9 (2C, 2 rotamers), 130.3, 130.2 (1C, 2 rotamers), 130.0, 129.9 (1C, 2 rotamers), 128.7, 128.6 (2C, 2 rotamers), 120.3, 119.4 (1C, 2 rotamers), 113.7, 113.4 (1C, 2 rotamers), 113.1, 112.9 (1C, 2 rotamers), 80.0, 79.7 (1C, 2 rotamers), 75.9, 74.9 (1C, 2 rotamers), 55.5, 55.4 (1C, 2 rotamers), 50.9, 49.1 (1C, 2 rotamers), 39.6, 39.5 (1C, 2 rotamers), 37.7, 35.3 (1C, 2 rotamers). **^{19}F NMR** (376 MHz, DMSO-d_6): δ 66.5 (1F, s). **HRMS** (ESI, positive ion mode): *m/z* for $[2(C_{19}H_{18}FNO_4S)+Na]^+$ = 773.1779, found 773.1780. **IR** ν_{max} (cm^{-1}): 1647, 1599, 1403.

4-(2-((3-(3,3-Dimethylureido)benzyl)amino)-2-oxoethyl)benzenesulfonyl fluoride, 2f

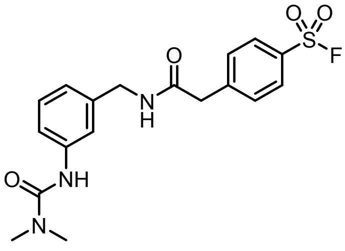

This compound was supplied by Enamine.

4-(2-((3-Acetamidobenzyl)amino)-2-oxoethyl)benzenesulfonyl fluoride, 2g

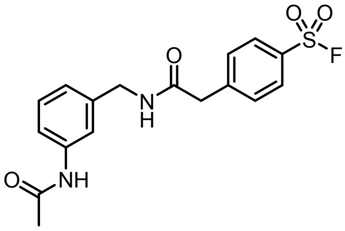

This compound was supplied by Enamine.

4-(2-(3-(3,4-Difluorophenoxy)azetidin-1-yl)-2-oxoethyl)benzenesulfonyl fluoride, **2h**

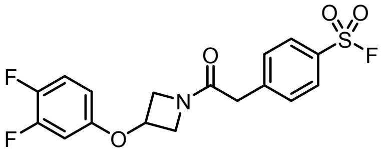

2-(4-(Fluorosulfonyl)phenyl)acetic acid (80 mg, 0.37 mmol), 3-(3,4-difluorophenoxy)azetidine (81 mg, 0.37 mmol), and HATU (237 mg, 0.62 mmol) were dissolved in DMF (1.1 mL). To this solution was added DIPEA (96 mL, 0.55 mmol). The solution was stirred for 1 h at room temperature. The crude reaction mixture was directly purified by MDAP (formic acid modifier). The solvent was dried under a stream of nitrogen to yield the title compound (58 mg, 0.15 mmol, 41%) as a colorless gum. **LC-MS** (formic acid modifier): t_r = 1.15 mins, 97% by UV, $[M+H]^+$ found 385.9. **^{1}H NMR** (400 MHz, DMSO-d_6): δ 8.08 (2H, d, *J* = 8.4 Hz, 2 × ArCH), 7.67 (2H, d, *J* = 8.4 Hz, 2 × ArCH), 7.43–7.35 (1H, m, 1 × ArCH), 7.04 (1H, ddd, *J* = 12.4, 6.5, 3.2 Hz, 1 × ArCH), 6.74–6.70 (1H, m, 1 × ArCH), 5.09–5.04 (1H, m, 1 × CH), 4.70–4.66 (1H, m, 1 × CH), 4.37–4.32 (1H, m, 1 × CH), 4.23–4.20 (1H, m, 1 × CH), 3.85–3.79 (1H, m, 1 × CH), 3.72 (2H, s, 1 × CH_2). **^{13}C NMR** (151 MHz, DMSO-d_6): δ 169.5 (1C, s), 153.3 (1C, dd, *J* =9, 2 Hz), 150.2 (1C, dd, *J* = 245, 14 Hz), 146.0–144.0 (1C, m), 145.5 (1C, s), 132.0 (2C, s), 130.1 (1C, d, *J* = 23 Hz), 128.7 (2C, s), 118.4 (1C, d, *J* = 18 Hz), 111.4 (1C, dd, *J* = 6, 3 Hz), 105.0 (1C, d, *J* = 20 Hz), 66.5 (1C, s), 57.3 (1C, s), 55.0 (1C, s), 38.0 (1C, s). **^{19}F{^{13}C} NMR** (376 MHz, DMSO-d_6): δ 66.6 (1F, s, 1 × SO_2F), -135.9 (1F, d, *J* = 22.9 Hz, 1 × ArCF), -148.03 (1F, d, *J* = 22.9 Hz, 1 × ArCF). **HRMS** (ESI, positive ion mode): *m/z* for $[C_{17}H_{14}F_3NO_4S+H]^+$ = 386.0674, found 386.0669. **IR** ν_{max} (cm^{-1}): 1650, 1517, 1442.

4-(2-(3-(2-Carbamoylphenoxy)azetidin-1-yl)-2-oxoethyl)benzenesulfonyl fluoride, **2i**

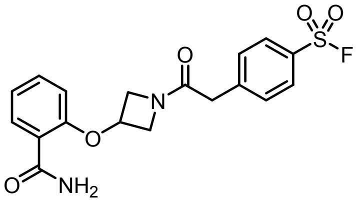

2-(4-(Fluorosulfonyl)phenyl)acetic acid (71 mg, 0.33 mmol), 2-(azetidin-3-yloxy)benzamide (89 mg, 0.39 mmol), and HATU (247 mg, 0.65 mmol) were dissolved in DMF (1.1 mL). To this solution was added DIPEA (91 mL, 0.52 mmol). The solution was stirred for 1 h at room temperature. The crude reaction mixture was directly purified by MDAP (formic acid modifier). The solvent was dried under a stream of nitrogen to afford a pale orange gum. Saturated sodium bicarbonate solution (20 mL) was added, and the aqueous phase was extracted with DCM (3 × 20 mL). The combined organic extracts were dried (hydrophobic frit) and concentrated *in vacuo* to yield the title compound (47.9 mg, 0.12 mmol, 38%) as a colorless gum. **LC-MS** (formic acid modifier): t_r = 0.86 mins, >99% by UV, $[M+H]^+$ found 392.9. **^{1}H NMR** (400 MHz, DMSO-d_6): δ 8.09 (2H, d, J = 8.4 Hz, 2 × ArC$\underline{H}$), 7.78 (1H, dd, J = 7.88, 1.97 Hz, 1 × ArC$\underline{H}$), 7.68 (2H, d, J = 8.4 Hz, 2 × ArC$\underline{H}$), 7.55 (2H, s, 1 × N$\underline{H_2}$), 7.48–7.43 (1H, m, 1 × ArC$\underline{H}$), 7.11–7.06 (1H, m, 1 × ArC$\underline{H}$), 6.87–6.83 (1H, m, 1 × ArC$\underline{H}$), 5.20–5.13 (1H, m, 1 × C$\underline{H}$), 4.73–4.67 (1H, m, 1 × C$\underline{H}$), 4.39–4.36 (1H, m, 1 × C$\underline{H}$), 4.36–4.32 (1H, m, 1 × C$\underline{H}$), 4.00–3.94 (1H, m, 1 × C$\underline{H}$), 3.73 (2H, s, 1 × C$\underline{H_2}$). **^{13}C NMR** (101 MHz, DMSO-d_6): δ 169.6 (1C), 166.8 (1C), 154.4 (1C), 145.6 (1C), 132.7 (1C), 132.0 (2C), 131.4 (1C), 130.1 (1C), 128.7 (2C), 124.5 (1C), 121.7 (1C), 113.2 (1C), 66.4 (1C), 55.3 (2C), 38.1 (1C). **^{19}F NMR** (376 MHz, DMSO-d_6): δ 66.6 (1F, s). **HRMS** (ESI, positive ion mode): m/z for $[C_{18}H_{17}FN_2O_5S+H]^+$ = 393.0920, found 393.0921. **IR** ν_{max} (cm^{-1}): 3392, 3180, 1645, 1602.

4-(2-(4-(2-Carbamoylphenoxy)piperidin-1-yl)-2-oxoethyl)benzenesulfonyl fluoride, **2p**

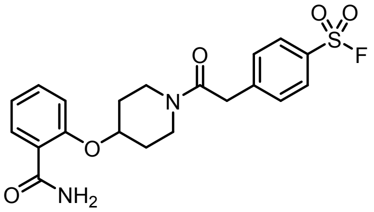

2-(4-(Fluorosulfonyl)phenyl)acetic acid (80 mg, 0.37 mmol), 2-(piperidin-4-yloxy)benzamide (89 mg, 0.39 mmol), and HATU (237 mg, 0.62 mmol) were dissolved in DMF (1.1 mL). To this solution was added DIPEA (96 mL, 0.55 mmol). The solution was stirred for 1 h at room temperature. The crude reaction mixture was directly purified by MDAP (formic acid modifier). The solvent was dried under a stream of nitrogen to yield the title compound (39.1 mg, 0.093 mmol, 25%) as a colorless gum. **LC-MS** (formic acid modifier): t_r = 0.93 mins, 95% by UV, $[M+H]^+$ found 421.0. **^{1}H NMR** (400 MHz, DMSO-d_6): δ 8.08 (2H, d, J = 8.4 Hz, 2 × ArC$\underline{H}$), 7.76–7.74 (1H, dd, J = 7.88, 1.97 Hz, 1 × ArC$\underline{H}$), 7.65 (2H, d, J = 8.4 Hz, 2 × ArC$\underline{H}$), 7.51 (2H, s, 1 × N$\underline{H_2}$), 7.48–7.42 (1H, m, 1 × ArC$\underline{H}$), 7.25–7.21 (1H, m, 1 × ArC$\underline{H}$), 7.06–7.00 (1H, m, 1 × ArC$\underline{H}$), 4.84–4.77 (1H, m, 1 × C$\underline{H}$), 3.99 (2H, s, 1 × C$\underline{H_2}$), 3.87–3.73 (2H, m, 2 × C$\underline{H}$), 3.51–3.33 (2H, m, 2 × C$\underline{H}$), 2.01–1.91 (2H, m, 2 × C$\underline{H}$), 1.79–1.62 (2H, m, 2 × C$\underline{H}$). **^{13}C NMR** (101 MHz, DMSO-d_6): δ 168.1 (1C), 137.2 (1C), 155.1 (1C), 146.5 (1C), 132.5 (1C), 131.9 (2C), 131.2 (1C), 130.0 (1C), 128.6 (2C), 125.1 (1C), 121.2 (1C), 115.2 (1C), 73.4 (1C), 42.9 (2C), 39.0 (1C), 30.5 (2C). **^{19}F NMR** (376 MHz, DMSO-d_6): δ 66.6 (1F, s). **HRMS** (ESI, positive ion mode): m/z for $[C_{20}H_{21}FN_2O_5S+H]^+$ = 421.1233, found 421.1231. **IR** ν_{max} (cm^{-1}): 3464, 1630, 1401.

4-(2-(4-(2,4-Difluorophenoxy)piperidin-1-yl)-2-oxoethyl)benzenesulfonyl fluoride, **2q**

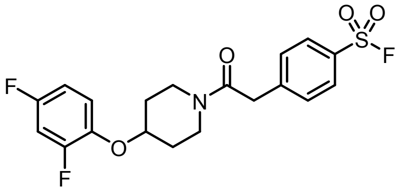

This compound was supplied by Enamine.

4-(2-((3-(Cyclopropanecarboxamido)benzyl)amino)-2-oxoethyl)benzenesulfonyl fluoride, **2s**

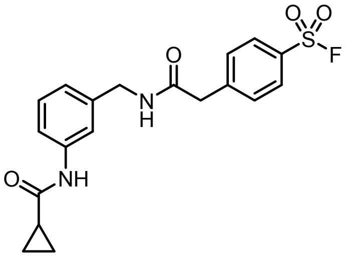

This compound was supplied by Enamine.

4-(2-(4-((2-Fluorophenyl)amino)piperidin-1-yl)-2-oxoethyl)benzenesulfonyl fluoride, **2t**

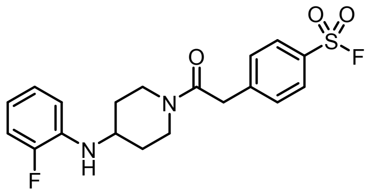

2-(4-(Fluorosulfonyl)phenyl)acetic acid (80 mg, 0.37 mmol), *N*-(2-fluorophenyl)piperidin-4-amine (78 mg, 0.40 mmol), and HATU (237 mg, 0.62 mmol) were dissolved in DMF (1.1 mL). To this solution was added DIPEA (96 mL, 0.55 mmol). The solution was stirred for 1 h at room temperature. The crude reaction mixture was directly purified by MDAP (formic acid modifier). The solvent was dried under a stream of nitrogen to yield the title compound (89.9 mg, 0.228 mmol, 62%) as a white solid. **LC-MS** (formic acid modifier): t_r = 1.16 mins, 97% by UV, $[M+H]^+$ found 394.9. **^{1}H NMR** (400 MHz, DMSO-d_6): δ 8.08 (2H, d, *J* = 8.4 Hz, 2 × ArCH), 7.65 (2H, d, *J* = 8.4 Hz, 2 × ArCH), 7.10–7.01 (1H, m, 1 × ArCH), 6.44–6.41 (1H, m, 1 × ArCH), 6.40–6.35 (1H, m, 1 × ArCH), 6.30–6.23 (1H, m, 1 × ArCH), 5.85 (1H, d, *J* = 8.4 Hz, 1 × NH), 4.30–4.20 (1H, m, 1 × CH), 3.97 (2H, s, 1 × CH_2), 3.96–3.90 (1H, m, 1 × CH), 3.55–3.43 (1H, m, 1 × CH), 3.28–3.19 (1H, m, 1 × CH), 2.93–2.84 (1H, m, 1 × CH), 1.96–1.86 (2H, m, 2 × CH), 1.31–1.16 (2H, m, 2 × CH). **^{13}C NMR** (151 MHz, DMSO-d_6): δ 168.0 (1C, s), 164.1 (1C, d, *J* = 239 Hz), 150.2 (1C, d, *J* = 11 Hz), 146.6 (1C, s), 131.8 (2C, s), 130.7 (1C, d, *J* = 11 Hz), 129.9 (1C, d, *J* = 11 Hz), 128.7 (2C, s), 109.2 (1C, br s), 102.0 (1C, br d, *J* = 21 Hz), 98.9 (1C, br d, *J* = 25 Hz), 49.0 (1C, s), 44.5 (1C, s), 40.6 (1C, s), 39.7 (1C, s), 32.4 (1C, s), 31.7 (1C, s). **^{19}F NMR** (376 MHz, DMSO-d_6): δ 66.6 (1F, s, 1 × SO_2F), -113.2 (1F, s, 1 × ArCF). **HRMS** (ESI, positive ion mode): *m/z* for $[C_{19}H_{20}F_2N_2O_3S+H]^+$ = 395.1241, found 395.1241. **IR** ν_{max} (cm^{-1}): 3324, 1619, 1415, 1203.

10. References

(1) (2020) R Studio 3.6.3, RSutio Team: Integrated Fevelopment for R. RStudio, PBC, Boston, MA, www.rstudio.com.

(2) Grant, E. K., Fallon, D. J., Hann, M. M., Fantom, K. G. M., Quinn, C., Zappacosta, F., Annan, R. S., Chung, C. wa, Bamborough, P., Dixon, D. P., Stacey, P., House, D., Patel, V. K., Tomkinson, N. C. O., and Bush, J. T. (2020) A Photoaffinity-Based Fragment-Screening Platform for Efficient Identification of Protein Ligands. *Angew. Chem. Int. Ed. 59*, 21096–21105.

(3) Thomas, R. P., Heap, R. E., Zappacosta, F., Grant, E. K., Pogány, P., Besley, S., Fallon, D. J., Hann, M. M., House, D., Tomkinson, N. C. O., and Bush, J. T. (2021) A direct-to-biology high-throughput chemistry approach to reactive fragment screening. *Chem. Sci. 12*, 12098–12106.

(4) (2020) Pipeline Pilot 20.1.0.2208, BIOVIA, Dassault Systèmes, San Diego.

(5) Leo, A. J., and Hoekman, D. (2000) Calculating log P(oct) with no missing fragments; the problem of estimating new interaction parameters. *Perspect. Drug Discov. Des. 18*, 19–38.

(6) Szegezdi, J., and Csizmadia, F. (2007) A method for calculating the pKa values of small and large molecules, in *Chemaxon*.

(7) Csepregi, S., Vargyas, M., Papp, Á., Csizmadia, F., Papp, J., and Vadász, P. (2008) Maximum Common Substructure Based Hierarchical Clustering, in *Chemaxon*.

(8) Rogers, D., and Hahn, M. (2010) Extended-Connectivity Fingerprints. *J. Chem. Inf. Model. 50*, 742–754.

(9) Perkins, D. N., Pappin, D. J. C., Creasy, D. M., and Cottrell, J. S. (1999) Probability-based protein identification by searching sequence databases using mass spectrometry data. *Electrophoresis 20*, 3551–3567.

(10) (2019) Molecular Operating Environment (MOE) 2019.01, Chemical Computing Group ULC, 1010 Sherbooke St. West, Suite #910, Montreal, QC, Canada, H3A 2R7.

(11) GraphPad Prism 5.0.4, GraphPad Software, San Diego, California, USA, www.graphpad.com. GraphPad Software, San Diego, California USA, www.graphpad.com.

(12) Bruderer, R., Bernhardt, O. M., Gandhi, T., Miladinović, S. M., Cheng, L. Y., Messner, S., Ehrenberger, T., Zanotelli, V., Butscheid, Y., Escher, C., Vitek, O., Rinner, O., and Reiter, L. (2015) Extending the limits of quantitative proteome profiling with data-independent acquisition and application to acetaminophen-treated three-dimensional liver microtissues. *Mol. Cell. Proteomics 14*, 1400–1410.

(13) Perez-Riverol, Y., Csordas, A., Bai, J., Bernal-Llinares, M., Hewapathirana, S., Kundu, D. J., Inuganti, A., Griss, J., Mayer, G., Eisenacher, M., Pérez, E., Uszkoreit, J., Pfeuffer, J., Sachsenberg, T., Yilmaz, Ş., Tiwary, S., Cox, J., Audain, E., Walzer, M., Jarnuczak, A. F., Ternent, T., Brazma, A., and Vizcaíno, J. A. (2019) The PRIDE database and related tools and resources in 2019: Improving support for quantification data. *Nucleic Acids Res. 47*, 442–450.

(14) Kabsch, W. (2010) XDS. *Acta Crystallogr. Sect. D Biol. Crystallogr. 66*, 125–132.

(15) Evans, P. R. (1993) "Data reduction" - Data Collection & Processing, in *Proceedings of CCP4 Study Weekend*, pp 114–122.

(16) Bricogne, G., Blanc, E., Brandl, M., Flensburg, C., Keller, P., Paciorek, W., Roversi, P., Sharff, A., Smart, O. S., Vonrhein, C., and Womack, T. O. (2017) BUSTER 2.11.8, Global Phasing Ltd., Cambridge, United Kingdom.

(17) Vonrhein, C., Flensburg, C., Keller, P., Sharff, A., Smart, O., Paciorek, W., Womack, T., and Bricogne, G. (2011) Data processing and analysis with the autoPROC toolbox. *Acta Crystallogr. Sect. D Biol. Crystallogr. 67*, 293–302.

(18) Smart, O. S., Womack, T. O., Sharff, A., Flensburg, C., Keller, P., Paciorek, W., Vonrhein, C., and Briscogne, G. (2011) Grade 1.2.20, Global Phasing Ltd., Cambridge, United Kingdom, www.globalphasing.com.

(19) Emsley, P., Lohkamp, B., Scott, W. G., and Cowtan, K. (2010) Features and development of Coot. *Acta Crystallogr. Sect. D Biol. Crystallogr. 66*, 486–501.

(20) Collaborative Computational Project and IUCr. (1994) The CCP4 suite: Programs for protein crystallography. *Acta Crystallogr. Sect. D Biol. Crystallogr. 50*, 760–763.

(21) Murshudov, G. N., Vagin, A. A., and Dodson, E. J. (1997) Refinement of macromolecular structures by the maximum-likelihood method. *Acta Crystallogr. Sect. D Biol. Crystallogr. 53*, 240–255.
